# Diversity Scaling of Human Digestive Tract (DT) Microbiomes: The Intra-DT and Inter-individual Patterns

**DOI:** 10.3389/fgene.2021.724661

**Published:** 2021-09-24

**Authors:** Hongju (Daisy) Chen, Bin Yi, Qiang Liu, Xia Xu, Lin Dai, Zhanshan (Sam) Ma

**Affiliations:** ^1^ Computational Biology and Medical Ecology Lab, State Key Laboratory of Genetic Resources and Evolution, Kunming Institute of Zoology, Chinese Academy of Sciences, Kunming, China; ^2^ Kunming College of Life Sciences, University of Chinese Academy of Sciences, Kunming, China; ^3^ College of Mathematics, Honghe University, Mengzi, China; ^4^ Faculty of Science, Kunming University of Science and Technology, Kunming, China; ^5^ Center for Excellence in Animal Evolution and Genetics, Chinese Academy of Sciences, Kunming, China

**Keywords:** Inter-individual microbiome heterogeneity, Intra-DT microbiome heterogeneity, Diversity-area relationship (DAR), Potential diversity, Diversity scaling

## Abstract

The human gut microbiome has been extensively studied, but its diversity scaling (changes or heterogeneities) along the digestive tract (DT) as well as their inter-individual heterogeneities have not been adequately addressed to the best of our knowledge. Here we fill the gap by applying the diversity-area relationship (DAR), a recent extension to the classic species-area relationship (SAR) in biogeography, by reanalyzing a dataset of over 2000 16s-rRNA microbiome samples obtained from 10 DT sites of over 200 individuals. We sketched out the biogeography “maps” for each of the 10 DT sites by cross-individual DAR analysis, and the intra-DT distribution pattern by cross-DT-site DAR analysis. Regarding the inter-individual biogeography, it was found that all DT sites have the invariant (constant) scaling parameter—all sites possessing the same diversity change rate across individuals, but most sites have different *potential* diversities, which include the portions of diversity that may be absent locally but present regionally. In the case of this study, the potential diversity of each DT site covers the total diversity of the respective site from all individuals in the cohort. In terms of the genus *richness*, an average individual hosts approximately 20% of the population-level genus richness (total bacterial genus of a human population). In contrast, in terms of community *biodiversity*, the percentages of individual over population may exceed 90%. This suggests that the differences between individuals in their DT microbiomes are predominantly in the composition of bacterial species, rather than how their abundances are distributed (*i.e*., biodiversity). Regarding the intra-DT patterns, the scaling parameter (*z*) is larger—suggesting that the intra-DT biodiversity changes are larger than inter-individual changes. The higher intra-DT heterogeneity of bacteria diversity, as suggested by larger intra-DT *z* than the inter-individual heterogeneity, should be expected since the intra-DT heterogeneity reflects the functional differentiations of the DT tract, while the inter-individual heterogeneity (z) reflects the difference of the same DT site across individuals. On average, each DT site contains 21–36% of the genus diversity of the whole DT, and the percentages are even higher in terms of higher taxon levels.

## Introduction

There have been extensive studies on various aspects of human gut microbiomes over the last decade or so, particularly on their diversities (for example, HMP Consortium (Human Microbiome Project Consortium), 2012a; HMP Consortium (Human Microbiome Project Consortium), 2012b; Lozupone et al., 2012; Segata et al., 2012). However, certain information on the diversity scaling (changes) across individuals or across various sites of an individual’s DT (digestive tract) seems to be still missing. For example, to what extent can an individual’s gut microbial species richness (number of species) represent a population? What about species diversity (such as Shannon evenness index) if individual *vs* population is compared? How is the microbiome distributed along one’s DT, in terms of species richness or diversity? How about the scaling relationships on higher taxon levels, such as phylum, class, order, and family? Similar questions are traditionally investigated in biogeography. One of the most well-known ecological laws in the field is the so-termed species-area relationship (SAR), which was first discovered in the 19th century (Watson, 1835) and has been extensively investigated since the 1960s (Preston, 1960; Connor and McCoy, 1979; Rosenzweig, 1995; Lomolino, 2000; Drakare et al., 2006; Tjørve and Tjørve, 2008, 2009; Harte et al., 2009; He and Hubbell 2011; Sizling et al., 2011; Storch et al., 2012; Triantis et al., 2012; Helmus et al., 2014). It is considered as “ecology’s most general, yet protean pattern” by Lomolino (2000) and Whittaker and Triantis (2012). In practice, SAR has become one of the most important theories and models in conservation biology and biodiversity protection. This is because SAR can be applied to establish a simple functional relationship between the number of species (species richness) and the area of a region. It was found that SAR typically follow a simple power function in the form of *S* = *c*A^z^, where *S* is the number of species, *A* is the size of area (in the case of human microbiome, *A* can be treated as the number of individuals sampled), and *z* and *c* are SAR parameters. In particular, *z* is termed the scaling parameter of SAR, and it is a measure of change (increase) rate of species number over area.

It is generally recognized that the number of species, or formally species richness, is a rather convenient but very rough measure of biodiversity. This is because biodiversity is obviously strongly influenced by both species richness and species abundances of individual species. Several biodiversity metrics that consider both species numbers and abundances have been proposed and widely used in ecology since 1960s. A recent consensus has been that the Hill numbers, which were first introduced by Hill (1973) into ecology but did not receive significant attention until recently, offer the most appropriate metrics for measuring alpha-diversity (Chao et al., 2012; Chao et al., 2014a; Chao et al., 2014b) since most existing diversity metrics such as species richness, Shannon entropy, and Simpson index turned out to be special cases (or functions) of the Hill numbers. To take advantages of the Hill numbers as general biodiversity metrics, Ma (2018a) extended the classic SAR to more general diversity-area relationship (DAR) by substituting the species richness with general diversity measured with Hill numbers. In the present study, we applied the DAR approach to address the previous raised questions regarding the changes of DT microbiome diversity, both across individuals (inter-individual) and across DT sites (intra-individual or intra-DT).

To investigate the inter-individual and intra-individual (intra-DT) diversity scaling patterns with the DAR approach, we used a dataset originally collected by Segata et al (2012). Their study collected ten microbiome samples from each of over 200 individuals’ digestive tract [buccal mucosa (BM), keratinized gingiva (KG), hard palate (HP), throat (Th), palatine tonsils (PT), tongue dorsum (TD) and saliva (Sal), supraginval (SupP), subgingival plaques (SubP), and stool (Stool)]. The dataset provides an ideal opportunity for us achieve the objective of this study—analyzing the inter-individual and intra-individual diversity scaling of the human DT microbiomes.

## Materials and Methods

### A Brief Description of the Digestive Tract Microbiome Dataset

The DT microbiome dataset we reanalyzed in this study was first reported by Segata et al. (2012), which is part of the Human Microbiome Project (HMP). A total of 2078 DT microbiome samples were collected from 242 healthy adults aged from 18 to 40 years old, who were enrolled in the HMP. The ten DT sites sampled included seven from the oral cavity (BM, KG, HP, Th, PT, TD, and Sal), two from the oropharynx (SupP and SubP), and one from the gut (Stool). The operational taxonomic unit (OTU) tables and the metadata information on the individuals are available at https://www.hmpdacc.org/, and for more detailed information on the dataset, refer to Segata *et al.* (2012).

### Computational Procedures for the DAR Analysis

#### Definitions of Alpha Diversities

We applied the Hill numbers (Hill 1973; Chao et al., 2012; 2014a) to measure the alpha diversity, which are defined as:

Dq=(∑i=1Spiq)1/(1−q) (1)

where *D* is the diversity in Hill numbers, *q* (=0, 1, 2, …) is the order number of diversity, *S* is number of species (or OTUs), and *p*_*i*_ is the relative abundance of OTU *i*. When *q* = 1, the Hill number is not defined, but we can figure out its limit as *q* approaches to 1 as follows:

D1=limq→1Dq=exp(−∑i=1Spi⁡log(pi)) (2)

The Hill numbers are a series of diversity measures corresponding to different diversity orders (*q*), where *q* determines the weight of relative frequencies of species abundances. When *q* = 0, species abundance is not involved in the calculation, and ^0^ *D* is the number of OTUs or the species richness. When *q* = 1, ^1^ *D* equals the *exponential* of Shannon entropy, which represents the number of typical or common species in the community. When *q* = 2, ^2^ *D* is equal to the reciprocal of the Simpson index and represents the number of species with high abundance. Generally, ^*q*^ *D* represents the diversity of a community with *x* = ^*q*^ *D* equally abundant species.

#### DAR Analysis

According to Ma (2018a), Ma (2018b), Ma (2019), we selected and used two DAR models for the DT microbiome in this study, one is the power law (PL) model, and another is the power law with exponential cutoff (PLEC) model. The PL model is:

Dq=cAz (3)

where ^*q*^ *D* is the diversity measured in the *q*th order Hill numbers, *A* is *area*, and *c* and *z* are the PL parameters.

The PLEC model is:

Dq=cAz⁡exp(dA) (4)

where *d* is a third parameter with taper-off effect, and exp (*dA*) is the exponential decay term that eventually overwhelms the power law behavior when *A* becomes very large.

We transformed the non-linear Eqs 3, 4 into log-linear regression Equations 5, 6 to estimate the parameters of PL and PLEC models, respectively:

ln(D)=ln(c)+z⁡ln(A) (5)

ln(D)=ln(c)+z⁡ln(A)+dA (6)

#### Four Important DAR Parameters and Corresponding Profiles

According to Ma (2018a) and Ma and Li (2019), there are four important DAR parameters, including the diversity scaling parameter (*z*), pair-wise diversity overlap (PDO or *g*), maximal accrual diversity (MAD or *D*_max_), and ratio of individual diversity to population accrual diversity (RIP).

i) As the slope or tangent of the PL-DAR model (Eqs. 3, 5, the *z*-value was termed as the diversity scaling parameter.
ii) The PDO or *g* was defined as,

$$g=2-2^{z}$$ (7)

where *z* is the scaling parameter of the PL-DAR model. The range of g is generally between 0 and 1. If *z* = 1, then *g* = 0 and there is no overlap or similarity. If *z* = 0, then *g* = 1 and there is a total overlap or similarity.

iii) The MAD or *D*_max_ was defined based on the PLEC-DAR model (Eqs. 4, 6, that is,

Dmaxq=c(−zd)z⁡exp(−z)=cAmaxz⁡exp(−z) (8)

where *A*_max_ = -*z*/*d* is the number of individuals (microbiome samples) needed to reach the MAD, and *c* and *d* are parameters of the PLEC-DAR model.

iv) The RIP was defined as,

RqIP=cq/Dmax2 (9)

Where ^*q*^ *c* is the parameter of PL-DAR model at diversity order of *q*, and ^*q*^ *D*_max_ is the MAD that can be computed with Eq. 8.

Ma (2018a) and Ma and Li (2019) also defined the relationships between these four parameters and the diversity order (*q*) as the DAR profile, PDO profile, MAD profile and RIP profile, respectively.

#### Design for DAR Analysis

Our analysis consists of two parts, inter-individual (cross-individual) DAR analysis and intra-individual (cross-DT site) DAR analysis. Based on the inter-individual DAR analysis, we can investigate diversity scaling for each of the 10 DT sites across 200 + individuals. We built PL-DAR and PLEC-DAR models for each DT site, and further conducted permutation tests (randomization test) for the DAR parameters (i.e., *z* and *D*_max_) of different DT sites. The procedure for the randomization test refers to Collingridge (2013), in which the number of permutations or re-samplings was set to 1,000 times. The *p*-value of randomization test can be used to determine the significance of differences. It is noted that parameter *c* of the PL model indicates the diversity in the first unit of area to accrue. Thus, to exclude the influence of the accrual order of area unit on parameter *c*, we randomly permutated the area units to be accumulated each time the DAR model was built. In the inter-individual DAR analysis, we repeated this re-sampling procedure 100 times, and adopted the averages of the model parameters from the 100 times of DAR fittings as the final model parameters of the inter-individual DAR model for the DT site under investigation. The detailed computational procedures can be found in Ma (2018a), Ma (2018b). Based on the intra-individual DAR analysis, we can investigate intra-DT diversity scaling across 10 DT sites. The steps of intra-individual DAR analysis are as follows: 1) We first randomly selected a sample from all samples belonging to the same DT site, and a total of 10 samples from 10 DT sites constitute the intra-DAR samples for an “individual”. 2) We built PL-DAR and PLEC-DAR models for the “individual”, in which area units (DT sites) were accumulated in order of anatomy structure from the oral cavity to the intestinal tract. 3) We repeated steps (*i*)-(*ii*) 1,000 times, and adopted the averages of the model parameters from the 1,000 times of DAR fittings as the final model parameters of the intra-individual DAR model. In addition, beside genus taxon level, we also analyzed the DAR patterns of other taxa including phylum, class, order, and family taxa.

## Results

### Inter-Individual DAR Modeling of the Human Gut Microbiome for Each DT Site

**FIGURE 1:**
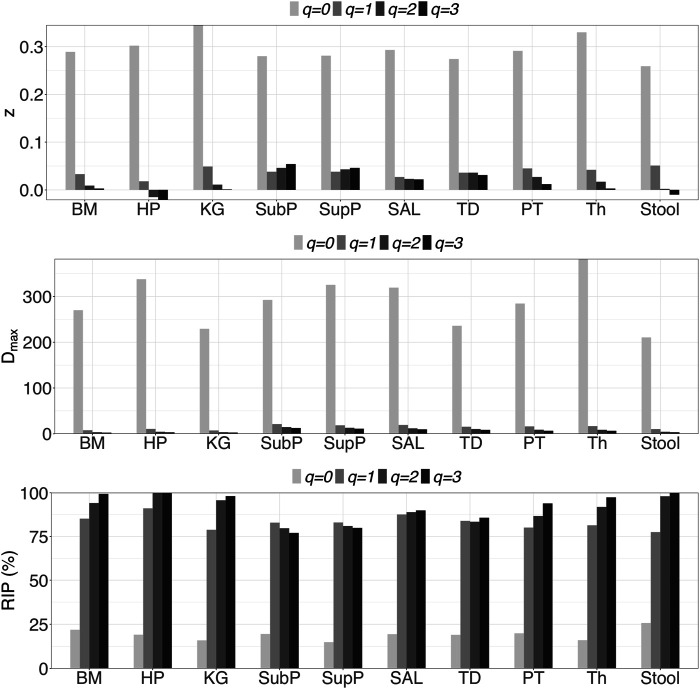
Graphs of the three important profiles from inter-individual DAR models for each of the 10 DT microbiome sites at genus taxon level, including DAR profiles (*z-q* patterns), MAD profiles (*D*_max_-*q* patterns), and RIP profiles (*RIP*-*q* patterns). Bar color-depth indicates diversity order. The *x*-axis shows the DT sites: buccal mucosa (BM), keratinized gingiva (KG), hard palate (HP), throat (Th), palatine tonsils (PT), tongue dorsum (TD) and saliva (Sal), supraginval (SupP), subgingival plaques (SubP), and stool (Stool).

At each of the five taxon levels, we built PL-DAR and PLEC-DAR models for each of the 10 sites of the human DT microbiome. Supplementary Table S1 listed the results of fitting DAR models for each 10 DT sites at the genus taxon level, including the diversity order (*q*) of the Hill numbers, the mean model parameters (*z*, *c*, *d*, *g*, *D*_max_ and RIP) and measures for goodness-of-fitting (*R* and *p*-value). *N* is the number of successful fittings out of 100 re-samplings, as explained previously. Supplementary Tables S2-S5 listed the results at other taxon levels, i.e., phylum, class, order, and family. Tables 1 and 2 listed results from the permutation tests for the differences in scaling parameter (*z*) and *D*_max_ of different DT microbiome sites. From these results, we summarize the following findings:

i) *DAR profile*: At the genus level, the average scaling parameter (*z*) of the 10 DT sites across diversity order *q* = 0–3 is *z* = (0.294, 0.038, 0.020, 0.014), and their standard errors ranged from 0.003 to 0.008 (as shown in Supplementary Table S1 and Figure 1). For each DT microbiome site, the *z*-values monotonically decreased with the diversity order *q*. As shown in Supplementary Tables S2-S5, the scaling parameter (*z*) gradually decreased with the taxon level, and for example, the average scaling parameter (*z*) at phylum level across *q* = 0–3 is *z* = (0.108, 0.013, 0.009, 0.007). As shown in Table 1, no significant differences in scaling parameter (*z*) at species-level were detected among 10 DT sites, and the detailed results of randomization tests were listed in Supplementary Table S7.
ii) *PDO profile*: PDO or parameter *g* characterizes the overlap or similarity between pair-wise microbiomes. As shown in Supplementary Table S1, at genus level, the average PDO parameter (*g*) of the 10 DT sites across diversity order *q* = 0–3 is *g* = (0.773, 0.973, 0.985, 0.989), and their standard errors ranged from 0.002 to 0.007. In contrast to the diversity scaling parameter (*z*), the PDO parameter (*g*) increased with either diversity order *q* or taxon level (see Supplementary Tables S2-S5).
iii) *MAD profile*: As shown in Supplementary Table S1, at genus level, the average *D*_max_ of the 10 DT sites across diversity order *q* = 0–3 is *D*_max_ = (288.7, 14.2, 8.2, 6.6). MAD or parameter *D*_max_ can be considered as a proxy of potential or “dark” diversity, which can be used to estimate microbial biodiversity of a DT sites for a human population. For example, at taxonomic genus, the maximal accrual of species richness (Hill numbers for *q* = 0) across individuals is around 289. Similar to the DAR profile, the MAD profile of each DT site decreased with diversity order *q* (Supplementary Table S1 and Figure 1). As shown in Supplementary Tables S2-S5, the *D*_max_ decreased with the taxon level, and for example, the average *D*_max_ at phylum level across *q* = 0–3 is *D*_max_ = (14.7, 4.0, 3.3, 3.1). We test the difference in parameter *D*_max_ between each pair of DT sites by using randomization test, and results were listed in Table 2. At diversity order *q* = 0, two out of 45 or 4.4% comparisons between 10 DT sites exhibited statistically significant differences. These two comparisons with difference were SAL *vs* Stool and SupP *vs* Stool. At diversity order *q* = 1-3, there were 73.3% (33/45), 57.8% (26/45) and 68.9% (31/45) comparisons with significant differences, respectively. Please see Supplementary Table S7 for the detailed results of randomization tests.
iv) *RIP profile*: As shown in Supplementary Table S1, at genus level, the average RIP of the 10 DT sites across diversity order *q* = 0–3 is *RIP* = (19.1, 83.1, 90.8, 93.4), and their standard errors ranged from 1.0 to 3.3. The RIP profiles of each DT site monotonically increased with *q* (Supplementary Table S1 and Figure 1). RIP can characterize the relationship between individual-level diversity and population-level diversity. For example, at diversity order *q* = 0, RIP = 19.1 indicating that an average individual can represent for approximately 19% of population diversity.

**TABLE 1:** Summary of the randomization tests from Supplementary Table S7: the pair-wise comparisons of the DAR parameter (*z*) among the ten DT sites of the human digestive microbiome (at Genus taxon level), each digit of the code (*e.g*., “0,000”) represents the result of randomization test for each diversity order *q* = 0, 1, 2, 3. ‘0 = no significant difference, “1” = significant difference.

Digestive sites	BM	HP	KG	PT	SAL	Stool	SubP	SupP	TD	Th
**BM**	NA	0,000	0,000	0,000	0,000	0,000	0,000	0,000	0,000	0,000
**HP**	0,000	NA	0,000	0,000	0,000	0,000	0,000	0,000	0,000	0,000
**KG**	0,000	0,000	NA	0,000	0,000	0,000	0,000	0,000	0,000	0,000
**FPT**	0,000	0,000	0,000	NA	0,000	0,000	0,000	0,000	0,000	0,000
**SAL**	0,000	0,000	0,000	0,000	NA	0,000	0,000	0,000	0,000	0,000
**Stool**	0,000	0,000	0,000	0,000	0,000	NA	0,000	0,000	0,000	0,000
**SubP**	0,000	0,000	0,000	0,000	0,000	0,000	NA	0,000	0,000	0,000
**SupP**	0,000	0,000	0,000	0,000	0,000	0,000	0,000	NA	0,000	0,000
**TD**	0,000	0,000	0,000	0,000	0,000	0,000	0,000	0,000	NA	0,000
**Th**	0,000	0,000	0,000	0,000	0,000	0,000	0,000	0,000	0,000	NA
*Percentage with Significant Differences (%)	0	0	0	0	0	0	0	0	0	0

* The percentage with significant differences at all diversity orders *q* = 0–3 are the same, i.e., all zeros.

**TABLE 2:** Summary of the randomization tests from Supplementary Table S7: the pair-wise comparisons of the DAR parameter (*D*_max_: potential diversity) among the ten DT sites of the human digestive microbiome (Genus level) for each diversity order *q*. The same coding scheme as in Table 1 was used.

Digestive sites	BM	HP	KG	PT	SAL	Stool	SubP	SupP	TD	Th
**BM**	NA	0,100	0,000	0,101	0,101	0,000	0,101	0,101	0,101	0,101
**HP**	0,100	NA	0,100	0,111	0,111	0,000	0,111	0,111	0,111	0,111
**KG**	0,000	0,100	NA	0,111	0,111	0,000	0,111	0,111	0,111	0,111
**PT**	0,101	0,111	0,111	NA	0,111	0,100	0,111	0,011	0,000	0,000
**SAL**	0,101	0,111	0,111	0,111	NA	1,111	0,111	0,000	0,100	0,011
**Stool**	0,000	0,000	0,000	0,100	1,111	NA	0,111	1,111	0,111	0,100
**SubP**	0,101	0,111	0,111	0,111	0,111	0,111	NA	0,110	0,111	0,111
**SupP**	0,101	0,111	0,111	0,011	0,000	1,111	0,110	NA	0,111	0,011
**TD**	0,101	0,111	0,111	0,000	0,100	0,111	0,111	0,111	NA	0,000
**Th**	0,101	0,111	0,111	0,000	0,011	0,100	0,111	0,011	0,000	NA
***q* = 0 (%)**	0	0	0	0	11.1	22.2	0	11.1	0	0
***q* = 1 (%)**	77.8	88.9	77.8	66.7	77.8	66.7	100	66.7	77.8	55.6
***q* = 2 (%)**	0	66.7	66.7	55.6	66.7	44.4	88.9	77.8	55.6	55.6
***q* = 3 (%)**	66.7	66.7	66.7	66.7	77.8	44.4	88.9	77.8	66.7	66.7

### Intra-DT Diversity Scaling (Across DT Sites) Analysis With Intra-Individual DAR Models

**FIGURE 2:**
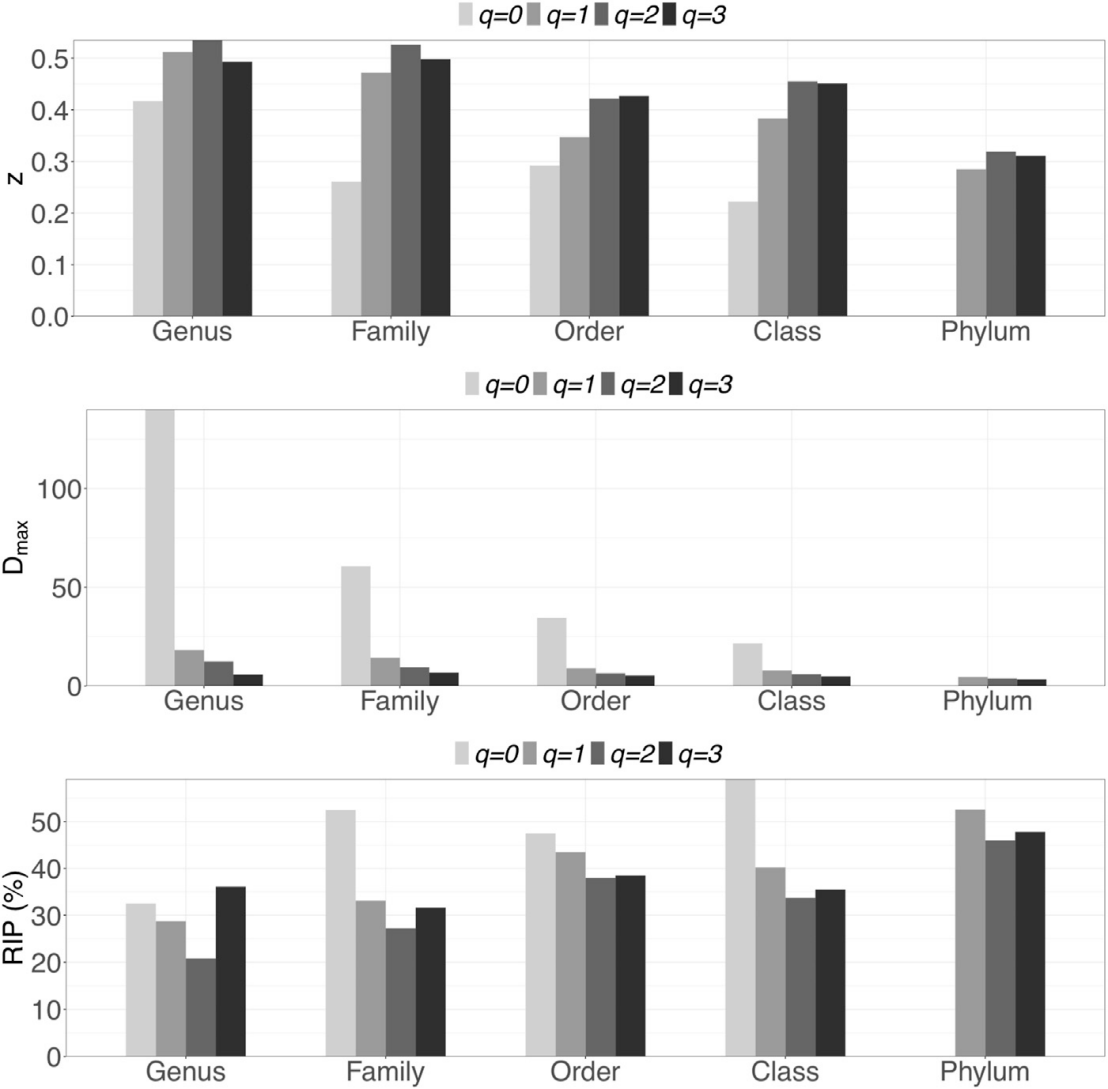
Graphs of three important profiles from intra-individual DAR models for each of the five taxon levels, including DAR profiles (*z-q* patterns), MAD profiles (*D*_max_-*q* patterns), and RIP profiles (*RIP*-*q* patterns). Bar color indicates diversity order.

At each taxon level, we built the PL- and PLEC-DAR models across 10 DT sites to investigate intra-DT distribution pattern. Supplementary Table S6 and Figure 2 list the results of fitting intra-DAR models at all five taxon levels, including the same parameters as Supplementary Table S1. *N* is the number of successful fittings out of 1,000 re-samplings, as explained in the materials and methods section. When *q* = 0, fitting to both intra-DAR models were failed at phylum level. The reason is that different DT sites had the same number of phyla in 890 out of 1,000 re-samplings, which results in the relationship between ln(*D*) and ln(*c*) being equivalent to a line parallel to the *x*-axis, and the estimation of goodness-of-fitting *R* and *p*-value being failed. From Supplementary Table S6, we summarize the following findings:

i) *DAR profile*: At the genus level, the scaling parameter (*z*) across diversity order *q* = 0–3 is *z* = (0.417, 0.512, 0.535, 0.493). DAR profile increased with *q* at *q* = 0–3, but slightly decreased at *q* = 4. Compared with inter-individual DAR profile, there was not much difference in *z*-values between at *q* = 0 and other diversity orders in intra-individual diversity scaling. Similar to the inter-individual diversity scaling, the scaling parameter (*z*) also decreased with the taxon level, but dropped relatively slowly.
ii) *PDO profile*: The trends of PDO profiles over diversity order *q* were contrary to those of DAR profiles.
iii) *MAD profile*: At genus level, *D*_max_ across diversity order *q* = 0–3 is *D*_max_ = (140.0, 18.2, 12.4, 5.8). MAD or parameter *D*_max_ offers estimates for the potential microbial diversity in the whole human DT. For example, at taxonomic genus, the theoretical maximal accrual of species richness (Hill numbers for *q* = 0) across all DT sites is around 140. Similar to the inter-individual MAD profile, *D*_max_ decreased with diversity order *q* and taxon level.
iv) *RIP profile*: At genus level, RIP across diversity order *q* = 0–3 is *RIP* = (32.5, 28.7, 20.8, 36.1). The RIP profiles of each DT sites monotonically increased with *q*. Compared with inter-individual DAR models, the trends of RIP over either diversity order *q* or taxon level were less obvious in intra-individual DAR models.

## Conclusion and Discussion

Understanding the biogeography or the spatial distribution of biodiversity is of critical significance both theoretically and practically. Theoretically, biogeography shows a big picture of community/metacommunity patterns on a larger scale, in our case, the inter-individual microbiome distribution in a human population cohort. A somewhat unique quality of this study is the analysis of intra-individual diversity distribution across the DT sites, using the same DAR tools as used for the inter-individual diversity scaling, which have been a norm of biogeography study in the studies of human microbiomes.

Practically, biogeography of human microbiome is of obvious importance for public health and personified medicine. For instance, understanding the inter-individual heterogeneity is essential for studying and implementing microbiome intervention treatments such as fecal transplantation for treating certain microbiome-associated diseases (Kump et al., 2013; Wei et al., 2015; Weingarden and Vaughn 2017; Ishikawa et al., 2017, 2018; Cohen et al., 2019). The inter-individual heterogeneity can help to identify/explain possible differences in treatment effects. Similarly, studying the intra-individual (along the human DT in this study) diversity scaling is also of important significance, for example, in choosing the optimum location of DT sites for treatment intervention. An additional advantage of our study is that we apply the same DAR approach to investigate both inter-individual and intra-DT (intra-individual) diversity scaling (across individual and across DT sites) of the human DT microbiomes, which makes the integrated analysis of both sources of heterogeneities (inter-individuals and intra-individual) implementable using the same set of parameters (such as diversity scaling rate *z*, potential diversity, and RIP).

In terms of the inter-individual microbial diversity scaling (Supplementary Table S1 and Table 1 and 2), the diversity scaling parameter (*z*) of all 10 DT sites seems invariant with the site—the *z*-values of all sites did not show significant statistical differences. At genus level, the average scaling parameter (rate) (*z*) across 10 sites is 0.294 at diversity order *q* = 0 (species richness), 0.038 at *q* = 1 (Shannon entropy), 0.020 at *q* = 2 (Simpson index), and 0.014 at *q* = 3. The scaling rate of *species richness* (Hill number for *q* = 0) is nearly 10 times larger than those of other diversity orders, *e.g.,* community evenness measured with Shannon entropy. These results suggest that the inter-individual differences in DT microbiome diversity are primarily in the number of microbial genus—species richness, as demonstrated by much higher scaling rate, rather than in general community diversity as demonstrated by Hill numbers for *q* > 1. The inter-individual diversity scaling parameters (*z*) obtained in this study is also consistent with previous study by [Citations: Ma (2018a), Ecology and Evolution, Ma (2018b), Microbial Ecology], in which single gut microbiome diversity scaling was investigated.

Besides inter-individual diversity scaling parameter (*z*), another DAR parameter RIP (ratio of individual to population level diversity) also revealed that the critical differences between individuals lie in species richness (*q* = 0), rather than in community diversity (*q* > 0). The average RIP for species richness (*q* = 0) of 10 DT sites is 19.1% with standard error of 1.0 only, while RIP for general community diversity (*q* = 1–3) ranged from 83.1 to 93.4%. These RIP numbers indicates that an average individual can host approximately 20% of microbial genus owned by a whole population, while the microbial diversity of an individual may exceed 90% the total diversity of a population from which the individual comes from. Furthermore, all 10 DT exhibited very similar inter-individual diversity scaling as described above, which is evidenced by the rather small standard error of the average *z* and RIP across the 10 DT sites. To the best of our knowledge, noprevious studies have addressed the RIP of gut microbiomes.

We also systematically investigated the inter-individual diversity scaling on other four taxa including phylum, class, order, and family (Supplementary Table S2-S5). The scaling patterns are similar to the previously summarized genus-level scaling, but the diversity scaling parameter (*z*) generally decreases with the taxon level. That is, the inter-individual differences in diversity decreases with the higher taxonomic orders. This should be expected since higher diversity order such as phyla and classes are more general (rough) classifications and the similarity in diversity should certainly be higher at more general taxonomic scales. Higher similarity in diversity is equivalent to lower diversity scaling parameter (*z*), *i.e.,* slower scaling rate. To the best of our knowledge, this study should be the first one that studies diversity scaling at taxonomic level beyond species.

In terms of intra-DT diversity scaling patterns (Supplementary Table S6 and Figure 2), at the genus level, the diversity scaling parameter (*z*) at various diversity orders (*q* = 0–3) is actually more similar that inter-individual scaling, which is indicated by the relatively narrow range of *z*-values {0.417 (*q* = 0), 0.512 (*q* = 1), 0.535 (*q* = 2), 0.493 (*q* = 3)}. Similarly, the RIP vector {32.5% (*q* = 0), 28.7% (*q* = 1), 20.8% (*q* = 2), 36.1 (*q* = 3)} also exhibited a range, compared with previous inter-individual. These findings suggest that intra-DT heterogeneity seems to be universally stronger than the inter-individual heterogeneity. This is obviously determined by the human biology, since for intra-DT diversity scaling, we are comparing “apples” and “oranges” (*e.g*., oral site *vs* gut), while inter-individual diversity scaling was comparing the “apples from two apple trees”. Therefore, comparing them may not be that meaningful. The important insight our study revealed is that 1) each DT site hosts approximately 1/5 to 1/3 of the whole DT diversity, and 2) there should be significantly overlaps (similarity) among the DT sites as inferred by (1). This high similarity in intra-DT diversity can be explained by the biological fact that DT is a continuum in which microbial dispersal occurs routinely. On the other hand, the heterogeneity in diversity scaling can be explained that the DT continuum is not homogenous either. In fact, the DT is differentiated as four different niches hosting some functionally different microbial species as revealed in the original study of Segata *et al.* (2012), upon the datasets of which our study is based.

Similar to the inter-individual diversity scaling, we also investigated the intra-DT diversity scaling on other four taxon levels (phylum, class, order, and family) (Supplementary Table S6) beyond genus level. The pattern is similar to previous inter-individual diversity scaling. That is, at higher taxonomic level, the difference becomes smaller or the similarity becomes larger, perhaps like using a telescope to observe remote landscapes. For example, at the taxonomic *class* level, the RIP for *q* = 0 was 59.1%, suggesting that an average individual can host approximately 60% of the microbial classes of a whole population.

A minor limitation of this study is that the species-level DAR analysis was missing given that the original raw sequencing reads reported in Segata *et al.* (2012) were only binned to genus and above taxon levels. Since in many cases, the species level (or 97% similarity level) OTUs are simply a number appended to genus name, and may be of limited biomedical significances. In the meantime, the annotations at higher taxon levels (genus, family, order, class, and phylum) should be rather stable, and the analyses of their diversity scaling can be more useful practically. In fact, to the best of our knowledge, this study should be the first comprehensive analysis of DT microbiomes at and above genus levels.

**FIGURE 3:**
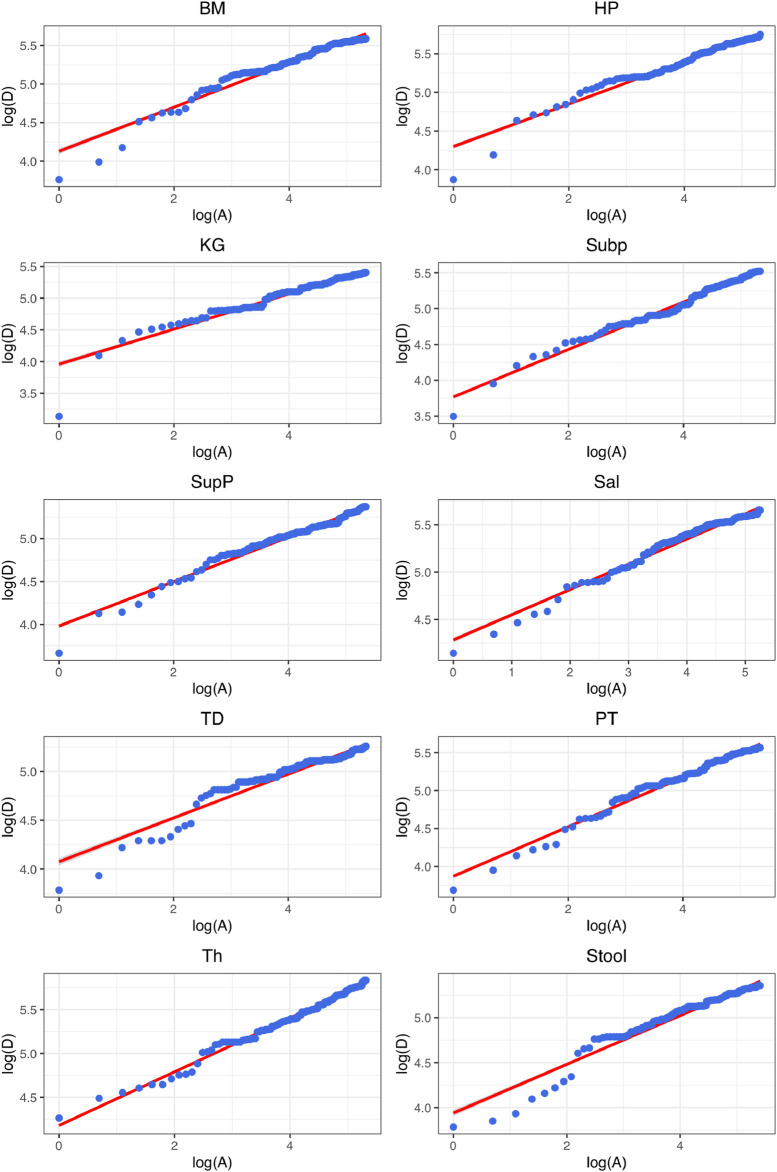
Graphs of fitting the diversity-area relationship (DAR) power-law (PL) model (Eq. 5) for the gut microbiome at each of the 10 DT (digestive tract) site, at the genus taxon level, for diversity order *q* = 0 (*i.e*., species richness).

Finally, yet another minor limitation of this study is that we could not provide in-depth mechanistic interpretation of the observed patterns as revealed by DAR modeling analysis. On the one hand, DAR as an extension to classic SAR inherited both the merits and limitations of SAR. The SAR was discovered more than a century ago (Watson 1835) largely as an empirical relationship, and some scholars called it “collector’s curve”—hinting that when one travels more regions (areas) he or she should be able to collective more species. In the early days of biology, this was indeed the case; botanists and zoologists (collectively known as naturalists, the most famous should be Charles Darwin) were, in the first place, bio-geographers and taxonomists. Arguably one of the most sophisticated mathematical tools they used was the graphing on coordinate papers with pencils, especially on the log-scales. The SAR graphs on coordinate papers would be straight lines (*e.g*., Figure 3 and Eq. 5), and transforming back to mathematical equation turned out to be a power-function (Eq. 3). During the last few decades, many ecologists (*e.g.,* Tjørve and Tjørve 2008; Harte et al., 2009; Sizling et al., 2011; Triantis et al., 2012) have tried to investigate the mechanisms underlying the observed SAR patterns. One of the most influential hypotheses is the self-similarity (scale invariance) hypothesis (*e.g.,* Harte et al., 2009; Sizling et al., 2011), which was also adopted by Ma (2018a) when he extended the classic SAR to general DAR. Interested readers should refer to Harte et al. (2009), Sizling et al. (2011), Ma (2018a) for mechanistic discussion on the SAR and DAR patterns. From a practical perspective, these mechanistic discussions are relatively less relevant, given that SAR has been considered as one of the most important models in conservation biology and biogeography. In our opinion, the DAR, which extends classis SAR from species richness to general diversity metrics, should have equally important applications in microbial biogeography and biogeography in general.

## Data Availability

The original contribution presented in the study are included in the article/Supplementary Material, further inquiries can be directed to the corresponding authors
