# Supplementary material for: Diversity Scaling of Human Digestive Tract (DT) Microbiomes: The Intra-DT and Inter-individual Patterns
Source: Front Genet. 2021 Sep 24;12:724661. doi: 10.3389/fgene.2021.724661 (PMC8497975; doi:10.3389/fgene.2021.724661)
Supplement: Supplementary file 1 [file DataSheet1.PDF]

Online Supplementary Information (OSI) for: Chen *et al.* (2021) Diversity scaling of human digestive tract (DT) microbiomes: the intra-DT and inter-individual patterns. *Frontiers in Genetics*.

The abbreviation list for the 10 digestive tract (DT) sites:

buccal mucosa (BM)
keratinized gingival (KG)
hard palate (HP)
throat (Th)
palatine tonsils (PT)
tongue dorsum (TD)
saliva (Sal)
supraginval (SupP)
subgingival plaques (SubP)
stool (Stool).

The five taxon-level for DAR analysis:

genus
phylum
class
order
family

List of Supplementary Tables:

Table S1. The DAR (Diversity-Area Relationship) models for each of the 10 DT (digestive tract) microbiome sites at the ***genus*** taxon-level

Table S2. Fitting the DAR (Diversity-Area Relationship) models for each of the 10 digestive microbiome sites at the ***phylum*** taxon-level

Table S3. Fitting the DAR (Diversity-Area Relationship) models for each of the 10 digestive microbiome sites at the ***class*** taxon-level

Table S4. Fitting the DAR (Diversity-Area Relationship) models for each of the 10 digestive microbiome sites at the ***order*** taxon-level

Table S5. Fitting the DAR (Diversity-Area Relationship) models for each of the 10 digestive microbiome sites at the ***family*** taxon-level

Table S6. The intra-DAR (intra-individual Diversity-Area Relationship) models

Table S7. The permutation test for the differences in the DAR parameters between the digestive sites via 1000 times of re-sampling, at the species taxon level, for each diversity order (q=0-3).

Table S1. The DAR (Diversity-Area Relationship) models for each of the 10 DT (digestive tract) microbiome sites at the ***genus*** taxon-level.

Site	Diversity Order	Power Law (PL)						PL with Exponential Cutoff (PLEC)								RIP
		z	*c*	*R*	*p*-value	*g*	**N*	*z*	*d*	*c*	*R*	*p*-value	**N*	A_{max}	D_{max}	
BM	*q=0*	0.289	59.145	0.984	0.000	0.778	100	0.349	-0.001	51.009	0.992	0.000	100	323	270.1	21.9
	q=1	0.033	6.475	0.538	0.044	0.976	88	0.058	0.000	6.086	0.675	0.003	99	130	7.6	85.2
	q=2	0.009	3.105	0.526	0.029	0.993	92	0.011	0.000	3.089	0.670	0.006	95	396	3.3	94.1
	q=3	0.003	2.482	0.521	0.019	0.997	94	0.000	0.000	2.502	0.669	0.007	99	2	2.5	99.3
HP	*q=0*	0.302	64.586	0.99	0.000	0.767	100	0.343	-0.001	58.323	0.995	0.000	100	453	337.7	19.1
	q=1	0.018	9.564	0.445	0.082	0.987	83	0.034	0.000	9.198	0.613	0.007	95	117	10.5	91.1
	q=2	-0.015	4.545	0.474	0.055	1.009	84	-0.025	0.000	4.655	0.620	0.010	95	141	4.2	100**
	q=3	-0.021	3.449	0.489	0.054	1.014	91	-0.038	0.000	3.600	0.629	0.013	98	121	3.1	100**
KG	*q=0*	0.345	36.416	0.983	0.000	0.73	100	0.407	-0.001	31.187	0.990	0.000	100	364	229.3	15.9
	q=1	0.049	5.675	0.56	0.037	0.964	93	0.096	-0.001	5.058	0.692	0.003	98	115	7.2	78.8
	q=2	0.011	3.254	0.516	0.028	0.991	88	0.027	0.000	3.124	0.660	0.006	96	93	3.4	95.7
	q=3	0.001	2.649	0.5	0.106	0.998	80	0.007	0.000	2.612	0.654	0.014	97	67	2.7	98.1
SubP	*q=0*	0.280	56.997	0.991	0.000	0.786	100	0.303	0.000	53.839	0.995	0.000	100	724	292.5	19.5
	q=1	0.038	17.567	0.709	0.008	0.973	98	0.075	-0.001	16.023	0.817	0.000	100	113	21.2	82.9
	q=2	0.046	11.635	0.686	0.018	0.967	96	0.092	-0.001	10.381	0.810	0.008	99	112	14.6	79.7
	q=3	0.054	9.621	0.68	0.015	0.962	94	0.104	-0.001	8.491	0.801	0.000	100	114	12.5	77.0
SupP	*q=0*	0.281	48.327	0.99	0.000	0.785	100	0.288	0.000	47.418	0.993	0.000	100	2164	325.4	14.9
	q=1	0.038	15.364	0.598	0.045	0.973	93	0.072	-0.001	14.098	0.736	0.000	100	118	18.5	83.0
	q=2	0.043	10.454	0.583	0.024	0.969	91	0.080	-0.001	9.535	0.720	0.001	99	123	12.9	81.0
	q=3	0.046	8.793	0.567	0.031	0.967	90	0.083	-0.001	8.029	0.711	0.004	99	128	11	79.9
SAL	*q=0*	0.293	61.992	0.99	0.000	0.775	100	0.324	-0.001	57.512	0.994	0.000	100	536	319.3	19.4
	q=1	0.027	16.827	0.604	0.019	0.981	94	0.055	-0.001	15.721	0.745	0.007	97	101	19.2	87.6
	q=2	0.023	10.486	0.534	0.033	0.983	90	0.046	0.000	9.924	0.685	0.003	99	105	11.8	88.9
	q=3	0.022	8.628	0.519	0.034	0.984	91	0.043	0.000	8.207	0.669	0.000	100	107	9.6	89.9
TD	*q=0*	0.274	44.835	0.988	0.000	0.791	100	0.291	0.000	42.905	0.992	0.000	100	945	235.9	19.0
	q=1	0.036	12.833	0.595	0.032	0.975	92	0.071	-0.001	11.752	0.732	0.000	100	115	15.3	83.9
	q=2	0.036	8.593	0.507	0.044	0.974	87	0.070	-0.001	7.893	0.661	0.003	99	117	10.3	83.4
	q=3	0.031	7.199	0.479	0.038	0.978	89	0.062	-0.001	6.659	0.636	0.013	98	113	8.4	85.7
PT	*q=0*	0.291	56.599	0.986	0.000	0.776	100	0.329	-0.001	51.522	0.991	0.000	100	492	284.6	19.9
	q=1	0.045	12.897	0.542	0.025	0.968	90	0.087	-0.001	11.612	0.678	0.000	100	117	16.1	80.1
	q=2	0.027	7.546	0.456	0.050	0.980	86	0.062	-0.001	6.910	0.583	0.019	95	100	8.7	86.7
	q=3	0.012	6.007	0.467	0.047	0.990	89	0.037	0.000	5.641	0.588	0.021	96	82	6.4	93.9
Th	*q=0*	0.330	60.947	0.989	0.000	0.743	100	0.369	-0.001	55.313	0.993	0.000	100	513	382	16.0
	q=1	0.042	13.585	0.551	0.064	0.970	86	0.081	-0.001	12.342	0.701	0.005	98	113	16.7	81.4
	q=2	0.017	7.815	0.477	0.062	0.987	86	0.043	0.000	7.330	0.632	0.015	94	89	8.5	91.9
	q=3	0.003	6.135	0.484	0.035	0.997	91	0.020	0.000	5.894	0.639	0.006	99	65	6.3	97.4
Stool	*q=0*	0.259	54.055	0.976	0.000	0.803	100	0.323	-0.001	45.925	0.986	0.000	100	303	210.5	25.7
	q=1	0.051	7.822	0.51	0.056	0.963	86	0.107	-0.001	6.780	0.691	0.004	99	115	10.1	77.5
	q=2	0.002	4.116	0.461	0.066	0.997	88	0.028	0.000	3.854	0.636	0.018	97	65	4.2	98.0
	q=3	-0.010	3.245	0.46	0.027	1.006	84	0.002	0.000	3.146	0.629	0.012	97	9	3.2	100**
q=0	**Mean**	0.294	54.390	0.987	0.000	0.773	100	0.333	-0.001	49.495	0.992	0.000	100	682	288.7	19.1
	**Std Error**	0.008	2.769	0.001	0.000	0.007	0	0.012	0.000	2.576	0.001	0.000	0	176	17.1	1.0
q=1	**Mean**	0.038	11.861	0.565	0.041	0.973	90	0.074	-0.001	10.867	0.708	0.003	99	115	14.2	83.1
	**Std Error**	0.003	1.347	0.022	0.007	0.002	1	0.007	0.000	1.248	0.017	0.001	1	2	1.6	1.3
q=2	**Mean**	0.020	7.155	0.522	0.041	0.985	89	0.043	0.000	6.670	0.668	0.009	97	134	8.2	90.8
	**Std Error**	0.006	1.013	0.022	0.005	0.004	1	0.011	0.000	0.894	0.020	0.002	1	30	1.3	2.8
q=3	**Mean**	0.014	5.821	0.517	0.041	0.989	89	0.032	0.000	5.478	0.663	0.009	98	81	6.6	93.4

	**Std Error**	0.008	0.861	0.021	0.008	0.005	1	0.014	0.000	0.750	0.018	0.002	0	14	1.2	3.3

* The model parameters were computed from 100 times of repeated DAR modeling based on 100 times of re-sampling; *N* is the number of successful DAR model-fitting out of 100 times of re-sampling.

** The theoretical maximum of RIP is 100%, any RIP exceeding 100% may be caused by estimation errors.

Table S2. Fitting the DAR (Diversity-Area Relationship) models for each of the 10 digestive microbiome sites at the ***phylum*** taxon-level

Digestive Site	Diversity Order & Statistics		Power Law (PL)						PL with Exponential Cutoff (PLEC)							
			z	ln(*c*)	*R*	*p*-value	*g*	*N**	*z*	*d*	ln(*c*)	*R*	*p*-value	*N**	A_{max}	D_{max}
BM	*q=0*	Mean	0.100	2.168	0.895	0.000	0.928	100	0.106	0.000	2.153	0.920	0.000	100	947	16.0
		Std. Err.	0.022	0.102	0.050	0.000	0.016		0.051	0.001	0.146	0.044	0.000			
		Min	0.046	1.865	0.743	0.000	0.882		-0.006	-0.002	1.784	0.750	0.000			
		Max	0.161	2.401	0.976	0.000	0.968		0.235	0.001	2.473	0.983	0.000			
	q=1	Mean	0.014	0.989	0.498	0.052	0.990	88	0.022	0.000	0.967	0.648	0.019	97	141	2.9
		Std. Err.	0.034	0.171	0.284	0.160	0.024		0.062	0.001	0.221	0.232	0.115			
		Min	-0.079	0.583	0.011	0.000	0.933		-0.110	-0.002	0.435	0.011	0.000			
		Max	0.094	1.468	0.976	0.871	1.053		0.169	0.001	1.492	0.978	0.987			
	q=2	Mean	0.004	0.682	0.483	0.052	0.997	88	0.006	0.000	0.679	0.635	0.001	100	232	2.0
		Std. Err.	0.038	0.189	0.286	0.174	0.026		0.069	0.001	0.246	0.232	0.005			
		Min	-0.105	0.299	0.007	0.000	0.941		-0.160	-0.002	0.166	0.175	0.000			
		Max	0.082	1.244	0.969	0.925	1.070		0.145	0.002	1.316	0.979	0.041			
	q=3	Mean	0.001	0.567	0.484	0.049	0.999	89	-0.001	0.000	0.572	0.636	0.004	98	30	1.8
		Std. Err.	0.035	0.175	0.284	0.162	0.024		0.065	0.001	0.233	0.229	0.024			
		Min	-0.104	0.228	0.011	0.000	0.951		-0.166	-0.002	0.117	0.130	0.000			
		Max	0.069	1.103	0.959	0.871	1.070		0.122	0.002	1.203	0.977	0.174			
HP	*q=0*	Mean	0.119	2.158	0.927	0.000	0.914	100	0.145	0.000	2.095	0.949	0.000	100	310	16.1
		Std. Err.	0.023	0.119	0.046	0.000	0.017		0.048	0.001	0.142	0.034	0.000			
		Min	0.063	1.826	0.721	0.000	0.868		0.011	-0.003	1.730	0.790	0.000			
		Max	0.179	2.433	0.977	0.000	0.955		0.261	0.002	2.490	0.989	0.000			
	q=1	Mean	0.001	1.171	0.495	0.054	0.999	91	0.007	0.000	1.156	0.646	0.007	98	66	3.3
		Std. Err.	0.030	0.150	0.255	0.190	0.021		0.052	0.001	0.184	0.215	0.051			
		Min	-0.065	0.796	0.007	0.000	0.944		-0.118	-0.002	0.698	0.084	0.000			
		Max	0.079	1.500	0.928	0.924	1.044		0.143	0.001	1.592	0.955	0.490			
	q=2	Mean	-0.008	0.881	0.507	0.044	1.005	90	-0.009	0.000	0.884	0.665	0.017	97	546	2.3
		Std. Err.	0.038	0.193	0.267	0.167	0.026		0.066	0.001	0.235	0.209	0.108			
		Min	-0.096	0.422	0.000	0.000	0.938		-0.179	-0.002	0.343	0.039	0.000			
		Max	0.086	1.320	0.944	0.997	1.065		0.155	0.002	1.486	0.953	0.860			
	q=3	Mean	-0.011	0.750	0.508	0.042	1.007	86	-0.016	0.000	0.762	0.669	0.017	97	189	2.0
		Std. Err.	0.038	0.188	0.271	0.129	0.026		0.065	0.001	0.232	0.208	0.101			
		Min	-0.101	0.319	0.024	0.000	0.945		-0.190	-0.002	0.256	0.059	0.000			
		Max	0.077	1.190	0.945	0.731	1.068		0.140	0.002	1.382	0.951	0.701			
KG	*q=0*	Mean	0.107	2.009	0.898	0.000	0.923	100	0.135	-0.001	1.938	0.928	0.000	100	264	12.9
		Std. Err.	0.026	0.130	0.064	0.000	0.019		0.046	0.001	0.140	0.044	0.000			
		Min	0.039	1.743	0.520	0.000	0.882		0.000	-0.002	1.597	0.724	0.000			
		Max	0.161	2.386	0.977	0.000	0.973		0.264	0.002	2.245	0.985	0.000			
	q=1	Mean	0.015	0.961	0.456	0.051	0.989	88	0.028	0.000	0.930	0.614	0.018	95	122	2.8
		Std. Err.	0.032	0.154	0.261	0.170	0.022		0.065	0.001	0.217	0.234	0.097			
		Min	-0.060	0.468	0.004	0.000	0.918		-0.138	-0.002	0.224	0.052	0.000			
		Max	0.114	1.318	0.939	0.950	1.040		0.212	0.002	1.440	0.960	0.757			
	q=2	Mean	0.007	0.742	0.456	0.060	0.995	87	0.012	0.000	0.732	0.608	0.006	94	155	2.2
		Std. Err.	0.037	0.181	0.263	0.176	0.026		0.072	0.001	0.245	0.235	0.025			
		Min	-0.095	0.238	0.017	0.000	0.922		-0.161	-0.003	0.051	0.134	0.000			
		Max	0.108	1.239	0.921	0.805	1.064		0.183	0.002	1.386	0.966	0.154			
	q=3	Mean	0.003	0.650	0.463	0.064	0.998	85	0.003	0.000	0.650	0.612	0.011	96	0	NA
		Std. Err.	0.037	0.182	0.263	0.195	0.026		0.072	0.001	0.246	0.234	0.066			
		Min	-0.107	0.177	0.002	0.000	0.930		-0.174	-0.002	0.026	0.069	0.000			
		Max	0.097	1.187	0.921	0.972	1.072		0.158	0.002	1.353	0.969	0.615			
PT	*q=0*	Mean	0.100	2.231	0.908	0.000	0.928	100	0.095	0.000	2.243	0.930	0.000	100	0	NA
		Std. Err.	0.024	0.114	0.061	0.000	0.018		0.047	0.001	0.135	0.050	0.000			
		Min	0.026	1.903	0.623	0.000	0.878		-0.020	-0.002	1.889	0.627	0.000			
		Max	0.166	2.530	0.974	0.000	0.982		0.224	0.002	2.581	0.979	0.000			
	q=1	Mean	0.019	1.368	0.511	0.028	0.987	88	0.040	0.000	1.316	0.669	0.006	99	107	4.3
		Std. Err.	0.030	0.149	0.262	0.086	0.021		0.061	0.001	0.209	0.197	0.053			
		Min	-0.034	0.995	0.048	0.000	0.930		-0.067	-0.003	0.790	0.079	0.000			
		Max	0.098	1.619	0.920	0.488	1.023		0.212	0.001	1.689	0.931	0.527			
	q=2	Mean	0.015	1.133	0.500	0.031	0.989	86	0.033	0.000	1.089	0.664	0.002	98	106	3.4
		Std. Err.	0.041	0.204	0.258	0.109	0.029		0.080	0.001	0.276	0.191	0.015			
		Min	-0.062	0.658	0.019	0.000	0.917		-0.132	-0.003	0.444	0.140	0.000			
		Max	0.115	1.515	0.943	0.783	1.042		0.216	0.002	1.579	0.950	0.131			
	q=3	Mean	0.010	1.018	0.497	0.051	0.993	89	0.022	0.000	0.987	0.659	0.007	98	100	2.9
		Std. Err.	0.046	0.227	0.256	0.179	0.032		0.087	0.001	0.299	0.195	0.050			
		Min	-0.080	0.533	0.006	0.000	0.920		-0.168	-0.003	0.324	0.084	0.000			
		Max	0.111	1.469	0.954	0.932	1.054		0.213	0.002	1.525	0.958	0.477			
SAL	*q=0*	Mean	0.109	2.242	0.912	0.000	0.921	100	0.086	0.000	2.297	0.941	0.000	100	0	NA
		Std. Err.	0.021	0.100	0.050	0.000	0.015		0.049	0.001	0.110	0.035	0.000			
		Min	0.048	2.044	0.719	0.000	0.887		-0.020	-0.002	2.031	0.794	0.000			
		Max	0.154	2.503	0.971	0.000	0.966		0.196	0.003	2.569	0.984	0.000			
		Mean	0.013	1.500	0.543	0.051	0.991	89	0.025	0.000	1.470	0.691	0.003	98	105	4.8

	q=1	Std. Err.	0.020	0.096	0.273	0.172	0.014		0.036	0.000	0.126	0.200	0.019			
		Min	-0.034	1.251	0.005	0.000	0.956		-0.054	-0.002	1.069	0.137	0.000			
		Max	0.063	1.728	0.945	0.940	1.023		0.149	0.001	1.746	0.964	0.166			
	q=2	Mean	0.010	1.277	0.499	0.070	0.993	85	0.021	0.000	1.253	0.669	0.008	96	104	3.8
		Std. Err.	0.026	0.127	0.270	0.195	0.018		0.049	0.001	0.170	0.215	0.040			
		Min	-0.048	0.937	0.001	0.000	0.941		-0.080	-0.002	0.745	0.110	0.000			
		Max	0.083	1.558	0.937	0.991	1.033		0.172	0.001	1.576	0.950	0.318			
	q=3	Mean	0.009	1.171	0.482	0.044	0.994	84	0.018	0.000	1.149	0.660	0.017	96	100	3.4
		Std. Err.	0.030	0.147	0.270	0.120	0.021		0.057	0.001	0.194	0.226	0.092			
		Min	-0.054	0.772	0.035	0.000	0.934		-0.107	-0.002	0.605	0.074	0.000			
		Max	0.093	1.474	0.943	0.629	1.037		0.178	0.002	1.496	0.948	0.595			
Stool	*q=0*	Mean	0.133	2.036	0.906	0.000	0.903	100	0.206	-0.001	1.850	0.950	0.000	100	169	14.9
		Std. Err.	0.030	0.146	0.052	0.000	0.023		0.057	0.001	0.196	0.032	0.000			
		Min	0.052	1.623	0.759	0.000	0.838		0.074	-0.004	1.339	0.818	0.000			
		Max	0.217	2.447	0.976	0.000	0.963		0.390	0.000	2.390	0.991	0.000			
	q=1	Mean	0.016	0.803	0.541	0.054	0.989	91	0.032	0.000	0.763	0.705	0.010	99	121	2.4
		Std. Err.	0.025	0.128	0.271	0.187	0.018		0.044	0.001	0.154	0.196	0.098			
		Min	-0.051	0.485	0.002	0.000	0.949		-0.068	-0.002	0.398	0.012	0.000			
		Max	0.072	1.141	0.923	0.980	1.035		0.143	0.001	1.141	0.961	0.984			
	q=2	Mean	0.012	0.651	0.484	0.028	0.992	95	0.027	0.000	0.611	0.645	0.000	100	106	2.0
		Std. Err.	0.026	0.135	0.251	0.134	0.019		0.045	0.001	0.164	0.210	0.002			
		Min	-0.046	0.312	0.002	0.000	0.942		-0.067	-0.002	0.174	0.189	0.000			
		Max	0.082	0.951	0.950	0.974	1.031		0.139	0.001	1.004	0.974	0.018			
	q=3	Mean	0.011	0.597	0.464	0.047	0.992	89	0.027	0.000	0.556	0.630	0.010	97	101	1.9
		Std. Err.	0.029	0.149	0.265	0.159	0.020		0.048	0.001	0.174	0.224	0.067			
		Min	-0.053	0.224	0.001	0.000	0.939		-0.072	-0.002	0.095	0.065	0.000			
		Max	0.086	0.930	0.954	0.983	1.036		0.143	0.001	0.977	0.974	0.625			
SubP	*q=0*	Mean	0.094	2.250	0.901	0.000	0.933	100	0.084	0.000	2.274	0.924	0.000	100	0	NA
		Std. Err.	0.018	0.086	0.052	0.000	0.013		0.046	0.001	0.121	0.046	0.000			
		Min	0.053	2.014	0.727	0.000	0.901		-0.016	-0.002	1.976	0.727	0.000			
		Max	0.136	2.455	0.977	0.000	0.962		0.207	0.001	2.547	0.977	0.000			
	q=1	Mean	0.016	1.660	0.571	0.026	0.989	91	0.030	0.000	1.624	0.710	0.002	99	118	5.7
		Std. Err.	0.016	0.076	0.257	0.117	0.011		0.033	0.000	0.115	0.206	0.020			
		Min	-0.020	1.465	0.007	0.000	0.960		-0.033	-0.002	1.265	0.125	0.000			
		Max	0.057	1.842	0.975	0.918	1.014		0.144	0.001	1.869	0.977	0.203			
	q=2	Mean	0.021	1.547	0.618	0.001	0.985	99	0.042	0.000	1.495	0.743	0.000	100	112	5.2
		Std. Err.	0.017	0.080	0.207	0.007	0.012		0.038	0.000	0.128	0.173	0.000			
		Min	-0.013	1.316	0.128	0.000	0.948		-0.021	-0.002	1.021	0.278	0.000			
		Max	0.073	1.717	0.963	0.066	1.009		0.194	0.000	1.735	0.981	0.000			
	q=3	Mean	0.026	1.492	0.628	0.011	0.981	96	0.053	0.000	1.427	0.752	0.000	100	111	5.1
		Std. Err.	0.019	0.090	0.211	0.066	0.013		0.042	0.000	0.142	0.173	0.001			
		Min	-0.008	1.241	0.036	0.000	0.940		-0.014	-0.003	0.895	0.205	0.000			
		Max	0.084	1.662	0.949	0.604	1.005		0.223	0.000	1.672	0.986	0.012			
SupP	*q=0*	Mean	0.049	2.332	0.764	0.000	0.966	100	0.096	-0.001	2.213	0.884	0.000	100	114	13.1
		Std. Err.	0.018	0.087	0.121	0.000	0.013		0.035	0.000	0.121	0.076	0.000			
		Min	0.003	2.115	0.316	0.000	0.933		0.010	-0.002	1.923	0.469	0.000			
		Max	0.093	2.550	0.939	0.000	0.998		0.175	0.000	2.533	0.962	0.000			
	q=1	Mean	0.016	1.588	0.560	0.025	0.989	92	0.030	0.000	1.552	0.685	0.002	98	120	5.3
		Std. Err.	0.020	0.098	0.258	0.103	0.014		0.037	0.000	0.133	0.218	0.016			
		Min	-0.029	1.363	0.033	0.000	0.956		-0.042	-0.002	1.148	0.134	0.000			
		Max	0.062	1.821	0.952	0.632	1.020		0.149	0.000	1.807	0.965	0.154			
	q=2	Mean	0.021	1.474	0.541	0.024	0.985	94	0.040	0.000	1.427	0.655	0.009	96	121	4.9
		Std. Err.	0.025	0.126	0.253	0.115	0.018		0.046	0.001	0.169	0.236	0.047			
		Min	-0.034	1.157	0.000	0.000	0.939		-0.044	-0.002	0.926	0.104	0.000			
		Max	0.086	1.752	0.961	1.000	1.023		0.184	0.001	1.731	0.974	0.322			
	q=3	Mean	0.026	1.415	0.532	0.028	0.982	92	0.048	0.000	1.360	0.650	0.013	96	122	4.7
		Std. Err.	0.030	0.148	0.254	0.127	0.021		0.053	0.001	0.194	0.237	0.073			
		Min	-0.038	1.011	0.006	0.000	0.924		-0.048	-0.002	0.797	0.078	0.000			
		Max	0.105	1.736	0.958	0.932	1.026		0.202	0.001	1.708	0.978	0.534			
TD	*q=0*	Mean	0.099	2.171	0.911	0.000	0.929	100	0.109	0.000	2.146	0.931	0.000	100	606	15.4
		Std. Err.	0.024	0.113	0.054	0.000	0.018		0.043	0.001	0.133	0.048	0.000			
		Min	0.039	1.814	0.638	0.000	0.871		0.010	-0.001	1.727	0.654	0.000			
		Max	0.175	2.473	0.978	0.000	0.973		0.216	0.001	2.451	0.982	0.000			
	q=1	Mean	0.009	1.481	0.529	0.024	0.994	93	0.021	0.000	1.452	0.658	0.017	96	100	4.6
		Std. Err.	0.023	0.113	0.248	0.108	0.016		0.042	0.000	0.151	0.216	0.108			
		Min	-0.046	1.143	0.025	0.000	0.946		-0.064	-0.001	0.960	0.030	0.000			
		Max	0.076	1.738	0.933	0.715	1.032		0.149	0.001	1.780	0.971	0.910			
	q=2	Mean	0.006	1.304	0.523	0.043	0.996	88	0.015	0.000	1.281	0.675	0.004	99	90	3.8
		Std. Err.	0.034	0.169	0.261	0.147	0.024		0.060	0.001	0.217	0.193	0.041			
		Min	-0.072	0.808	0.013	0.000	0.926		-0.100	-0.002	0.595	0.092	0.000			
		Max	0.103	1.659	0.938	0.847	1.048		0.189	0.001	1.706	0.971	0.414			
	q=3	Mean	0.000	1.200	0.521	0.042	1.000	91	0.005	0.000	1.188	0.688	0.000	100	56	3.3
		Std. Err.	0.040	0.199	0.275	0.157	0.028		0.069	0.001	0.250	0.182	0.004			
		Min	-0.089	0.652	0.002	0.000	0.923		-0.130	-0.002	0.450	0.177	0.000			
		Max	0.108	1.610	0.944	0.972	1.060		0.188	0.002	1.679	0.968	0.036			

Th	*q=0*	Mean	0.168	2.078	0.948	0.000	0.876	100	0.135	0.001	2.159	0.966	0.000	100	0	NA
		Std. Err.	0.026	0.127	0.036	0.000	0.020		0.059	0.001	0.151	0.017	0.000			
		Min	0.097	1.843	0.789	0.000	0.835		-0.026	-0.001	1.851	0.904	0.000			
		Max	0.220	2.378	0.987	0.000	0.931		0.230	0.004	2.514	0.990	0.000			
	q=1	Mean	0.007	1.392	0.467	0.047	0.995	88	0.021	0.000	1.359	0.606	0.014	97	84	4.2
		Std. Err.	0.024	0.118	0.253	0.161	0.017		0.042	0.000	0.151	0.219	0.096			
		Min	-0.050	1.071	0.011	0.000	0.949		-0.060	-0.001	0.964	0.032	0.000			
		Max	0.071	1.688	0.936	0.872	1.034		0.128	0.001	1.615	0.954	0.905			
	q=2	Mean	0.000	1.160	0.471	0.055	1.000	85	0.009	0.000	1.136	0.613	0.014	96	54	3.2
		Std. Err.	0.035	0.174	0.259	0.172	0.024		0.062	0.001	0.217	0.216	0.076			
		Min	-0.094	0.740	0.011	0.000	0.941		-0.108	-0.003	0.599	0.069	0.000			
		Max	0.083	1.643	0.896	0.872	1.063		0.166	0.001	1.540	0.969	0.621			
	q=3	Mean	-0.006	1.035	0.476	0.061	1.004	84	-0.002	0.000	1.025	0.616	0.019	95	0	NA
		Std. Err.	0.039	0.193	0.268	0.183	0.027		0.068	0.001	0.240	0.229	0.100			
		Min	-0.117	0.609	0.006	0.000	0.944		-0.135	-0.003	0.451	0.057	0.000			
		Max	0.078	1.608	0.905	0.929	1.078		0.178	0.002	1.520	0.971	0.721			

* The model parameters were computed from 100 times of repeated DAR modeling from 100 times of re-sampling; *N* is the number of successful DAR model-fitting out of 100 times of re-sampling.

Table S3. Fitting the DAR (Diversity-Area Relationship) models for each of the 10 digestive microbiome sites at the ***class*** taxon-level

**Digestive Site**	**Diversity Order & Statistics**		**Power Law (PL)**						**PL with Exponential Cutoff (PLEC)**							
			z	**ln(*c*)**	***R***	***p*-value**	***g***	****N***	***z***	***d***	**ln(*c*)**	***R***	***p*-value**	****N***	A_{max}	D_{max}
BM	*q=0*	Mean	0.099	2.727	0.929	0.000	0.929	100	0.118	0.000	2.680	0.948	0.000	100	346	25.8
		Std. Err.	0.019	0.093	0.047	0.000	0.014		0.041	0.001	0.123	0.037	0.000			
		Min	0.042	2.494	0.698	0.000	0.894		0.029	-0.002	2.371	0.723	0.000			
		Max	0.145	3.039	0.985	0.000	0.971		0.238	0.001	2.993	0.989	0.000			
	q=1	Mean	0.012	1.315	0.482	0.055	0.991	90	0.025	0.000	1.283	0.624	0.007	96	108	3.9
		Std. Err.	0.044	0.220	0.267	0.188	0.031		0.080	0.001	0.285	0.229	0.036			
		Min	-0.103	0.823	0.004	0.000	0.918		-0.149	-0.002	0.684	0.103	0.000			
		Max	0.113	1.903	0.962	0.952	1.069		0.205	0.002	1.987	0.972	0.334			
	q=2	Mean	-0.002	0.867	0.476	0.052	1.001	87	-0.004	0.000	0.870	0.616	0.006	96	128	2.4
		Std. Err.	0.048	0.239	0.262	0.169	0.033		0.086	0.001	0.310	0.229	0.032			
		Min	-0.146	0.352	0.020	0.000	0.925		-0.225	-0.002	0.277	0.117	0.000			
		Max	0.104	1.616	0.953	0.779	1.096		0.174	0.002	1.785	0.976	0.246			
	q=3	Mean	-0.006	0.707	0.474	0.070	1.004	86	-0.011	0.000	0.721	0.616	0.009	97	111	2.0
		Std. Err.	0.043	0.214	0.265	0.212	0.030		0.079	0.001	0.283	0.230	0.053			
		Min	-0.149	0.261	0.002	0.000	0.939		-0.237	-0.002	0.203	0.091	0.000			
		Max	0.086	1.448	0.956	0.980	1.098		0.143	0.002	1.630	0.979	0.427			
HP	*q=0*	Mean	0.130	2.707	0.949	0.000	0.906	100	0.145	0.000	2.669	0.960	0.000	100	521	30.9
		Std. Err.	0.021	0.097	0.030	0.000	0.016		0.043	0.001	0.129	0.025	0.000			
		Min	0.070	2.496	0.832	0.000	0.868		0.039	-0.002	2.397	0.859	0.000			
		Max	0.179	2.973	0.988	0.000	0.950		0.242	0.001	3.019	0.991	0.000			
	q=1	Mean	0.011	1.540	0.512	0.039	0.992	92	0.026	0.000	1.504	0.657	0.000	100	96	4.9
		Std. Err.	0.037	0.182	0.253	0.150	0.026		0.070	0.001	0.242	0.190	0.005			
		Min	-0.060	1.158	0.009	0.000	0.932		-0.120	-0.002	0.858	0.173	0.000			
		Max	0.094	1.912	0.928	0.894	1.041		0.216	0.002	1.992	0.962	0.047			
	q=2	Mean	0.001	1.148	0.510	0.056	0.999	88	0.006	0.000	1.136	0.662	0.002	98	65	3.2
		Std. Err.	0.050	0.249	0.271	0.177	0.035		0.090	0.001	0.315	0.189	0.013			
		Min	-0.101	0.661	0.005	0.000	0.923		-0.203	-0.003	0.345	0.146	0.000			
		Max	0.107	1.672	0.918	0.942	1.067		0.236	0.002	1.838	0.949	0.115			
	q=3	Mean	-0.005	0.972	0.513	0.043	1.003	90	-0.006	0.000	0.975	0.662	0.003	98	312	2.6
		Std. Err.	0.049	0.244	0.267	0.159	0.034		0.088	0.001	0.311	0.190	0.030			
		Min	-0.109	0.506	0.002	0.000	0.932		-0.224	-0.003	0.239	0.110	0.000			
		Max	0.095	1.507	0.918	0.978	1.073		0.211	0.002	1.706	0.946	0.294			
KG	*q=0*	Mean	0.106	2.588	0.930	0.000	0.923	100	0.136	-0.001	2.514	0.951	0.000	100	255	22.9
		Std. Err.	0.020	0.102	0.032	0.000	0.015		0.044	0.001	0.139	0.029	0.000			
		Min	0.060	2.369	0.844	0.000	0.890		0.041	-0.002	2.162	0.845	0.000			
		Max	0.151	2.837	0.976	0.000	0.958		0.250	0.001	2.825	0.988	0.000			
	q=1	Mean	0.020	1.301	0.477	0.059	0.985	87	0.040	0.000	1.252	0.641	0.009	95	115	4.1
		Std. Err.	0.046	0.234	0.255	0.180	0.033		0.090	0.001	0.297	0.226	0.050			
		Min	-0.081	0.767	0.002	0.000	0.905		-0.195	-0.003	0.537	0.088	0.000			
		Max	0.131	1.858	0.947	0.978	1.055		0.267	0.003	1.829	0.972	0.453			
	q=2	Mean	0.002	0.959	0.471	0.038	0.998	92	0.008	0.000	0.944	0.633	0.013	98	78	2.6
		Std. Err.	0.052	0.265	0.268	0.151	0.036		0.093	0.001	0.323	0.230	0.099			
		Min	-0.127	0.363	0.008	0.000	0.913		-0.228	-0.003	0.159	0.025	0.000			
		Max	0.121	1.657	0.945	0.914	1.084		0.258	0.003	1.597	0.964	0.940			
	q=3	Mean	-0.004	0.827	0.473	0.039	1.002	85	-0.005	0.000	0.828	0.637	0.006	96	1085	2.2
		Std. Err.	0.050	0.255	0.277	0.117	0.035		0.088	0.001	0.310	0.228	0.028			
		Min	-0.139	0.267	0.030	0.000	0.924		-0.222	-0.003	0.090	0.130	0.000			
		Max	0.106	1.538	0.956	0.671	1.092		0.225	0.003	1.459	0.962	0.174			
PT	*q=0*	Mean	0.107	2.766	0.951	0.000	0.923	100	0.119	0.000	2.736	0.964	0.000	100	555	29.0
		Std. Err.	0.018	0.086	0.029	0.000	0.013		0.037	0.001	0.112	0.020	0.000			
		Min	0.054	2.562	0.826	0.000	0.892		-0.007	-0.001	2.463	0.909	0.000			
		Max	0.148	3.002	0.987	0.000	0.962		0.201	0.002	3.103	0.991	0.000			
	q=1	Mean	0.027	1.830	0.523	0.042	0.981	92	0.050	0.000	1.773	0.656	0.009	98	123	7.1
		Std. Err.	0.031	0.154	0.258	0.165	0.022		0.054	0.001	0.196	0.213	0.065			
		Min	-0.026	1.359	0.001	0.000	0.915		-0.071	-0.002	1.250	0.070	0.000			
		Max	0.118	2.096	0.907	0.993	1.018		0.194	0.002	2.160	0.971	0.599			
	q=2	Mean	0.030	1.555	0.484	0.055	0.978	90	0.051	0.000	1.502	0.622	0.009	98	137	5.5
		Std. Err.	0.049	0.248	0.259	0.188	0.035		0.081	0.001	0.297	0.220	0.074			
		Min	-0.046	0.785	0.008	0.000	0.869		-0.125	-0.003	0.708	0.054	0.000			
		Max	0.178	1.960	0.941	0.913	1.032		0.261	0.003	2.042	0.968	0.736			
	q=3	Mean	0.028	1.424	0.482	0.056	0.980	87	0.045	0.000	1.381	0.622	0.012	97	148	4.8
		Std. Err.	0.059	0.301	0.269	0.167	0.042		0.095	0.001	0.348	0.222	0.078			
		Min	-0.066	0.545	0.001	0.000	0.856		-0.153	-0.003	0.524	0.060	0.000			
		Max	0.194	1.893	0.955	0.985	1.044		0.281	0.004	1.980	0.964	0.691			
SAL	*q=0*	Mean	0.120	2.776	0.951	0.000	0.913	100	0.126	0.000	2.761	0.964	0.000	100	1084	33.7
		Std. Err.	0.019	0.091	0.027	0.000	0.015		0.044	0.001	0.122	0.018	0.000			
		Min	0.075	2.572	0.836	0.000	0.878		0.034	-0.002	2.500	0.897	0.000			
		Max	0.166	2.992	0.989	0.000	0.947		0.214	0.002	2.999	0.990	0.000			
		Mean	0.018	2.031	0.577	0.017	0.988	95	0.036	0.000	1.986	0.708	0.001	100	100	8.3

	q=1	Std. Err.	0.017	0.083	0.254	0.093	0.012		0.030	0.000	0.105	0.210	0.004			
		Min	-0.018	1.824	0.016	0.000	0.959		-0.042	-0.002	1.758	0.192	0.000			
		Max	0.058	2.209	0.961	0.822	1.012		0.110	0.001	2.242	0.977	0.029			
	q=2	Mean	0.017	1.790	0.513	0.054	0.988	85	0.033	0.000	1.750	0.656	0.011	98	105	6.5
		Std. Err.	0.020	0.098	0.286	0.155	0.014		0.037	0.000	0.127	0.220	0.093			
		Min	-0.027	1.535	0.008	0.000	0.953		-0.061	-0.002	1.489	0.031	0.000			
		Max	0.066	2.001	0.943	0.916	1.018		0.127	0.001	2.049	0.959	0.912			
	q=3	Mean	0.016	1.666	0.513	0.075	0.988	85	0.032	0.000	1.629	0.648	0.008	97	107	5.7
		Std. Err.	0.021	0.104	0.282	0.204	0.015		0.040	0.001	0.137	0.215	0.045			
		Min	-0.029	1.405	0.003	0.000	0.953		-0.079	-0.002	1.361	0.105	0.000			
		Max	0.066	1.897	0.928	0.967	1.020		0.129	0.001	1.974	0.965	0.348			
Stool	*q=0*	Mean	0.128	2.648	0.899	0.000	0.907	100	0.201	-0.001	2.463	0.949	0.000	100	165	26.7
		Std. Err.	0.026	0.128	0.054	0.000	0.020		0.060	0.001	0.194	0.033	0.000			
		Min	0.059	2.311	0.713	0.000	0.852		0.051	-0.003	1.946	0.853	0.000			
		Max	0.200	3.016	0.984	0.000	0.959		0.343	0.001	2.921	0.992	0.000			
	q=1	Mean	0.016	0.845	0.519	0.057	0.989	87	0.035	0.000	0.798	0.678	0.005	99	111	2.5
		Std. Err.	0.030	0.150	0.281	0.169	0.021		0.053	0.001	0.191	0.206	0.049			
		Min	-0.082	0.530	0.003	0.000	0.945		-0.128	-0.002	0.446	0.081	0.000			
		Max	0.078	1.348	0.964	0.969	1.055		0.131	0.001	1.436	0.973	0.486			
	q=2	Mean	0.008	0.667	0.470	0.048	0.994	84	0.022	0.000	0.632	0.643	0.010	98	93	2.0
		Std. Err.	0.029	0.145	0.263	0.148	0.020		0.055	0.001	0.195	0.202	0.093			
		Min	-0.080	0.307	0.009	0.000	0.946		-0.143	-0.002	0.236	0.027	0.000			
		Max	0.077	1.100	0.965	0.898	1.054		0.140	0.001	1.262	0.966	0.922			
	q=3	Mean	0.007	0.609	0.462	0.062	0.995	84	0.020	0.000	0.576	0.638	0.010	99	90	1.9
		Std. Err.	0.031	0.154	0.271	0.184	0.022		0.058	0.001	0.205	0.212	0.099			
		Min	-0.080	0.224	0.008	0.000	0.944		-0.154	-0.002	0.142	0.008	0.000			
		Max	0.079	1.033	0.967	0.910	1.054		0.154	0.001	1.220	0.971	0.993			
SubP	*q=0*	Mean	0.097	2.811	0.941	0.000	0.931	100	0.107	0.000	2.785	0.953	0.000	100	564	28.7
		Std. Err.	0.018	0.086	0.032	0.000	0.013		0.036	0.000	0.111	0.028	0.000			
		Min	0.060	2.603	0.790	0.000	0.900		0.026	-0.001	2.501	0.820	0.000			
		Max	0.137	3.010	0.984	0.000	0.958		0.190	0.001	3.045	0.992	0.000			
	q=1	Mean	0.023	2.081	0.625	0.014	0.984	95	0.048	0.000	2.020	0.753	0.000	100	108	9.0
		Std. Err.	0.018	0.091	0.231	0.067	0.013		0.038	0.000	0.131	0.177	0.004			
		Min	-0.025	1.807	0.055	0.000	0.943		-0.039	-0.002	1.644	0.174	0.000			
		Max	0.080	2.319	0.952	0.430	1.017		0.163	0.001	2.354	0.964	0.044			
	q=2	Mean	0.033	1.891	0.616	0.019	0.977	94	0.066	-0.001	1.810	0.751	0.002	99	110	7.8
		Std. Err.	0.026	0.127	0.232	0.094	0.018		0.048	0.001	0.173	0.172	0.021			
		Min	-0.021	1.494	0.030	0.000	0.919		-0.034	-0.002	1.332	0.123	0.000			
		Max	0.113	2.159	0.936	0.671	1.015		0.215	0.001	2.192	0.971	0.211			
	q=3	Mean	0.039	1.786	0.589	0.031	0.972	93	0.077	-0.001	1.693	0.735	0.000	100	113	7.2
		Std. Err.	0.031	0.158	0.251	0.141	0.023		0.055	0.001	0.202	0.177	0.001			
		Min	-0.018	1.306	0.001	0.000	0.903		-0.029	-0.003	1.136	0.219	0.000			
		Max	0.134	2.079	0.950	0.992	1.013		0.250	0.001	2.098	0.975	0.007			
SupP	*q=0*	Mean	0.063	2.817	0.858	0.000	0.956	100	0.100	-0.001	2.724	0.912	0.000	100	151	22.8
		Std. Err.	0.016	0.077	0.082	0.000	0.012		0.033	0.000	0.108	0.061	0.000			
		Min	0.025	2.636	0.579	0.000	0.927		0.018	-0.002	2.445	0.658	0.000			
		Max	0.101	3.001	0.963	0.000	0.983		0.194	0.000	3.019	0.984	0.000			
	q=1	Mean	0.016	1.990	0.489	0.077	0.989	83	0.037	0.000	1.938	0.662	0.004	99	100	7.9
		Std. Err.	0.028	0.138	0.280	0.213	0.019		0.049	0.001	0.177	0.206	0.037			
		Min	-0.034	1.593	0.008	0.000	0.936		-0.087	-0.002	1.490	0.098	0.000			
		Max	0.090	2.254	0.946	0.911	1.024		0.154	0.002	2.250	0.961	0.370			
	q=2	Mean	0.017	1.789	0.501	0.036	0.988	88	0.040	0.000	1.731	0.662	0.003	98	98	6.5
		Std. Err.	0.039	0.196	0.257	0.126	0.027		0.065	0.001	0.240	0.195	0.023			
		Min	-0.047	1.134	0.020	0.000	0.897		-0.142	-0.003	1.072	0.124	0.000			
		Max	0.141	2.130	0.936	0.776	1.032		0.213	0.003	2.104	0.964	0.202			
	q=3	Mean	0.014	1.678	0.511	0.017	0.990	93	0.037	0.000	1.622	0.662	0.001	99	92	5.8
		Std. Err.	0.045	0.232	0.242	0.064	0.032		0.074	0.001	0.272	0.191	0.008			
		Min	-0.060	0.909	0.056	0.000	0.884		-0.180	-0.003	0.870	0.156	0.000			
		Max	0.159	2.079	0.947	0.417	1.041		0.238	0.003	2.059	0.960	0.076			
TD	*q=0*	Mean	0.099	2.691	0.932	0.000	0.929	100	0.111	0.000	2.661	0.946	0.000	100	519	25.6
		Std. Err.	0.020	0.091	0.038	0.000	0.015		0.042	0.001	0.127	0.033	0.000			
		Min	0.056	2.419	0.749	0.000	0.886		0.036	-0.002	2.284	0.752	0.000			
		Max	0.155	2.903	0.989	0.000	0.960		0.249	0.001	2.883	0.990	0.000			
	q=1	Mean	0.020	1.912	0.533	0.026	0.986	92	0.045	0.000	1.851	0.688	0.001	99	104	7.5
		Std. Err.	0.021	0.100	0.252	0.110	0.015		0.042	0.000	0.145	0.199	0.006			
		Min	-0.025	1.635	0.014	0.000	0.944		-0.031	-0.003	1.352	0.165	0.000			
		Max	0.078	2.126	0.932	0.836	1.017		0.217	0.000	2.142	0.968	0.056			
	q=2	Mean	0.026	1.737	0.501	0.052	0.982	86	0.055	-0.001	1.662	0.660	0.010	96	106	6.5
		Std. Err.	0.029	0.144	0.267	0.156	0.021		0.057	0.001	0.200	0.218	0.061			
		Min	-0.027	1.345	0.018	0.000	0.923		-0.030	-0.003	0.982	0.078	0.000			
		Max	0.107	1.985	0.904	0.797	1.019		0.288	0.001	1.993	0.967	0.527			
	q=3	Mean	0.028	1.643	0.489	0.067	0.980	87	0.060	-0.001	1.563	0.648	0.003	98	106	5.9
		Std. Err.	0.036	0.178	0.261	0.211	0.026		0.067	0.001	0.237	0.210	0.020			
		Min	-0.031	1.188	0.005	0.000	0.912		-0.036	-0.004	0.807	0.128	0.000			
		Max	0.122	1.953	0.876	0.941	1.021		0.322	0.001	1.921	0.964	0.179			

Th	*q=0*	Mean	0.166	2.641	0.966	0.000	0.878	100	0.141	0.000	2.704	0.976	0.000	100	0	NA
		Std. Err.	0.022	0.109	0.019	0.000	0.017		0.042	0.001	0.121	0.014	0.000			
		Min	0.106	2.386	0.899	0.000	0.842		0.060	-0.001	2.378	0.910	0.000			
		Max	0.212	2.910	0.991	0.000	0.924		0.266	0.002	2.979	0.993	0.000			
	q=1	Mean	0.020	1.841	0.494	0.042	0.986	89	0.043	0.000	1.784	0.656	0.008	97	102	7.0
		Std. Err.	0.032	0.158	0.261	0.146	0.022		0.054	0.001	0.199	0.215	0.050			
		Min	-0.041	1.349	0.009	0.000	0.915		-0.057	-0.002	1.214	0.089	0.000			
		Max	0.118	2.133	0.966	0.897	1.028		0.189	0.001	2.177	0.985	0.449			
	q=2	Mean	0.022	1.582	0.443	0.075	0.984	85	0.047	0.000	1.521	0.617	0.009	96	103	5.4
		Std. Err.	0.049	0.249	0.287	0.216	0.035		0.080	0.001	0.297	0.235	0.047			
		Min	-0.075	0.846	0.007	0.000	0.875		-0.071	-0.004	0.656	0.094	0.000			
		Max	0.169	2.062	0.959	0.923	1.050		0.297	0.002	2.019	0.982	0.411			
	q=3	Mean	0.019	1.456	0.457	0.073	0.986	82	0.042	0.000	1.399	0.620	0.025	96	100	4.7
		Std. Err.	0.059	0.298	0.287	0.201	0.042		0.092	0.001	0.343	0.239	0.130			
		Min	-0.099	0.609	0.003	0.000	0.861		-0.102	-0.004	0.466	0.038	0.000			
		Max	0.187	2.044	0.956	0.971	1.067		0.328	0.002	1.949	0.977	0.868			

* The model parameters were computed from 100 times of repeated DAR modeling from 100 times of re-sampling; *N* is the number of successful DAR model-fitting out of 100 times of re-sampling.

Table S4. Fitting the DAR (Diversity-Area Relationship) models for each of the 10 digestive microbiome sites at the ***order*** taxon-level

Digestive Site	Diversity Order & Statistics		Power Law (PL)						PL with Exponential Cutoff (PLEC)							
			z	ln(*c*)	*R*	*p*-value	*g*	**N*	*z*	*d*	ln(*c*)	*R*	*p*-value	**N*	A_{max}	D_{max}
BM	*q=0*	Mean	0.171	3.002	0.972	0.000	0.874	100	0.188	0.000	2.959	0.977	0.000	100	600	53.2
		Std. Err.	0.022	0.109	0.017	0.000	0.017		0.042	0.001	0.141	0.015	0.000			
		Min	0.115	2.735	0.903	0.000	0.830		0.095	-0.002	2.652	0.903	0.000			
		Max	0.226	3.309	0.994	0.000	0.917		0.288	0.001	3.298	0.995	0.000			
	q=1	Mean	0.018	1.558	0.509	0.039	0.987	90	0.036	0.000	1.514	0.661	0.001	99	112	5.2
		Std. Err.	0.040	0.198	0.254	0.147	0.028		0.077	0.001	0.267	0.205	0.006			
		Min	-0.089	1.131	0.003	0.000	0.927		-0.135	-0.003	0.894	0.168	0.000			
		Max	0.101	2.061	0.922	0.962	1.060		0.240	0.001	2.175	0.978	0.055			
	q=2	Mean	0.006	1.079	0.484	0.048	0.995	85	0.012	0.000	1.064	0.643	0.004	98	115	3.0
		Std. Err.	0.049	0.243	0.264	0.153	0.034		0.091	0.001	0.319	0.210	0.033			
		Min	-0.137	0.600	0.010	0.000	0.923		-0.223	-0.004	0.342	0.104	0.000			
		Max	0.108	1.788	0.935	0.881	1.091		0.264	0.002	1.965	0.974	0.326			
	q=3	Mean	0.001	0.892	0.476	0.054	0.999	86	0.001	0.000	0.892	0.639	0.010	98	206	2.4
		Std. Err.	0.046	0.227	0.267	0.155	0.032		0.085	0.001	0.301	0.210	0.087			
		Min	-0.140	0.461	0.009	0.000	0.934		-0.236	-0.003	0.239	0.039	0.000			
		Max	0.092	1.613	0.938	0.893	1.092		0.230	0.002	1.785	0.973	0.856			
HP	*q=0*	Mean	0.170	3.061	0.967	0.000	0.874	100	0.212	-0.001	2.958	0.979	0.000	100	278	51.4
		Std. Err.	0.022	0.110	0.017	0.000	0.017		0.044	0.001	0.146	0.013	0.000			
		Min	0.113	2.820	0.914	0.000	0.835		0.119	-0.002	2.580	0.916	0.000			
		Max	0.221	3.386	0.992	0.000	0.918		0.334	0.001	3.280	0.996	0.000			
	q=1	Mean	0.016	1.694	0.492	0.059	0.989	89	0.025	0.000	1.673	0.639	0.017	97	157	5.9
		Std. Err.	0.035	0.172	0.258	0.203	0.024		0.064	0.001	0.228	0.199	0.105			
		Min	-0.059	1.240	0.004	0.000	0.924		-0.118	-0.002	1.116	0.048	0.000			
		Max	0.106	2.041	0.936	0.959	1.040		0.156	0.002	2.185	0.964	0.796			
	q=2	Mean	0.005	1.260	0.493	0.036	0.996	86	-0.001	0.000	1.274	0.633	0.015	97	9	3.6
		Std. Err.	0.050	0.249	0.264	0.110	0.035		0.089	0.001	0.321	0.216	0.091			
		Min	-0.126	0.670	0.032	0.000	0.913		-0.240	-0.002	0.583	0.061	0.000			
		Max	0.120	1.867	0.955	0.647	1.083		0.170	0.003	2.148	0.966	0.683			
	q=3	Mean	-0.002	1.070	0.498	0.041	1.001	90	-0.015	0.000	1.102	0.638	0.002	99	65	2.9
		Std. Err.	0.051	0.252	0.262	0.149	0.035		0.091	0.001	0.329	0.213	0.009			
		Min	-0.148	0.503	0.001	0.000	0.922		-0.285	-0.002	0.445	0.162	0.000			
		Max	0.108	1.748	0.961	0.990	1.097		0.156	0.003	2.086	0.970	0.070			
KG	*q=0*	Mean	0.199	2.736	0.965	0.000	0.852	100	0.255	-0.001	2.596	0.980	0.000	100	253	42.7
		Std. Err.	0.028	0.139	0.021	0.000	0.023		0.057	0.001	0.188	0.014	0.000			
		Min	0.117	2.360	0.890	0.000	0.792		0.129	-0.003	2.042	0.905	0.000			
		Max	0.272	3.123	0.993	0.000	0.915		0.400	0.001	3.062	0.996	0.000			
	q=1	Mean	0.027	1.424	0.448	0.070	0.980	84	0.057	-0.001	1.351	0.602	0.009	97	108	4.8
		Std. Err.	0.045	0.222	0.272	0.191	0.032		0.081	0.001	0.286	0.228	0.056			
		Min	-0.072	0.555	0.008	0.000	0.852		-0.097	-0.003	0.408	0.083	0.000			
		Max	0.200	1.962	0.979	0.914	1.049		0.279	0.001	1.948	0.991	0.493			
	q=2	Mean	0.009	1.072	0.422	0.090	0.993	75	0.025	0.000	1.032	0.582	0.025	92	86	3.1
		Std. Err.	0.050	0.251	0.280	0.204	0.035		0.088	0.001	0.315	0.241	0.106			
		Min	-0.112	0.192	0.013	0.000	0.866		-0.152	-0.003	0.102	0.042	0.000			
		Max	0.182	1.731	0.987	0.851	1.075		0.229	0.002	1.711	0.993	0.837			
	q=3	Mean	0.000	0.928	0.441	0.051	0.999	84	0.009	0.000	0.906	0.592	0.012	97	59	2.5
		Std. Err.	0.048	0.241	0.261	0.159	0.034		0.085	0.001	0.305	0.228	0.091			
		Min	-0.123	0.132	0.001	0.000	0.885		-0.158	-0.002	0.056	0.033	0.000			
		Max	0.157	1.600	0.987	0.994	1.082		0.194	0.002	1.554	0.992	0.896			
PT	*q=0*	Mean	0.157	3.042	0.959	0.000	0.885	100	0.210	-0.001	2.910	0.980	0.000	100	224	46.4
		Std. Err.	0.025	0.123	0.026	0.000	0.019		0.049	0.001	0.161	0.013	0.000			
		Min	0.096	2.777	0.874	0.000	0.838		0.103	-0.002	2.461	0.928	0.000			
		Max	0.216	3.343	0.992	0.000	0.931		0.345	0.001	3.325	0.995	0.000			
	q=1	Mean	0.026	1.957	0.566	0.036	0.982	91	0.053	0.000	1.888	0.726	0.000	100	108	8.0
		Std. Err.	0.029	0.144	0.262	0.144	0.021		0.055	0.001	0.199	0.178	0.000			
		Min	-0.032	1.514	0.005	0.000	0.916		-0.046	-0.002	1.224	0.318	0.000			
		Max	0.117	2.224	0.929	0.938	1.022		0.233	0.001	2.238	0.955	0.000			
	q=2	Mean	0.029	1.669	0.522	0.050	0.979	88	0.060	-0.001	1.593	0.682	0.010	96	109	6.1
		Std. Err.	0.046	0.228	0.260	0.176	0.033		0.082	0.001	0.297	0.205	0.050			
		Min	-0.059	0.954	0.001	0.000	0.870		-0.094	-0.003	0.608	0.099	0.000			
		Max	0.176	2.083	0.921	0.992	1.040		0.323	0.001	2.092	0.959	0.362			
	q=3	Mean	0.028	1.536	0.498	0.058	0.980	86	0.059	-0.001	1.457	0.670	0.004	98	105	5.3
		Std. Err.	0.055	0.277	0.267	0.178	0.039		0.095	0.001	0.341	0.199	0.036			
		Min	-0.077	0.719	0.002	0.000	0.855		-0.134	-0.004	0.397	0.099	0.000			
		Max	0.195	2.027	0.929	0.976	1.052		0.353	0.002	2.031	0.975	0.358			
SAL	*q=0*	Mean	0.201	2.985	0.977	0.000	0.850	100	0.206	0.000	2.975	0.984	0.000	100	2471	79.6
		Std. Err.	0.023	0.111	0.014	0.000	0.018		0.053	0.001	0.148	0.008	0.000			
		Min	0.151	2.730	0.928	0.000	0.805		0.075	-0.002	2.565	0.955	0.000			

		Max	0.257	3.247	0.996	0.000	0.890		0.326	0.002	3.282	0.996	0.000			
	q=1	Mean	0.019	2.118	0.583	0.011	0.986	95	0.044	0.000	2.059	0.727	0.005	99	92	9.1
		Std. Err.	0.022	0.108	0.241	0.047	0.015		0.041	0.000	0.146	0.198	0.044			
		Min	-0.034	1.880	0.073	0.000	0.953		-0.036	-0.002	1.736	0.093	0.000			
		Max	0.066	2.378	0.959	0.313	1.023		0.147	0.000	2.383	0.982	0.439			
	q=2	Mean	0.017	1.833	0.544	0.020	0.988	92	0.042	0.000	1.772	0.693	0.003	99	85	6.8
		Std. Err.	0.028	0.138	0.256	0.073	0.020		0.050	0.001	0.179	0.207	0.026			
		Min	-0.045	1.515	0.060	0.000	0.945		-0.072	-0.002	1.376	0.119	0.000			
		Max	0.077	2.141	0.949	0.411	1.031		0.172	0.001	2.181	0.973	0.262			
	q=3	Mean	0.016	1.698	0.521	0.046	0.989	90	0.041	0.000	1.636	0.673	0.001	99	83	5.9
		Std. Err.	0.030	0.147	0.266	0.164	0.021		0.052	0.001	0.188	0.215	0.006			
		Min	-0.051	1.358	0.006	0.000	0.940		-0.093	-0.002	1.242	0.172	0.000			
		Max	0.083	2.020	0.948	0.930	1.035		0.170	0.001	2.109	0.965	0.058			
Stool	*q=0*	Mean	0.226	2.672	0.959	0.000	0.830	100	0.289	-0.001	2.509	0.976	0.000	100	272	46.6
		Std. Err.	0.037	0.191	0.024	0.000	0.030		0.072	0.001	0.247	0.015	0.000			
		Min	0.138	2.282	0.862	0.000	0.764		0.107	-0.003	1.912	0.916	0.000			
		Max	0.306	3.100	0.993	0.000	0.900		0.489	0.001	3.032	0.994	0.000			
	q=1	Mean	0.012	0.818	0.501	0.037	0.992	89	0.029	0.000	0.775	0.663	0.004	99	103	2.4
		Std. Err.	0.026	0.138	0.256	0.127	0.018		0.042	0.001	0.155	0.190	0.035			
		Min	-0.060	0.424	0.019	0.000	0.935		-0.068	-0.002	0.297	0.097	0.000			
		Max	0.090	1.187	0.922	0.773	1.040		0.140	0.001	1.194	0.960	0.351			
	q=2	Mean	0.006	0.654	0.458	0.043	0.996	87	0.020	0.000	0.618	0.625	0.010	97	84	2.0
		Std. Err.	0.027	0.137	0.250	0.137	0.019		0.046	0.001	0.168	0.204	0.064			
		Min	-0.052	0.223	0.015	0.000	0.935		-0.091	-0.002	0.105	0.072	0.000			
		Max	0.090	0.960	0.941	0.822	1.035		0.137	0.001	1.008	0.974	0.559			
	q=3	Mean	0.004	0.600	0.462	0.052	0.997	89	0.018	0.000	0.565	0.632	0.002	99	79	1.9
		Std. Err.	0.029	0.150	0.261	0.163	0.020		0.051	0.001	0.182	0.202	0.011			
		Min	-0.056	0.153	0.011	0.000	0.935		-0.095	-0.002	0.054	0.141	0.000			
		Max	0.091	0.895	0.952	0.872	1.038		0.135	0.001	0.948	0.976	0.108			
SubP	*q=0*	Mean	0.145	3.115	0.956	0.000	0.894	100	0.190	-0.001	3.004	0.975	0.000	100	235	47.0
		Std. Err.	0.025	0.125	0.028	0.000	0.019		0.043	0.001	0.148	0.018	0.000			
		Min	0.075	2.738	0.849	0.000	0.833		0.084	-0.002	2.675	0.900	0.000			
		Max	0.222	3.457	0.992	0.000	0.947		0.268	0.001	3.396	0.995	0.000			
	q=1	Mean	0.026	2.158	0.623	0.028	0.981	94	0.056	-0.001	2.084	0.761	0.010	97	104	9.9
		Std. Err.	0.018	0.086	0.218	0.134	0.013		0.039	0.000	0.132	0.181	0.072			
		Min	-0.009	1.856	0.009	0.000	0.936		-0.028	-0.002	1.578	0.062	0.000			
		Max	0.089	2.332	0.930	0.903	1.006		0.202	0.000	2.350	0.971	0.673			
	q=2	Mean	0.032	1.938	0.595	0.012	0.977	97	0.069	-0.001	1.847	0.742	0.000	100	104	8.2
		Std. Err.	0.024	0.118	0.217	0.081	0.017		0.052	0.001	0.175	0.172	0.000			
		Min	-0.013	1.583	0.028	0.000	0.924		-0.027	-0.002	1.264	0.257	0.000			
		Max	0.105	2.177	0.950	0.690	1.009		0.234	0.001	2.161	0.964	0.001			
	q=3	Mean	0.036	1.823	0.567	0.009	0.974	96	0.076	-0.001	1.726	0.720	0.002	99	106	7.4
		Std. Err.	0.029	0.143	0.230	0.050	0.021		0.060	0.001	0.203	0.186	0.020			
		Min	-0.018	1.451	0.056	0.000	0.918		-0.029	-0.002	1.129	0.125	0.000			
		Max	0.114	2.118	0.932	0.425	1.013		0.241	0.001	2.062	0.960	0.200			
SupP	*q=0*	Mean	0.120	3.083	0.929	0.000	0.913	100	0.184	-0.001	2.924	0.970	0.000	100	163	39.6
		Std. Err.	0.021	0.101	0.039	0.000	0.016		0.042	0.001	0.141	0.021	0.000			
		Min	0.066	2.803	0.800	0.000	0.868		0.107	-0.002	2.549	0.904	0.000			
		Max	0.179	3.349	0.989	0.000	0.953		0.297	0.000	3.222	0.996	0.000			
	q=1	Mean	0.025	2.063	0.505	0.068	0.982	88	0.051	0.000	1.999	0.679	0.029	96	112	8.9
		Std. Err.	0.025	0.123	0.281	0.206	0.018		0.051	0.001	0.176	0.221	0.145			
		Min	-0.022	1.740	0.002	0.000	0.935		-0.052	-0.002	1.552	0.030	0.000			
		Max	0.091	2.295	0.916	0.973	1.015		0.173	0.001	2.323	0.959	0.912			
	q=2	Mean	0.026	1.807	0.475	0.088	0.982	84	0.051	0.000	1.744	0.657	0.007	97	114	6.9
		Std. Err.	0.036	0.181	0.271	0.232	0.025		0.071	0.001	0.245	0.201	0.049			
		Min	-0.043	1.324	0.002	0.000	0.916		-0.097	-0.003	1.117	0.086	0.000			
		Max	0.116	2.130	0.914	0.972	1.029		0.270	0.002	2.159	0.951	0.459			
	q=3	Mean	0.021	1.672	0.464	0.070	0.985	82	0.043	0.000	1.618	0.652	0.002	99	113	5.9
		Std. Err.	0.042	0.213	0.271	0.196	0.030		0.082	0.001	0.278	0.189	0.013			
		Min	-0.060	1.097	0.005	0.000	0.907		-0.125	-0.004	0.863	0.139	0.000			
		Max	0.128	2.056	0.907	0.938	1.041		0.311	0.002	2.104	0.951	0.132			
TD	*q=0*	Mean	0.157	2.902	0.966	0.000	0.885	100	0.199	-0.001	2.797	0.980	0.000	100	268	40.8
		Std. Err.	0.025	0.128	0.020	0.000	0.019		0.042	0.001	0.151	0.011	0.000			
		Min	0.096	2.581	0.904	0.000	0.835		0.101	-0.002	2.422	0.933	0.000			
		Max	0.221	3.226	0.992	0.000	0.931		0.305	0.000	3.160	0.995	0.000			
	q=1	Mean	0.021	1.986	0.584	0.031	0.985	93	0.043	0.000	1.929	0.711	0.006	99	109	8.1
		Std. Err.	0.019	0.093	0.244	0.145	0.013		0.037	0.000	0.133	0.201	0.052			
		Min	-0.018	1.688	0.009	0.000	0.942		-0.027	-0.002	1.441	0.079	0.000			
		Max	0.081	2.178	0.965	0.893	1.012		0.180	0.001	2.189	0.973	0.521			
	q=2	Mean	0.026	1.784	0.544	0.024	0.982	93	0.051	0.000	1.720	0.679	0.009	98	113	6.8
		Std. Err.	0.027	0.134	0.234	0.104	0.019		0.053	0.001	0.187	0.194	0.080			
		Min	-0.025	1.374	0.032	0.000	0.923		-0.060	-0.002	1.096	0.047	0.000			
		Max	0.107	2.036	0.968	0.641	1.017		0.218	0.001	2.068	0.968	0.792			
	q=3	Mean	0.028	1.683	0.520	0.032	0.980	91	0.055	0.000	1.617	0.666	0.000	100	117	6.2
		Std. Err.	0.033	0.165	0.236	0.118	0.024		0.064	0.001	0.223	0.184	0.000			

		Min	-0.030	1.223	0.029	0.000	0.913		-0.081	-0.002	0.938	0.240	0.000			
		Max	0.120	1.973	0.965	0.679	1.021		0.233	0.001	2.032	0.970	0.002			
Th	*q=0*	Mean	0.231	2.954	0.981	0.000	0.826	100	0.226	0.000	2.969	0.985	0.000	100	0	NA
		Std. Err.	0.027	0.139	0.011	0.000	0.022		0.046	0.001	0.152	0.010	0.000			
		Min	0.171	2.612	0.936	0.000	0.773		0.118	-0.002	2.488	0.936	0.000			
		Max	0.295	3.283	0.996	0.000	0.874		0.383	0.002	3.353	0.997	0.000			
	q=1	Mean	0.027	1.947	0.572	0.042	0.981	89	0.054	0.000	1.881	0.715	0.002	99	111	8.0
		Std. Err.	0.029	0.144	0.263	0.156	0.020		0.054	0.001	0.193	0.195	0.012			
		Min	-0.045	1.423	0.008	0.000	0.905		-0.054	-0.002	1.222	0.148	0.000			
		Max	0.132	2.328	0.945	0.905	1.031		0.225	0.001	2.272	0.968	0.108			
	q=2	Mean	0.030	1.637	0.522	0.052	0.979	89	0.060	-0.001	1.563	0.675	0.007	97	109	6.0
		Std. Err.	0.043	0.220	0.274	0.165	0.031		0.075	0.001	0.273	0.214	0.050			
		Min	-0.060	0.885	0.013	0.000	0.870		-0.095	-0.003	0.717	0.088	0.000			
		Max	0.176	2.102	0.956	0.859	1.041		0.277	0.002	2.059	0.975	0.460			
	q=3	Mean	0.031	1.488	0.516	0.044	0.978	88	0.061	-0.001	1.416	0.668	0.008	98	111	5.2
		Std. Err.	0.051	0.262	0.268	0.157	0.036		0.084	0.001	0.308	0.212	0.075			
		Min	-0.077	0.663	0.006	0.000	0.860		-0.120	-0.003	0.561	0.053	0.000			
		Max	0.189	2.031	0.970	0.936	1.052		0.283	0.002	1.947	0.976	0.752			

* The model parameters were computed from 100 times of repeated DAR modeling from 100 times of re-sampling; *N* is the number of successful DAR model-fitting out of 100 times of re-sampling.

Table S5. Fitting the DAR (Diversity-Area Relationship) models for each of the 10 digestive microbiome sites at the ***family*** taxon-level

Site	Diversity Order & Statistics		Power Law (PL)						PL with Exponential Cutoff (PLEC)							
			z	ln(*c*)	*R*	*p*-value	*g*	**N*	*z*	*d*	ln(*c*)	*R*	*p*-value	**N*	A_{max}	D_{max}
BM	*q=0*	Mean	0.190	3.584	0.983	0.000	0.859	100	0.192	0.000	3.578	0.987	0.000	100	4526	149.3
		Std. Err.	0.020	0.099	0.010	0.000	0.016		0.041	0.001	0.131	0.009	0.000			
		Min	0.130	3.347	0.944	0.000	0.821		0.076	-0.001	3.285	0.956	0.000			
		Max	0.238	3.893	0.996	0.000	0.906		0.300	0.002	3.935	0.996	0.000			
	q=1	Mean	0.023	1.800	0.480	0.079	0.984	84	0.044	0.000	1.746	0.664	0.010	96	112	6.8
		Std. Err.	0.049	0.240	0.288	0.229	0.035		0.098	0.001	0.336	0.220	0.053			
		Min	-0.110	1.056	0.001	0.000	0.872		-0.213	-0.003	0.899	0.102	0.000			
		Max	0.174	2.401	0.963	0.984	1.074		0.255	0.002	2.657	0.973	0.347			
	q=2	Mean	0.003	1.172	0.469	0.035	0.997	89	0.007	0.000	1.163	0.634	0.007	97	96	3.3
		Std. Err.	0.051	0.246	0.263	0.116	0.035		0.103	0.001	0.350	0.233	0.040			
		Min	-0.153	0.457	0.033	0.000	0.892		-0.293	-0.003	0.402	0.096	0.000			
		Max	0.148	1.893	0.975	0.641	1.101		0.207	0.003	2.239	0.977	0.387			
	q=3	Mean	-0.003	0.957	0.473	0.045	1.001	90	-0.005	0.000	0.962	0.637	0.013	96	119	2.6
		Std. Err.	0.045	0.217	0.261	0.174	0.031		0.093	0.001	0.316	0.229	0.074			
		Min	-0.147	0.344	0.000	0.000	0.912		-0.287	-0.003	0.310	0.075	0.000			
		Max	0.122	1.625	0.976	0.997	1.097		0.173	0.003	1.973	0.977	0.564			
HP	*q=0*	Mean	0.221	3.596	0.987	0.000	0.834	100	0.237	0.000	3.556	0.991	0.000	100	800	134.7
		Std. Err.	0.022	0.118	0.008	0.000	0.018		0.037	0.001	0.122	0.005	0.000			
		Min	0.141	3.375	0.957	0.000	0.800		0.138	-0.002	3.301	0.966	0.000			
		Max	0.263	4.030	0.997	0.000	0.898		0.313	0.001	3.952	0.999	0.000			
	q=1	Mean	0.017	2.105	0.477	0.078	0.987	85	0.039	0.000	2.051	0.645	0.003	98	97	8.9
		Std. Err.	0.048	0.240	0.279	0.220	0.034		0.088	0.001	0.309	0.218	0.019			
		Min	-0.083	1.425	0.008	0.000	0.888		-0.167	-0.003	1.254	0.139	0.000			
		Max	0.153	2.572	0.965	0.911	1.056		0.282	0.003	2.750	0.980	0.140			
	q=2	Mean	-0.009	1.484	0.503	0.023	1.006	90	-0.008	0.000	1.479	0.649	0.002	98	0	NA
		Std. Err.	0.062	0.312	0.258	0.087	0.043		0.111	0.001	0.393	0.215	0.011			
		Min	-0.135	0.662	0.027	0.000	0.888		-0.274	-0.003	0.549	0.154	0.000			
		Max	0.153	2.117	0.969	0.707	1.090		0.271	0.004	2.414	0.976	0.090			
	q=3	Mean	-0.016	1.231	0.516	0.038	1.011	92	-0.024	0.000	1.248	0.661	0.004	99	179	3.2
		Std. Err.	0.059	0.294	0.258	0.147	0.040		0.106	0.001	0.376	0.207	0.032			
		Min	-0.145	0.491	0.008	0.000	0.906		-0.300	-0.003	0.401	0.106	0.000			
		Max	0.130	1.832	0.969	0.910	1.095		0.230	0.004	2.215	0.974	0.320			
KG	*q=0*	Mean	0.198	3.402	0.960	0.000	0.853	100	0.266	-0.001	3.231	0.981	0.000	100	216	81.1
		Std. Err.	0.029	0.146	0.027	0.000	0.024		0.053	0.001	0.183	0.016	0.000			
		Min	0.138	3.029	0.819	0.000	0.791		0.119	-0.003	2.776	0.877	0.000			
		Max	0.273	3.711	0.996	0.000	0.899		0.404	0.001	3.686	0.997	0.000			
	q=1	Mean	0.028	1.680	0.509	0.040	0.979	89	0.064	-0.001	1.592	0.647	0.008	95	101	6.2
		Std. Err.	0.063	0.312	0.265	0.147	0.045		0.116	0.001	0.413	0.227	0.039			
		Min	-0.090	0.870	0.006	0.000	0.869		-0.148	-0.004	0.477	0.105	0.000			
		Max	0.178	2.276	0.943	0.931	1.060		0.400	0.002	2.351	0.945	0.320			
	q=2	Mean	0.000	1.225	0.495	0.054	0.999	85	0.011	0.000	1.199	0.638	0.006	98	58	3.4
		Std. Err.	0.067	0.334	0.271	0.163	0.047		0.121	0.001	0.437	0.216	0.039			
		Min	-0.137	0.456	0.006	0.000	0.892		-0.226	-0.003	0.121	0.096	0.000			
		Max	0.148	1.946	0.960	0.933	1.091		0.327	0.003	2.050	0.960	0.388			
	q=3	Mean	-0.008	1.047	0.495	0.058	1.004	86	-0.006	0.000	1.044	0.641	0.004	99	0	NA
		Std. Err.	0.062	0.308	0.271	0.182	0.043		0.112	0.001	0.407	0.213	0.033			
		Min	-0.143	0.377	0.002	0.000	0.908		-0.228	-0.003	0.077	0.104	0.000			
		Max	0.127	1.761	0.964	0.977	1.095		0.273	0.003	1.830	0.966	0.328			
PT	*q=0*	Mean	0.182	3.569	0.979	0.000	0.865	100	0.204	0.000	3.514	0.985	0.000	100	519	98.1
		Std. Err.	0.022	0.112	0.014	0.000	0.017		0.039	0.001	0.130	0.010	0.000			
		Min	0.114	3.331	0.909	0.000	0.832		0.115	-0.002	3.169	0.913	0.000			
		Max	0.224	3.909	0.995	0.000	0.918		0.303	0.001	3.892	0.997	0.000			
	q=1	Mean	0.035	2.363	0.565	0.023	0.975	92	0.067	-0.001	2.285	0.711	0.002	99	119	12.6
		Std. Err.	0.036	0.178	0.238	0.109	0.026		0.074	0.001	0.253	0.180	0.015			
		Min	-0.049	1.883	0.018	0.000	0.904		-0.096	-0.003	1.538	0.134	0.000			
		Max	0.132	2.754	0.902	0.793	1.033		0.305	0.001	2.872	0.946	0.153			
	q=2	Mean	0.031	1.932	0.508	0.027	0.977	92	0.057	0.000	1.867	0.657	0.008	97	124	8.0
		Std. Err.	0.062	0.304	0.244	0.101	0.044		0.115	0.001	0.395	0.199	0.049			
		Min	-0.099	1.118	0.040	0.000	0.855		-0.182	-0.005	0.613	0.093	0.000			
		Max	0.195	2.547	0.898	0.563	1.066		0.430	0.003	2.711	0.954	0.410			
	q=3	Mean	0.021	1.730	0.496	0.040	0.984	91	0.038	0.000	1.686	0.649	0.003	99	122	6.2
		Std. Err.	0.072	0.357	0.244	0.170	0.050		0.130	0.002	0.445	0.196	0.032			
		Min	-0.143	0.826	0.001	0.000	0.849		-0.256	-0.005	0.364	0.104	0.000			
		Max	0.203	2.524	0.920	0.986	1.095		0.426	0.004	2.650	0.956	0.324			
SAL	*q=0*	Mean	0.215	3.571	0.984	0.000	0.839	100	0.215	0.000	3.571	0.988	0.000	100	15218<br>7	373.7
		Std. Err.	0.023	0.107	0.011	0.000	0.018		0.047	0.001	0.140	0.009	0.000			
		Min	0.159	3.360	0.913	0.000	0.796		0.110	-0.002	3.107	0.935	0.000			
		Max	0.267	3.854	0.995	0.000	0.883		0.380	0.002	3.904	0.996	0.000			
		Mean	0.023	2.534	0.567	0.030	0.984	91	0.050	-0.001	2.469	0.709	0.010	96	95	14.1

	q=1	Std. Err.	0.023	0.112	0.253	0.103	0.016		0.044	0.001	0.153	0.203	0.047			
		Min	-0.042	2.274	0.049	0.000	0.946		-0.060	-0.002	2.161	0.108	0.000			
		Max	0.076	2.848	0.932	0.503	1.029		0.147	0.001	2.888	0.967	0.332			
	q=2	Mean	0.020	2.185	0.520	0.062	0.986	87	0.045	0.000	2.124	0.670	0.008	99	93	9.8
		Std. Err.	0.032	0.158	0.260	0.189	0.023		0.056	0.001	0.203	0.197	0.080			
		Min	-0.070	1.764	0.009	0.000	0.927		-0.118	-0.002	1.674	0.048	0.000			
		Max	0.102	2.604	0.945	0.901	1.048		0.177	0.001	2.719	0.956	0.804			
	q=3	Mean	0.019	2.029	0.521	0.048	0.987	89	0.042	0.000	1.973	0.658	0.010	97	93	8.3
		Std. Err.	0.036	0.176	0.253	0.169	0.025		0.060	0.001	0.220	0.206	0.073			
		Min	-0.081	1.548	0.007	0.000	0.919		-0.139	-0.002	1.475	0.065	0.000			
		Max	0.113	2.485	0.962	0.921	1.054		0.186	0.001	2.625	0.963	0.673			
Stool	*q=0*	Mean	0.200	3.292	0.963	0.000	0.851	100	0.241	-0.001	3.185	0.975	0.000	100	346	77.8
		Std. Err.	0.026	0.135	0.026	0.000	0.021		0.056	0.001	0.182	0.019	0.000			
		Min	0.131	2.969	0.856	0.000	0.799		0.098	-0.003	2.853	0.898	0.000			
		Max	0.264	3.654	0.994	0.000	0.905		0.364	0.001	3.637	0.995	0.000			
	q=1	Mean	0.040	1.704	0.526	0.052	0.972	89	0.081	-0.001	1.599	0.705	0.001	99	117	6.7
		Std. Err.	0.048	0.248	0.270	0.176	0.035		0.079	0.001	0.294	0.174	0.013			
		Min	-0.055	0.961	0.007	0.000	0.861		-0.099	-0.003	0.765	0.137	0.000			
		Max	0.187	2.199	0.960	0.922	1.037		0.283	0.001	2.273	0.977	0.123			
	q=2	Mean	0.021	1.298	0.450	0.084	0.985	83	0.052	-0.001	1.218	0.645	0.002	99	99	4.1
		Std. Err.	0.060	0.311	0.290	0.222	0.042		0.097	0.001	0.353	0.199	0.021			
		Min	-0.110	0.427	0.002	0.000	0.857		-0.181	-0.004	0.276	0.118	0.000			
		Max	0.193	1.936	0.964	0.973	1.073		0.287	0.002	2.118	0.976	0.213			
	q=3	Mean	0.010	1.131	0.456	0.055	0.992	83	0.033	0.000	1.072	0.638	0.002	98	85	3.3
		Std. Err.	0.061	0.317	0.281	0.159	0.043		0.098	0.001	0.358	0.204	0.016			
		Min	-0.131	0.290	0.003	0.000	0.871		-0.208	-0.004	0.175	0.130	0.000			
		Max	0.175	1.815	0.965	0.965	1.087		0.268	0.002	2.013	0.974	0.154			
SubP	*q=0*	Mean	0.154	3.653	0.976	0.000	0.887	100	0.184	-0.001	3.580	0.985	0.000	100	347	87.4
		Std. Err.	0.018	0.091	0.017	0.000	0.014		0.033	0.000	0.108	0.011	0.000			
		Min	0.113	3.411	0.882	0.000	0.851		0.104	-0.002	3.287	0.931	0.000			
		Max	0.200	3.868	0.993	0.000	0.919		0.262	0.001	3.857	0.995	0.000			
	q=1	Mean	0.037	2.672	0.704	0.000	0.974	100	0.077	-0.001	2.575	0.820	0.000	100	108	17.4
		Std. Err.	0.019	0.093	0.167	0.001	0.014		0.039	0.000	0.139	0.124	0.000			
		Min	0.005	2.382	0.188	0.000	0.928		0.013	-0.002	2.067	0.341	0.000			
		Max	0.100	2.827	0.954	0.007	0.996		0.227	0.000	2.807	0.985	0.000			
	q=2	Mean	0.049	2.380	0.705	0.000	0.965	100	0.103	-0.001	2.247	0.824	0.000	100	106	13.8
		Std. Err.	0.026	0.125	0.145	0.000	0.019		0.054	0.001	0.189	0.119	0.000			
		Min	0.008	1.920	0.229	0.000	0.892		0.019	-0.003	1.467	0.283	0.000			
		Max	0.147	2.566	0.938	0.001	0.994		0.330	0.000	2.540	0.985	0.000			
	q=3	Mean	0.058	2.233	0.707	0.000	0.959	100	0.119	-0.001	2.081	0.824	0.001	99	108	12.4
		Std. Err.	0.030	0.146	0.145	0.004	0.022		0.061	0.001	0.215	0.125	0.010			
		Min	0.007	1.689	0.147	0.000	0.872		0.010	-0.004	1.193	0.150	0.000			
		Max	0.174	2.455	0.942	0.035	0.995		0.374	0.000	2.448	0.980	0.098			
SupP	*q=0*	Mean	0.165	3.520	0.975	0.000	0.879	100	0.196	-0.001	3.443	0.985	0.000	100	358	81.4
		Std. Err.	0.020	0.105	0.016	0.000	0.016		0.036	0.001	0.113	0.008	0.000			
		Min	0.115	3.288	0.903	0.000	0.843		0.116	-0.002	3.101	0.957	0.000			
		Max	0.210	3.784	0.993	0.000	0.917		0.302	0.001	3.663	0.995	0.000			
	q=1	Mean	0.031	2.591	0.597	0.042	0.978	89	0.063	-0.001	2.514	0.736	0.003	99	114	15.6
		Std. Err.	0.025	0.126	0.279	0.134	0.018		0.046	0.001	0.165	0.217	0.020			
		Min	-0.023	2.245	0.032	0.000	0.930		-0.026	-0.002	2.146	0.125	0.000			
		Max	0.097	2.857	0.958	0.648	1.016		0.166	0.001	2.852	0.973	0.194			
	q=2	Mean	0.039	2.321	0.592	0.041	0.972	93	0.076	-0.001	2.228	0.727	0.002	99	116	12.4
		Std. Err.	0.034	0.168	0.271	0.164	0.024		0.058	0.001	0.213	0.216	0.013			
		Min	-0.036	1.929	0.010	0.000	0.919		-0.039	-0.002	1.779	0.139	0.000			
		Max	0.112	2.685	0.947	0.882	1.025		0.200	0.001	2.640	0.967	0.131			
	q=3	Mean	0.043	2.185	0.585	0.034	0.969	93	0.083	-0.001	2.086	0.720	0.003	99	118	11.0
		Std. Err.	0.038	0.192	0.271	0.133	0.027		0.064	0.001	0.236	0.216	0.028			
		Min	-0.045	1.772	0.018	0.000	0.913		-0.038	-0.002	1.590	0.110	0.000			
		Max	0.120	2.611	0.932	0.789	1.030		0.211	0.001	2.567	0.969	0.281			
TD	*q=0*	Mean	0.160	3.470	0.973	0.000	0.883	100	0.192	-0.001	3.389	0.983	0.000	100	334	74.8
		Std. Err.	0.025	0.126	0.017	0.000	0.020		0.043	0.001	0.156	0.012	0.000			
		Min	0.096	3.204	0.894	0.000	0.840		0.078	-0.002	3.042	0.911	0.000			
		Max	0.214	3.780	0.993	0.000	0.931		0.291	0.001	3.799	0.996	0.000			
	q=1	Mean	0.028	2.426	0.562	0.026	0.980	90	0.061	-0.001	2.346	0.729	0.000	100	106	13.0
		Std. Err.	0.024	0.117	0.258	0.109	0.017		0.046	0.001	0.162	0.177	0.001			
		Min	-0.015	2.078	0.013	0.000	0.932		-0.033	-0.002	1.836	0.214	0.000			
		Max	0.094	2.648	0.947	0.846	1.010		0.220	0.001	2.608	0.964	0.008			
	q=2	Mean	0.031	2.124	0.498	0.068	0.978	84	0.067	-0.001	2.035	0.673	0.005	98	106	9.8
		Std. Err.	0.037	0.181	0.273	0.190	0.026		0.068	0.001	0.240	0.201	0.038			
		Min	-0.034	1.540	0.007	0.000	0.895		-0.066	-0.003	1.177	0.098	0.000			
		Max	0.144	2.452	0.943	0.915	1.023		0.313	0.002	2.395	0.958	0.364			
	q=3	Mean	0.027	1.978	0.480	0.053	0.981	90	0.061	-0.001	1.892	0.643	0.004	98	101	8.3
		Std. Err.	0.045	0.223	0.250	0.181	0.032		0.081	0.001	0.283	0.204	0.030			
		Min	-0.054	1.264	0.001	0.000	0.879		-0.094	-0.004	0.888	0.113	0.000			
		Max	0.165	2.389	0.926	0.988	1.037		0.354	0.002	2.298	0.949	0.260			

Th	*q=0*	Mean	0.242	3.560	0.985	0.000	0.817	100	0.239	0.000	3.568	0.989	0.000	100	0	NA
		Std. Err.	0.026	0.123	0.009	0.000	0.021		0.053	0.001	0.159	0.006	0.000			
		Min	0.173	3.278	0.941	0.000	0.764		0.083	-0.002	3.212	0.963	0.000			
		Max	0.306	3.895	0.997	0.000	0.873		0.359	0.003	3.966	0.998	0.000			
	q=1	Mean	0.032	2.429	0.509	0.060	0.977	89	0.070	-0.001	2.335	0.691	0.001	99	100	13.3
		Std. Err.	0.034	0.162	0.270	0.196	0.024		0.070	0.001	0.236	0.193	0.007			
		Min	-0.036	1.865	0.006	0.000	0.894		-0.097	-0.003	1.593	0.163	0.000			
		Max	0.146	2.741	0.908	0.935	1.024		0.273	0.002	2.801	0.977	0.068			
	q=2	Mean	0.019	1.998	0.446	0.052	0.986	88	0.054	-0.001	1.912	0.612	0.007	97	84	8.2
		Std. Err.	0.056	0.275	0.251	0.152	0.040		0.104	0.001	0.361	0.212	0.046			
		Min	-0.104	1.150	0.022	0.000	0.860		-0.202	-0.004	0.800	0.102	0.000			
		Max	0.189	2.600	0.925	0.756	1.070		0.339	0.003	2.601	0.988	0.351			
	q=3	Mean	0.007	1.798	0.454	0.049	0.994	85	0.036	-0.001	1.728	0.615	0.012	97	69	6.3
		Std. Err.	0.066	0.325	0.256	0.138	0.047		0.117	0.001	0.407	0.205	0.081			
		Min	-0.143	0.878	0.026	0.000	0.859		-0.256	-0.004	0.552	0.058	0.000			
		Max	0.190	2.538	0.930	0.711	1.094		0.337	0.004	2.541	0.986	0.711			

* The model parameters were computed from 100 times of repeated DAR modeling from 100 times of re-sampling; *N* is the number of successful DAR model-fitting out of 100 times of re-sampling.

Table S6. The intra-DAR (intra-individual Diversity-Area Relationship) models

Taxon	Diversity Order	Power Law (PL)						PL with Exponential Cutoff (PLEC)								RIP
		z	*c*	*R*	*p*-value	*g*	**N*	*z*	*d*	*c*	*R*	*p*-value	A_{max}	D_{max}	**N*	
Genus	*q=0*	0.417	45.468	0.962	0.000	0.665	1000	0.555	-0.030	44.168	0.974	0.000	41	140.0	454	32.5
	q=1	0.512	5.228	0.900	0.003	0.574	933	0.439	0.013	5.490	0.929	0.004	22	18.2	584	28.7
	q=2	0.535	2.583	0.886	0.004	0.551	865	0.273	0.051	2.915	0.920	0.005	29	12.4	564	20.8
	q=3	0.493	2.094	0.880	0.004	0.593	838	0.172	0.063	2.401	0.915	0.006	15	5.8	565	36.1
Family	*q=0*	0.261	31.785	0.959	0.000	0.802	1000	0.391	-0.030	30.938	0.975	0.000	34	60.6	604	52.5
	q=1	0.472	4.740	0.906	0.002	0.613	923	0.467	-0.004	4.988	0.935	0.003	22	14.3	580	33.1
	q=2	0.526	2.581	0.892	0.003	0.560	885	0.338	0.034	2.954	0.925	0.004	28	9.5	542	27.2
	q=3	0.498	2.119	0.886	0.004	0.588	859	0.226	0.050	2.497	0.918	0.005	21	6.7	530	31.6
Order	*q=0*	0.292	16.395	0.954	0.000	0.776	1000	0.425	-0.032	16.248	0.973	0.000	53	34.5	596	47.5
	q=1	0.347	3.869	0.892	0.003	0.728	880	0.461	-0.030	3.912	0.927	0.005	23	8.9	604	43.5
	q=2	0.422	2.430	0.888	0.003	0.660	850	0.393	-0.001	2.588	0.920	0.005	22	6.4	576	38.0
	q=3	0.427	2.040	0.887	0.003	0.656	839	0.303	0.020	2.219	0.919	0.005	17	5.3	547	38.5
Class	*q=0*	0.222	12.756	0.935	0.000	0.834	999	0.335	-0.028	12.591	0.962	0.001	26	21.6	660	59.1
	q=1	0.383	3.133	0.892	0.003	0.696	918	0.492	-0.027	3.133	0.932	0.004	25	7.8	614	40.2
	q=2	0.455	2.022	0.891	0.003	0.629	894	0.416	0.003	2.153	0.924	0.005	27	6.0	542	33.7
	q=3	0.451	1.738	0.888	0.004	0.633	891	0.322	0.023	1.895	0.923	0.005	24	4.9	514	35.5
Phylum	*q=0*	NA	NA	NA	NA	NA	NA	NA	NA	NA	NA	NA	NA	NA	NA	NA
	q=1	0.285	2.366	0.880	0.003	0.782	900	0.380	-0.025	2.382	0.916	0.005	18	4.5	617	52.6
	q=2	0.319	1.749	0.875	0.004	0.753	877	0.399	-0.024	1.820	0.909	0.006	49	3.8	579	46.0
	q=3	0.311	1.576	0.871	0.004	0.759	866	0.374	-0.020	1.647	0.903	0.007	25	3.3	561	47.8

* The model parameters were computed from 1000 times of repeated DAR modeling based on 1000 times of re-sampling; *N* is the number of successful DAR model-fitting out of 1000 times of re-sampling.

Table S7. The permutation test for the differences in the DAR parameters between the digestive sites *via* 1000 times of re-sampling, at *genus* taxon level, for each diversity order (q=0-3).

Diversity Order	DAR	Treatments	Parameter	Former	Latter	Delta Δ`=|H-D|	Permutated Mean |Δ|	SD of |Δ|	p-value
q=0	PL	BM vs. HP	z	0.289	0.305	0.016	0.035	0.026	0.702
			ln(c)	4.078	4.151	0.073	0.168	0.125	0.729
		BM vs. KG	z	0.289	0.339	0.050	0.044	0.033	0.377
			ln(c)	4.078	3.627	0.451	0.219	0.159	0.099
		BM vs. PT	z	0.289	0.293	0.004	0.034	0.025	0.910
			ln(c)	4.078	4.023	0.056	0.169	0.126	0.786
		BM vs. Sal	z	0.289	0.297	0.008	0.033	0.026	0.852
			ln(c)	4.078	4.109	0.030	0.160	0.125	0.880
		BM vs. Stool	z	0.289	0.249	0.040	0.034	0.025	0.355
			ln(c)	4.078	4.036	0.042	0.162	0.123	0.818
		BM vs. SubP	z	0.289	0.280	0.009	0.032	0.024	0.838
			ln(c)	4.078	4.042	0.036	0.155	0.116	0.841
		BM vs. SupP	z	0.289	0.277	0.012	0.035	0.027	0.786
			ln(c)	4.078	3.898	0.181	0.174	0.133	0.391
		BM vs. TD	z	0.289	0.267	0.022	0.037	0.028	0.616
			ln(c)	4.078	3.841	0.237	0.182	0.142	0.303
		BM vs. Th	z	0.289	0.322	0.033	0.040	0.030	0.508
			ln(c)	4.078	4.145	0.066	0.191	0.142	0.793
		HP vs. KG	z	0.305	0.339	0.033	0.044	0.033	0.547
			ln(c)	4.151	3.627	0.524	0.212	0.163	0.045
		HP vs. PT	z	0.305	0.293	0.012	0.035	0.025	0.801
			ln(c)	4.151	4.023	0.129	0.170	0.122	0.566
		HP vs. Sal	z	0.305	0.297	0.009	0.033	0.024	0.845
			ln(c)	4.151	4.109	0.043	0.154	0.116	0.818
		HP vs. Stool	z	0.305	0.249	0.056	0.032	0.024	0.157
			ln(c)	4.151	4.036	0.116	0.153	0.114	0.554
		HP vs. SubP	z	0.305	0.280	0.025	0.032	0.024	0.521
			ln(c)	4.151	4.042	0.109	0.152	0.114	0.561
		HP vs. SupP	z	0.305	0.277	0.028	0.035	0.026	0.515
			ln(c)	4.151	3.898	0.254	0.166	0.126	0.219
		HP vs. TD	z	0.305	0.267	0.039	0.039	0.029	0.437
			ln(c)	4.151	3.841	0.310	0.187	0.136	0.198
		HP vs. Th	z	0.305	0.322	0.016	0.037	0.028	0.729
			ln(c)	4.151	4.145	0.007	0.178	0.139	0.967
		KG vs. PT	z	0.339	0.293	0.046	0.046	0.034	0.431
			ln(c)	3.627	4.023	0.395	0.226	0.169	0.164
		KG vs. Sal	z	0.339	0.297	0.042	0.046	0.034	0.462
			ln(c)	3.627	4.109	0.481	0.218	0.158	0.076
		KG vs. Stool	z	0.339	0.249	0.090	0.037	0.027	0.056
			ln(c)	3.627	4.036	0.408	0.181	0.132	0.065
		KG vs. SubP	z	0.339	0.280	0.059	0.038	0.030	0.219
			ln(c)	3.627	4.042	0.415	0.184	0.144	0.083
		KG vs. SupP	z	0.339	0.277	0.062	0.038	0.029	0.192
			ln(c)	3.627	3.898	0.270	0.187	0.144	0.235
		KG vs. TD	z	0.339	0.267	0.072	0.041	0.031	0.167
			ln(c)	3.627	3.841	0.214	0.204	0.153	0.398
		KG vs. Th	z	0.339	0.322	0.017	0.049	0.037	0.768
			ln(c)	3.627	4.145	0.517	0.237	0.178	0.088
		PT vs. Sal	z	0.293	0.297	0.004	0.035	0.025	0.937
			ln(c)	4.023	4.109	0.086	0.170	0.123	0.704
		PT vs. Stool	z	0.293	0.249	0.044	0.034	0.025	0.300
			ln(c)	4.023	4.036	0.013	0.159	0.122	0.949
		PT vs. SubP	z	0.293	0.280	0.013	0.030	0.024	0.712
			ln(c)	4.023	4.042	0.019	0.149	0.115	0.919
		PT vs. SupP	z	0.293	0.277	0.016	0.034	0.026	0.696
			ln(c)	4.023	3.898	0.125	0.169	0.129	0.537
		PT vs. TD	z	0.293	0.267	0.027	0.038	0.028	0.584
			ln(c)	4.023	3.841	0.182	0.189	0.140	0.463
		PT vs. Th	z	0.293	0.322	0.028	0.039	0.030	0.568
			ln(c)	4.023	4.145	0.122	0.191	0.141	0.629
		Sal vs. Stool	z	0.297	0.249	0.048	0.032	0.025	0.230
			ln(c)	4.109	4.036	0.073	0.149	0.114	0.682
		Sal vs. SubP	z	0.297	0.280	0.017	0.030	0.023	0.659
			ln(c)	4.109	4.042	0.067	0.142	0.110	0.695
		Sal vs. SupP	z	0.297	0.277	0.020	0.033	0.026	0.618
			ln(c)	4.109	3.898	0.211	0.164	0.126	0.302

		Sal vs. TD	z	0.297	0.267	0.030	0.040	0.030	0.548
			ln(c)	4.109	3.841	0.267	0.189	0.147	0.257
		Sal vs. Th	z	0.297	0.322	0.025	0.036	0.028	0.570
			ln(c)	4.109	4.145	0.036	0.171	0.130	0.869
		Stool vs. SubP	z	0.249	0.280	0.031	0.030	0.023	0.395
			ln(c)	4.036	4.042	0.006	0.140	0.110	0.966
		Stool vs. SupP	z	0.249	0.277	0.028	0.029	0.022	0.442
			ln(c)	4.036	3.898	0.138	0.135	0.101	0.409
		Stool vs. TD	z	0.249	0.267	0.017	0.031	0.023	0.659
			ln(c)	4.036	3.841	0.195	0.150	0.111	0.299
		Stool vs. Th	z	0.249	0.322	0.073	0.035	0.027	0.106
			ln(c)	4.036	4.145	0.109	0.171	0.131	0.600
		SubP vs. SupP	z	0.280	0.277	0.003	0.030	0.023	0.922
			ln(c)	4.042	3.898	0.144	0.144	0.107	0.421
		SubP vs. TD	z	0.280	0.267	0.014	0.030	0.023	0.720
			ln(c)	4.042	3.841	0.201	0.151	0.116	0.284
		SubP vs. Th	z	0.280	0.322	0.041	0.037	0.027	0.384
			ln(c)	4.042	4.145	0.103	0.176	0.130	0.659
		SupP vs. TD	z	0.277	0.267	0.011	0.033	0.025	0.803
			ln(c)	3.898	3.841	0.056	0.158	0.123	0.763
		SupP vs. Th	z	0.277	0.322	0.045	0.042	0.031	0.393
			ln(c)	3.898	4.145	0.247	0.201	0.150	0.333
		TD vs. Th	z	0.267	0.322	0.055	0.045	0.034	0.333
			ln(c)	3.841	4.145	0.303	0.219	0.168	0.265
	PLEC	BM vs. HP	z	0.346	0.351	0.005	0.057	0.045	0.941
			ln(c)	3.938	4.040	0.102	0.207	0.157	0.691
			D_{max}	272.024	334.120	62.095	56.903	92.558	0.263
		BM vs. KG	z	0.346	0.398	0.052	0.073	0.054	0.574
			ln(c)	3.938	3.481	0.457	0.271	0.197	0.183
			D_{max}	272.024	231.199	40.826	48.763	97.862	0.332
		BM vs. PT	z	0.346	0.329	0.017	0.055	0.041	0.802
			ln(c)	3.938	3.934	0.004	0.199	0.152	0.981
			D_{max}	272.024	287.670	15.646	43.647	82.850	0.610
		BM vs. Sal	z	0.346	0.323	0.022	0.054	0.043	0.729
			ln(c)	3.938	4.045	0.107	0.193	0.149	0.658
			D_{max}	272.024	333.093	61.069	55.935	79.440	0.259
		BM vs. Stool	z	0.346	0.304	0.041	0.064	0.047	0.615
			ln(c)	3.938	3.895	0.043	0.223	0.164	0.886
			D_{max}	272.024	211.829	60.196	27.543	50.230	0.079
		BM vs. SubP	z	0.346	0.305	0.040	0.053	0.040	0.543
			ln(c)	3.938	3.981	0.043	0.189	0.144	0.854
			D_{max}	272.024	289.235	17.210	51.694	84.419	0.647
		BM vs. SupP	z	0.346	0.284	0.061	0.054	0.041	0.365
			ln(c)	3.938	3.880	0.058	0.202	0.153	0.832
			D_{max}	272.024	328.890	56.866	49.004	96.661	0.233
		BM vs. TD	z	0.346	0.290	0.056	0.055	0.043	0.415
			ln(c)	3.938	3.784	0.154	0.204	0.159	0.516
			D_{max}	272.024	220.848	51.176	49.173	73.049	0.269
		BM vs. Th	z	0.346	0.356	0.010	0.060	0.047	0.889
			ln(c)	3.938	4.060	0.122	0.218	0.163	0.676
			D_{max}	272.024	387.491	115.466	82.657	169.175	0.173
		HP vs. KG	z	0.351	0.398	0.047	0.075	0.056	0.629
			ln(c)	4.040	3.481	0.559	0.270	0.207	0.102
			D_{max}	334.120	231.199	102.921	62.304	104.493	0.144
		HP vs. PT	z	0.351	0.329	0.022	0.055	0.038	0.778
			ln(c)	4.040	3.934	0.106	0.195	0.145	0.668
			D_{max}	334.120	287.670	46.450	64.032	123.404	0.399
		HP vs. Sal	z	0.351	0.323	0.028	0.050	0.038	0.660
			ln(c)	4.040	4.045	0.005	0.177	0.133	0.980
			D_{max}	334.120	333.093	1.027	85.405	282.206	0.988
		HP vs. Stool	z	0.351	0.304	0.047	0.059	0.047	0.532
			ln(c)	4.040	3.895	0.145	0.206	0.160	0.568
			D_{max}	334.120	211.829	122.291	43.535	69.657	0.058
		HP vs. SubP	z	0.351	0.305	0.046	0.049	0.036	0.468
			ln(c)	4.040	3.981	0.059	0.174	0.131	0.792
			D_{max}	334.120	289.235	44.885	74.822	149.526	0.432
		HP vs. SupP	z	0.351	0.284	0.067	0.053	0.040	0.321
			ln(c)	4.040	3.880	0.160	0.192	0.146	0.513
			D_{max}	334.120	328.890	5.230	58.312	104.450	0.882
		HP vs. TD	z	0.351	0.290	0.061	0.059	0.044	0.419
			ln(c)	4.040	3.784	0.256	0.222	0.162	0.364

			D_{max}	334.120	220.848	113.272	69.203	119.671	0.152
		HP vs. Th	z	0.351	0.356	0.005	0.056	0.043	0.928
			$\ln(c)$	4.040	4.060	0.020	0.204	0.158	0.936
			D_{max}	334.120	387.491	53.371	72.526	105.341	0.389
		KG vs. PT	z	0.398	0.329	0.069	0.072	0.054	0.433
			$\ln(c)$	3.481	3.934	0.453	0.269	0.201	0.176
			D_{max}	231.199	287.670	56.471	59.717	93.979	0.289
		KG vs. Sal	z	0.398	0.323	0.075	0.081	0.060	0.465
			$\ln(c)$	3.481	4.045	0.564	0.286	0.212	0.110
			D_{max}	231.199	333.093	101.894	67.020	101.453	0.176
		KG vs. Stool	z	0.398	0.304	0.094	0.071	0.053	0.303
			$\ln(c)$	3.481	3.895	0.414	0.249	0.183	0.174
			D_{max}	231.199	211.829	19.370	27.473	42.507	0.420
		KG vs. SubP	z	0.398	0.305	0.093	0.070	0.053	0.292
			$\ln(c)$	3.481	3.981	0.499	0.247	0.190	0.116
			D_{max}	231.199	289.235	58.036	69.814	150.699	0.292
		KG vs. SupP	z	0.398	0.284	0.114	0.063	0.047	0.136
			$\ln(c)$	3.481	3.880	0.399	0.231	0.175	0.167
			D_{max}	231.199	328.890	97.692	57.175	83.386	0.152
		KG vs. TD	z	0.398	0.290	0.108	0.059	0.047	0.145
			$\ln(c)$	3.481	3.784	0.302	0.226	0.170	0.283
			D_{max}	231.199	220.848	10.350	58.609	86.211	0.785
		KG vs. Th	z	0.398	0.356	0.042	0.080	0.059	0.688
			$\ln(c)$	3.481	4.060	0.579	0.287	0.216	0.101
			D_{max}	231.199	387.491	156.292	77.538	127.394	0.117
		PT vs. Sal	z	0.329	0.323	0.006	0.051	0.038	0.927
			$\ln(c)$	3.934	4.045	0.111	0.189	0.139	0.653
			D_{max}	287.670	333.093	45.423	70.043	108.533	0.438
		PT vs. Stool	z	0.329	0.304	0.025	0.063	0.047	0.763
			$\ln(c)$	3.934	3.895	0.039	0.216	0.161	0.888
			D_{max}	287.670	211.829	75.841	36.536	73.158	0.096
		PT vs. SubP	z	0.329	0.305	0.024	0.049	0.035	0.705
			$\ln(c)$	3.934	3.981	0.047	0.177	0.128	0.851
			D_{max}	287.670	289.235	1.565	65.851	99.981	0.975
		PT vs. SupP	z	0.329	0.284	0.045	0.048	0.037	0.465
			$\ln(c)$	3.934	3.880	0.054	0.182	0.138	0.805
			D_{max}	287.670	328.890	41.220	69.550	193.876	0.409
		PT vs. TD	z	0.329	0.290	0.039	0.055	0.040	0.567
			$\ln(c)$	3.934	3.784	0.150	0.207	0.157	0.571
			D_{max}	287.670	220.848	66.822	66.204	100.578	0.296
		PT vs. Th	z	0.329	0.356	0.027	0.052	0.042	0.648
			$\ln(c)$	3.934	4.060	0.126	0.200	0.155	0.612
			D_{max}	287.670	387.491	99.821	83.178	132.081	0.215
		Sal vs. Stool	z	0.323	0.304	0.019	0.061	0.050	0.771
			$\ln(c)$	4.045	3.895	0.150	0.206	0.165	0.539
			D_{max}	333.093	211.829	121.264	37.291	48.416	0.045
		Sal vs. SubP	z	0.323	0.305	0.018	0.047	0.035	0.773
			$\ln(c)$	4.045	3.981	0.065	0.169	0.125	0.771
			D_{max}	333.093	289.235	43.858	76.788	134.967	0.442
		Sal vs. SupP	z	0.323	0.284	0.039	0.049	0.037	0.513
			$\ln(c)$	4.045	3.880	0.165	0.188	0.138	0.492
			D_{max}	333.093	328.890	4.203	75.160	152.312	0.935
		Sal vs. TD	z	0.323	0.290	0.034	0.055	0.043	0.620
			$\ln(c)$	4.045	3.784	0.262	0.213	0.160	0.320
			D_{max}	333.093	220.848	112.245	76.140	115.038	0.178
		Sal vs. Th	z	0.323	0.356	0.033	0.054	0.042	0.627
			$\ln(c)$	4.045	4.060	0.015	0.191	0.149	0.944
			D_{max}	333.093	387.491	54.397	107.725	363.442	0.469
		Stool vs. SubP	z	0.304	0.305	0.001	0.057	0.045	0.990
			$\ln(c)$	3.895	3.981	0.085	0.195	0.155	0.721
			D_{max}	211.829	289.235	77.406	29.796	60.633	0.067
		Stool vs. SupP	z	0.304	0.284	0.020	0.059	0.044	0.787
			$\ln(c)$	3.895	3.880	0.015	0.199	0.145	0.966
			D_{max}	211.829	328.890	117.061	23.255	32.072	0.027
		Stool vs. TD	z	0.304	0.290	0.015	0.058	0.042	0.838
			$\ln(c)$	3.895	3.784	0.112	0.201	0.145	0.680
			D_{max}	211.829	220.848	9.019	22.577	44.686	0.635
		Stool vs. Th	z	0.304	0.356	0.052	0.062	0.047	0.483
			$\ln(c)$	3.895	4.060	0.165	0.216	0.169	0.538
			D_{max}	211.829	387.491	175.662	55.225	86.363	0.053
		SubP vs. SupP	z	0.305	0.284	0.021	0.042	0.032	0.690

			ln(*c*)	3.981	3.880	0.100	0.159	0.121	0.604
			D_{max}	289.235	328.890	39.656	67.822	89.642	0.476
		SubP vs. TD	*z*	0.305	0.290	0.016	0.045	0.033	0.784
			ln(*c*)	3.981	3.784	0.197	0.166	0.126	0.339
			D_{max}	289.235	220.848	68.386	62.949	90.608	0.271
		SubP vs. Th	*z*	0.305	0.356	0.051	0.054	0.039	0.452
			ln(*c*)	3.981	4.060	0.079	0.191	0.143	0.736
			D_{max}	289.235	387.491	98.256	86.650	127.830	0.250
		SupP vs. TD	*z*	0.284	0.290	0.005	0.046	0.034	0.921
			ln(*c*)	3.880	3.784	0.097	0.168	0.124	0.657
			D_{max}	328.890	220.848	108.042	56.298	77.621	0.141
		SupP vs. Th	*z*	0.284	0.356	0.072	0.058	0.044	0.318
			ln(*c*)	3.880	4.060	0.180	0.214	0.169	0.476
			D_{max}	328.890	387.491	58.600	84.323	134.921	0.385
		TD vs. Th	*z*	0.290	0.356	0.066	0.064	0.046	0.416
			ln(*c*)	3.784	4.060	0.276	0.243	0.181	0.371
			D_{max}	220.848	387.491	166.642	112.077	242.099	0.153
q=1	PL	BM vs. HP	*z*	0.020	0.025	0.005	0.070	0.051	0.965
			ln(*c*)	1.937	2.229	0.292	0.351	0.251	0.509
		BM vs. KG	*z*	0.020	0.041	0.021	0.071	0.057	0.796
			ln(*c*)	1.937	1.774	0.163	0.358	0.281	0.690
		BM vs. PT	*z*	0.020	0.053	0.033	0.077	0.055	0.736
			ln(*c*)	1.937	2.505	0.568	0.382	0.276	0.242
		BM vs. Sal	*z*	0.020	0.033	0.012	0.081	0.063	0.877
			ln(*c*)	1.937	2.795	0.858	0.408	0.311	0.095
		BM vs. Stool	*z*	0.020	0.045	0.025	0.070	0.052	0.776
			ln(*c*)	1.937	2.084	0.147	0.349	0.260	0.745
		BM vs. SubP	*z*	0.020	0.042	0.022	0.076	0.056	0.818
			ln(*c*)	1.937	2.851	0.914	0.381	0.283	0.055
		BM vs. SupP	*z*	0.020	0.035	0.015	0.066	0.052	0.838
			ln(*c*)	1.937	2.745	0.808	0.338	0.259	0.060
		BM vs. TD	*z*	0.020	0.036	0.016	0.063	0.048	0.841
			ln(*c*)	1.937	2.552	0.615	0.323	0.246	0.131
		BM vs. Th	*z*	0.020	0.044	0.024	0.070	0.052	0.768
			ln(*c*)	1.937	2.600	0.663	0.349	0.262	0.125
		HP vs. KG	*z*	0.025	0.041	0.016	0.078	0.060	0.854
			ln(*c*)	2.229	1.774	0.455	0.390	0.294	0.362
		HP vs. PT	*z*	0.025	0.053	0.028	0.065	0.048	0.719
			ln(*c*)	2.229	2.505	0.276	0.325	0.243	0.503
		HP vs. Sal	*z*	0.025	0.033	0.008	0.062	0.047	0.918
			ln(*c*)	2.229	2.795	0.567	0.311	0.235	0.133
		HP vs. Stool	*z*	0.025	0.045	0.020	0.067	0.051	0.823
			ln(*c*)	2.229	2.084	0.144	0.340	0.262	0.727
		HP vs. SubP	*z*	0.025	0.042	0.017	0.060	0.046	0.831
			ln(*c*)	2.229	2.851	0.623	0.300	0.232	0.101
		HP vs. SupP	*z*	0.025	0.035	0.010	0.056	0.043	0.875
			ln(*c*)	2.229	2.745	0.516	0.282	0.222	0.152
		HP vs. TD	*z*	0.025	0.036	0.011	0.053	0.040	0.864
			ln(*c*)	2.229	2.552	0.323	0.263	0.198	0.333
		HP vs. Th	*z*	0.025	0.044	0.019	0.061	0.045	0.815
			ln(*c*)	2.229	2.600	0.372	0.310	0.225	0.349
		KG vs. PT	*z*	0.041	0.053	0.012	0.087	0.065	0.898
			ln(*c*)	1.774	2.505	0.731	0.440	0.327	0.195
		KG vs. Sal	*z*	0.041	0.033	0.008	0.091	0.066	0.937
			ln(*c*)	1.774	2.795	1.021	0.449	0.326	0.061
		KG vs. Stool	*z*	0.041	0.045	0.004	0.073	0.059	0.969
			ln(*c*)	1.774	2.084	0.310	0.367	0.301	0.495
		KG vs. SubP	*z*	0.041	0.042	0.001	0.083	0.065	0.995
			ln(*c*)	1.774	2.851	1.077	0.418	0.320	0.038
		KG vs. SupP	*z*	0.041	0.035	0.006	0.075	0.061	0.934
			ln(*c*)	1.774	2.745	0.971	0.385	0.300	0.049
		KG vs. TD	*z*	0.041	0.036	0.005	0.072	0.053	0.940
			ln(*c*)	1.774	2.552	0.778	0.359	0.262	0.083
		KG vs. Th	*z*	0.041	0.044	0.003	0.084	0.062	0.971
			ln(*c*)	1.774	2.600	0.826	0.428	0.318	0.116
		PT vs. Sal	*z*	0.053	0.033	0.021	0.044	0.038	0.679
			ln(*c*)	2.505	2.795	0.290	0.224	0.185	0.298
		PT vs. Stool	*z*	0.053	0.045	0.008	0.071	0.058	0.928
			ln(*c*)	2.505	2.084	0.420	0.360	0.293	0.339
		PT vs. SubP	*z*	0.053	0.042	0.011	0.039	0.033	0.803
			ln(*c*)	2.505	2.851	0.347	0.195	0.166	0.177

		PT vs. SupP	z	0.053	0.035	0.018	0.040	0.032	0.725
			ln(c)	2.505	2.745	0.240	0.203	0.158	0.324
		PT vs. TD	z	0.053	0.036	0.018	0.040	0.034	0.698
			ln(c)	2.505	2.552	0.047	0.201	0.167	0.854
		PT vs. Th	z	0.053	0.044	0.009	0.047	0.039	0.852
			ln(c)	2.505	2.600	0.095	0.234	0.194	0.718
		Sal vs. Stool	z	0.033	0.045	0.012	0.084	0.065	0.918
			ln(c)	2.795	2.084	0.711	0.430	0.331	0.176
		Sal vs. SubP	z	0.033	0.042	0.009	0.025	0.020	0.762
			ln(c)	2.795	2.851	0.056	0.125	0.097	0.708
		Sal vs. SupP	z	0.033	0.035	0.003	0.030	0.023	0.944
			ln(c)	2.795	2.745	0.050	0.148	0.114	0.787
		Sal vs. TD	z	0.033	0.036	0.003	0.033	0.026	0.934
			ln(c)	2.795	2.552	0.243	0.164	0.126	0.239
		Sal vs. Th	z	0.033	0.044	0.011	0.039	0.032	0.807
			ln(c)	2.795	2.600	0.195	0.195	0.153	0.420
		Stool vs. SubP	z	0.045	0.042	0.003	0.079	0.065	0.972
			ln(c)	2.084	2.851	0.767	0.400	0.328	0.143
		Stool vs. SupP	z	0.045	0.035	0.009	0.071	0.059	0.902
			ln(c)	2.084	2.745	0.661	0.359	0.304	0.142
		Stool vs. TD	z	0.045	0.036	0.009	0.066	0.052	0.927
			ln(c)	2.084	2.552	0.467	0.333	0.274	0.255
		Stool vs. Th	z	0.045	0.044	0.001	0.070	0.057	0.989
			ln(c)	2.084	2.600	0.516	0.356	0.301	0.243
		SubP vs. SupP	z	0.042	0.035	0.006	0.028	0.022	0.874
			ln(c)	2.851	2.745	0.106	0.141	0.110	0.549
		SubP vs. TD	z	0.042	0.036	0.006	0.032	0.026	0.889
			ln(c)	2.851	2.552	0.300	0.160	0.128	0.157
		SubP vs. Th	z	0.042	0.044	0.002	0.034	0.028	0.952
			ln(c)	2.851	2.600	0.251	0.168	0.139	0.238
		SupP vs. TD	z	0.035	0.036	0.000	0.032	0.025	0.992
			ln(c)	2.745	2.552	0.193	0.157	0.124	0.318
		SupP vs. Th	z	0.035	0.044	0.009	0.037	0.029	0.844
			ln(c)	2.745	2.600	0.145	0.185	0.147	0.514
		TD vs. Th	z	0.036	0.044	0.008	0.034	0.027	0.825
			ln(c)	2.552	2.600	0.049	0.174	0.136	0.821
	PLEC	BM vs. HP	z	0.043	0.046	0.004	0.130	0.096	0.986
			ln(c)	1.886	2.181	0.295	0.449	0.333	0.601
			D_{max}	7.670	10.500	2.829	0.908	0.711	0.017
		BM vs. KG	z	0.043	0.073	0.030	0.133	0.101	0.851
			ln(c)	1.886	1.705	0.181	0.463	0.356	0.752
			D_{max}	7.670	7.222	0.448	0.883	0.669	0.702
		BM vs. PT	z	0.043	0.082	0.039	0.143	0.101	0.838
			ln(c)	1.886	2.438	0.552	0.491	0.350	0.386
			D_{max}	7.670	15.927	8.256	1.498	1.132	0.000
		BM vs. Sal	z	0.043	0.058	0.015	0.145	0.104	0.938
			ln(c)	1.886	2.741	0.855	0.498	0.373	0.185
			D_{max}	7.670	19.168	11.498	1.484	1.154	0.000
		BM vs. Stool	z	0.043	0.080	0.038	0.126	0.098	0.793
			ln(c)	1.886	2.004	0.117	0.459	0.345	0.846
			D_{max}	7.670	10.080	2.409	1.545	1.361	0.202
		BM vs. SubP	z	0.043	0.085	0.042	0.133	0.096	0.816
			ln(c)	1.886	2.744	0.858	0.474	0.344	0.150
			D_{max}	7.670	21.296	13.625	1.623	1.240	0.000
		BM vs. SupP	z	0.043	0.066	0.023	0.124	0.095	0.880
			ln(c)	1.886	2.673	0.787	0.437	0.340	0.155
			D_{max}	7.670	18.553	10.883	1.472	1.216	0.001
		BM vs. TD	z	0.043	0.069	0.027	0.118	0.088	0.860
			ln(c)	1.886	2.471	0.585	0.419	0.315	0.251
			D_{max}	7.670	15.322	7.652	1.129	0.942	0.001
		BM vs. Th	z	0.043	0.082	0.039	0.128	0.091	0.825
			ln(c)	1.886	2.516	0.630	0.439	0.321	0.273
			D_{max}	7.670	16.736	9.066	1.243	0.978	0.000
		HP vs. KG	z	0.046	0.073	0.027	0.141	0.106	0.872
			ln(c)	2.181	1.705	0.477	0.498	0.367	0.438
			D_{max}	10.500	7.222	3.278	1.024	0.827	0.023
		HP vs. PT	z	0.046	0.082	0.036	0.114	0.087	0.790
			ln(c)	2.181	2.438	0.257	0.403	0.313	0.596
			D_{max}	10.500	15.927	5.427	1.487	1.188	0.007
		HP vs. Sal	z	0.046	0.058	0.011	0.119	0.090	0.945
			ln(c)	2.181	2.741	0.560	0.406	0.316	0.270

			D_{max}	10.500	19.168	8.668	1.451	1.141	0.000
		HP vs. Stool	z	0.046	0.080	0.034	0.124	0.096	0.832
			ln(c)	2.181	2.004	0.178	0.458	0.358	0.747
			D_{max}	10.500	10.080	0.420	1.640	1.346	0.835
		HP vs. SubP	z	0.046	0.085	0.039	0.113	0.088	0.789
			ln(c)	2.181	2.744	0.563	0.400	0.314	0.259
			D_{max}	10.500	21.296	10.796	1.456	1.235	0.001
		HP vs. SupP	z	0.046	0.066	0.020	0.105	0.080	0.865
			ln(c)	2.181	2.673	0.492	0.370	0.284	0.273
			D_{max}	10.500	18.553	8.053	1.363	1.135	0.000
		HP vs. TD	z	0.046	0.069	0.023	0.100	0.078	0.857
			ln(c)	2.181	2.471	0.290	0.355	0.278	0.507
			D_{max}	10.500	15.322	4.822	0.986	0.818	0.004
		HP vs. Th	z	0.046	0.082	0.036	0.113	0.082	0.799
			ln(c)	2.181	2.516	0.335	0.387	0.289	0.485
			D_{max}	10.500	16.736	6.236	1.324	1.182	0.006
		KG vs. PT	z	0.073	0.082	0.009	0.159	0.120	0.961
			ln(c)	1.705	2.438	0.734	0.567	0.431	0.313
			D_{max}	7.222	15.927	8.705	1.482	1.132	0.000
		KG vs. Sal	z	0.073	0.058	0.015	0.177	0.130	0.939
			ln(c)	1.705	2.741	1.036	0.608	0.446	0.181
			D_{max}	7.222	19.168	11.946	1.616	1.296	0.000
		KG vs. Stool	z	0.073	0.080	0.007	0.133	0.102	0.965
			ln(c)	1.705	2.004	0.299	0.496	0.379	0.620
			D_{max}	7.222	10.080	2.858	1.671	1.306	0.157
		KG vs. SubP	z	0.073	0.085	0.012	0.155	0.114	0.942
			ln(c)	1.705	2.744	1.040	0.544	0.416	0.139
			D_{max}	7.222	21.296	14.074	1.761	1.423	0.000
		KG vs. SupP	z	0.073	0.066	0.007	0.141	0.104	0.966
			ln(c)	1.705	2.673	0.968	0.505	0.385	0.127
			D_{max}	7.222	18.553	11.331	1.569	1.227	0.000
		KG vs. TD	z	0.073	0.069	0.004	0.134	0.101	0.981
			ln(c)	1.705	2.471	0.767	0.479	0.356	0.201
			D_{max}	7.222	15.322	8.100	1.254	1.054	0.001
		KG vs. Th	z	0.073	0.082	0.009	0.149	0.109	0.961
			ln(c)	1.705	2.516	0.812	0.537	0.391	0.218
			D_{max}	7.222	16.736	9.514	1.520	1.129	0.000
		PT vs. Sal	z	0.082	0.058	0.024	0.087	0.071	0.799
			ln(c)	2.438	2.741	0.303	0.304	0.251	0.415
			D_{max}	15.927	19.168	3.242	1.114	0.877	0.025
		PT vs. Stool	z	0.082	0.080	0.002	0.128	0.104	0.992
			ln(c)	2.438	2.004	0.435	0.471	0.378	0.450
			D_{max}	15.927	10.080	5.847	2.189	1.653	0.043
		PT vs. SubP	z	0.082	0.085	0.003	0.078	0.067	0.978
			ln(c)	2.438	2.744	0.306	0.280	0.243	0.354
			D_{max}	15.927	21.296	5.369	1.043	1.056	0.002
		PT vs. SupP	z	0.082	0.066	0.016	0.075	0.061	0.843
			ln(c)	2.438	2.673	0.235	0.274	0.219	0.494
			D_{max}	15.927	18.553	2.626	1.081	0.915	0.065
		PT vs. TD	z	0.082	0.069	0.013	0.075	0.064	0.861
			ln(c)	2.438	2.471	0.033	0.276	0.234	0.916
			D_{max}	15.927	15.322	0.605	0.910	0.704	0.592
		PT vs. Th	z	0.082	0.082	0.000	0.092	0.074	0.997
			ln(c)	2.438	2.516	0.078	0.316	0.271	0.814
			D_{max}	15.927	16.736	0.810	1.211	1.022	0.570
		Sal vs. Stool	z	0.058	0.080	0.023	0.159	0.121	0.909
			ln(c)	2.741	2.004	0.737	0.559	0.426	0.290
			D_{max}	19.168	10.080	9.089	3.063	3.697	0.044
		Sal vs. SubP	z	0.058	0.085	0.028	0.049	0.038	0.657
			ln(c)	2.741	2.744	0.003	0.173	0.134	0.983
			D_{max}	19.168	21.296	2.128	0.660	0.521	0.012
		Sal vs. SupP	z	0.058	0.066	0.009	0.058	0.045	0.903
			ln(c)	2.741	2.673	0.068	0.208	0.161	0.800
			D_{max}	19.168	18.553	0.615	0.721	0.570	0.493
		Sal vs. TD	z	0.058	0.069	0.012	0.063	0.052	0.875
			ln(c)	2.741	2.471	0.269	0.223	0.177	0.322
			D_{max}	19.168	15.322	3.846	0.726	0.589	0.003
		Sal vs. Th	z	0.058	0.082	0.025	0.077	0.062	0.783
			ln(c)	2.741	2.516	0.224	0.270	0.217	0.489
			D_{max}	19.168	16.736	2.432	0.968	0.772	0.059
		Stool vs. SubP	z	0.080	0.085	0.005	0.147	0.119	0.978

			ln(*c*)	2.004	2.744	0.741	0.538	0.426	0.255
			D_{max}	10.080	21.296	11.216	2.782	2.406	0.008
		Stool vs. SupP	*z*	0.080	0.066	0.014	0.134	0.106	0.931
			ln(*c*)	2.004	2.673	0.669	0.479	0.387	0.256
			D_{max}	10.080	18.553	8.473	2.585	2.572	0.018
		Stool vs. TD	*z*	0.080	0.069	0.011	0.122	0.092	0.960
			ln(*c*)	2.004	2.471	0.468	0.447	0.342	0.403
			D_{max}	10.080	15.322	5.242	1.868	1.705	0.045
		Stool vs. Th	*z*	0.080	0.082	0.002	0.132	0.102	0.990
			ln(*c*)	2.004	2.516	0.513	0.471	0.376	0.371
			D_{max}	10.080	16.736	6.656	2.376	2.203	0.037
		SubP vs. SupP	*z*	0.085	0.066	0.019	0.053	0.044	0.746
			ln(*c*)	2.744	2.673	0.071	0.194	0.156	0.756
			D_{max}	21.296	18.553	2.743	0.640	0.529	0.003
		SubP vs. TD	*z*	0.085	0.069	0.016	0.062	0.050	0.832
			ln(*c*)	2.744	2.471	0.273	0.219	0.177	0.308
			D_{max}	21.296	15.322	5.974	0.749	0.583	0.000
		SubP vs. Th	*z*	0.085	0.082	0.003	0.065	0.053	0.979
			ln(*c*)	2.744	2.516	0.228	0.233	0.191	0.412
			D_{max}	21.296	16.736	4.560	0.897	0.739	0.002
		SupP vs. TD	*z*	0.066	0.069	0.003	0.062	0.050	0.969
			ln(*c*)	2.673	2.471	0.202	0.223	0.180	0.435
			D_{max}	18.553	15.322	3.231	0.707	0.589	0.003
		SupP vs. Th	*z*	0.066	0.082	0.016	0.071	0.056	0.863
			ln(*c*)	2.673	2.516	0.157	0.257	0.202	0.613
			D_{max}	18.553	16.736	1.817	0.892	0.744	0.096
		TD vs. Th	*z*	0.069	0.082	0.013	0.066	0.051	0.867
			ln(*c*)	2.471	2.516	0.045	0.237	0.182	0.885
			D_{max}	15.322	16.736	1.414	0.727	0.568	0.111
q=2	PL	BM vs. HP	*z*	-0.007	-0.013	0.005	0.076	0.057	0.952
			ln(*c*)	1.221	1.504	0.283	0.383	0.281	0.563
		BM vs. KG	*z*	-0.007	0.001	0.009	0.076	0.058	0.919
			ln(*c*)	1.221	1.223	0.003	0.384	0.288	0.996
		BM vs. PT	*z*	-0.007	0.038	0.045	0.101	0.071	0.710
			ln(*c*)	1.221	1.945	0.725	0.508	0.363	0.260
		BM vs. Sal	*z*	-0.007	0.028	0.035	0.115	0.085	0.780
			ln(*c*)	1.221	2.328	1.107	0.584	0.417	0.124
		BM vs. Stool	*z*	-0.007	-0.006	0.001	0.083	0.063	0.989
			ln(*c*)	1.221	1.455	0.235	0.416	0.313	0.654
		BM vs. SubP	*z*	-0.007	0.051	0.058	0.113	0.083	0.683
			ln(*c*)	1.221	2.434	1.214	0.570	0.423	0.104
		BM vs. SupP	*z*	-0.007	0.044	0.051	0.104	0.077	0.694
			ln(*c*)	1.221	2.346	1.126	0.529	0.389	0.086
		BM vs. TD	*z*	-0.007	0.042	0.049	0.099	0.072	0.694
			ln(*c*)	1.221	2.122	0.901	0.510	0.367	0.156
		BM vs. Th	*z*	-0.007	0.021	0.028	0.091	0.068	0.800
			ln(*c*)	1.221	2.043	0.823	0.459	0.341	0.165
		HP vs. KG	*z*	-0.013	0.001	0.014	0.086	0.062	0.899
			ln(*c*)	1.504	1.223	0.281	0.430	0.308	0.619
		HP vs. PT	*z*	-0.013	0.038	0.051	0.097	0.068	0.683
			ln(*c*)	1.504	1.945	0.442	0.496	0.345	0.514
		HP vs. Sal	*z*	-0.013	0.028	0.041	0.096	0.072	0.726
			ln(*c*)	1.504	2.328	0.824	0.486	0.358	0.163
		HP vs. Stool	*z*	-0.013	-0.006	0.006	0.086	0.067	0.958
			ln(*c*)	1.504	1.455	0.048	0.432	0.340	0.940
		HP vs. SubP	*z*	-0.013	0.051	0.063	0.106	0.078	0.630
			ln(*c*)	1.504	2.434	0.931	0.531	0.393	0.174
		HP vs. SupP	*z*	-0.013	0.044	0.057	0.098	0.071	0.651
			ln(*c*)	1.504	2.346	0.843	0.504	0.366	0.176
		HP vs. TD	*z*	-0.013	0.042	0.055	0.091	0.065	0.668
			ln(*c*)	1.504	2.122	0.618	0.462	0.328	0.276
		HP vs. Th	*z*	-0.013	0.021	0.033	0.090	0.063	0.779
			ln(*c*)	1.504	2.043	0.540	0.449	0.322	0.376
		KG vs. PT	*z*	0.001	0.038	0.037	0.112	0.078	0.791
			ln(*c*)	1.223	1.945	0.722	0.571	0.399	0.342
		KG vs. Sal	*z*	0.001	0.028	0.027	0.114	0.085	0.825
			ln(*c*)	1.223	2.328	1.105	0.575	0.420	0.133
		KG vs. Stool	*z*	0.001	-0.006	0.008	0.085	0.068	0.936
			ln(*c*)	1.223	1.455	0.232	0.433	0.348	0.661
		KG vs. SubP	*z*	0.001	0.051	0.049	0.117	0.086	0.730
			ln(*c*)	1.223	2.434	1.211	0.593	0.435	0.107

		KG vs. SupP	z	0.001	0.044	0.043	0.114	0.083	0.759
			$\ln(c)$	1.223	2.346	1.123	0.578	0.411	0.115
		KG vs. TD	z	0.001	0.042	0.041	0.102	0.074	0.756
			$\ln(c)$	1.223	2.122	0.899	0.510	0.370	0.161
		KG vs. Th	z	0.001	0.021	0.019	0.104	0.074	0.893
			$\ln(c)$	1.223	2.043	0.820	0.534	0.383	0.208
		PT vs. Sal	z	0.038	0.028	0.010	0.072	0.057	0.883
			$\ln(c)$	1.945	2.328	0.382	0.363	0.282	0.402
		PT vs. Stool	z	0.038	-0.006	0.044	0.109	0.083	0.750
			$\ln(c)$	1.945	1.455	0.490	0.552	0.411	0.488
		PT vs. SubP	z	0.038	0.051	0.012	0.068	0.055	0.862
			$\ln(c)$	1.945	2.434	0.489	0.344	0.273	0.246
		PT vs. SupP	z	0.038	0.044	0.006	0.068	0.052	0.939
			$\ln(c)$	1.945	2.346	0.401	0.348	0.261	0.347
		PT vs. TD	z	0.038	0.042	0.004	0.066	0.053	0.953
			$\ln(c)$	1.945	2.122	0.177	0.335	0.268	0.662
		PT vs. Th	z	0.038	0.021	0.017	0.078	0.060	0.843
			$\ln(c)$	1.945	2.043	0.098	0.395	0.299	0.834
		Sal vs. Stool	z	0.028	-0.006	0.034	0.136	0.095	0.846
			$\ln(c)$	2.328	1.455	0.872	0.671	0.475	0.297
		Sal vs. SubP	z	0.028	0.051	0.022	0.034	0.026	0.599
			$\ln(c)$	2.328	2.434	0.107	0.169	0.129	0.603
		Sal vs. SupP	z	0.028	0.044	0.016	0.037	0.030	0.710
			$\ln(c)$	2.328	2.346	0.019	0.188	0.149	0.932
		Sal vs. TD	z	0.028	0.042	0.014	0.043	0.034	0.790
			$\ln(c)$	2.328	2.122	0.206	0.216	0.168	0.440
		Sal vs. Th	z	0.028	0.021	0.007	0.064	0.050	0.928
			$\ln(c)$	2.328	2.043	0.284	0.319	0.246	0.462
		Stool vs. SubP	z	-0.006	0.051	0.057	0.138	0.102	0.736
			$\ln(c)$	1.455	2.434	0.979	0.694	0.500	0.254
		Stool vs. SupP	z	-0.006	0.044	0.050	0.130	0.096	0.761
			$\ln(c)$	1.455	2.346	0.891	0.647	0.486	0.278
		Stool vs. TD	z	-0.006	0.042	0.049	0.111	0.081	0.737
			$\ln(c)$	1.455	2.122	0.666	0.572	0.424	0.359
		Stool vs. Th	z	-0.006	0.021	0.027	0.106	0.082	0.844
			$\ln(c)$	1.455	2.043	0.588	0.550	0.420	0.385
		SubP vs. SupP	z	0.051	0.044	0.007	0.034	0.026	0.855
			$\ln(c)$	2.434	2.346	0.088	0.169	0.132	0.660
		SubP vs. TD	z	0.051	0.042	0.008	0.042	0.033	0.868
			$\ln(c)$	2.434	2.122	0.312	0.214	0.167	0.247
		SubP vs. Th	z	0.051	0.021	0.030	0.060	0.049	0.679
			$\ln(c)$	2.434	2.043	0.391	0.300	0.247	0.294
		SupP vs. TD	z	0.044	0.042	0.002	0.046	0.037	0.973
			$\ln(c)$	2.346	2.122	0.224	0.233	0.184	0.441
		SupP vs. Th	z	0.044	0.021	0.023	0.064	0.048	0.763
			$\ln(c)$	2.346	2.043	0.303	0.324	0.244	0.470
		TD vs. Th	z	0.042	0.021	0.021	0.058	0.045	0.758
			$\ln(c)$	2.122	2.043	0.078	0.297	0.228	0.840
	PLEC	BM vs. HP	z	-0.006	-0.014	0.008	0.142	0.103	0.972
			$\ln(c)$	1.215	1.503	0.288	0.487	0.363	0.644
			D_{max}	NaN	4.215	NA	0.411	0.326	1.000
		BM vs. KG	z	-0.006	0.007	0.013	0.142	0.102	0.952
			$\ln(c)$	1.215	1.211	0.004	0.491	0.361	0.997
			D_{max}	NaN	3.433	NA	0.397	0.287	1.000
		BM vs. PT	z	-0.006	0.047	0.053	0.184	0.125	0.836
			$\ln(c)$	1.215	1.935	0.720	0.621	0.421	0.382
			D_{max}	NaN	8.472	NA	0.829	0.688	1.000
		BM vs. Sal	z	-0.006	0.050	0.056	0.194	0.140	0.823
			$\ln(c)$	1.215	2.280	1.066	0.655	0.484	0.209
			D_{max}	NaN	11.749	NA	1.073	0.837	1.000
		BM vs. Stool	z	-0.006	0.003	0.009	0.145	0.115	0.958
			$\ln(c)$	1.215	1.431	0.216	0.518	0.395	0.736
			D_{max}	NaN	4.205	NA	0.937	0.830	1.000
		BM vs. SubP	z	-0.006	0.101	0.107	0.193	0.142	0.667
			$\ln(c)$	1.215	2.311	1.096	0.653	0.480	0.188
			D_{max}	NaN	14.648	NA	1.415	1.067	1.000
		BM vs. SupP	z	-0.006	0.077	0.084	0.183	0.133	0.722
			$\ln(c)$	1.215	2.271	1.056	0.628	0.455	0.169
			D_{max}	NaN	12.982	NA	1.283	0.957	1.000
		BM vs. TD	z	-0.006	0.074	0.080	0.172	0.122	0.716
			$\ln(c)$	1.215	2.051	0.836	0.591	0.429	0.268

			D_{max}	NaN	10.284	NA	0.996	0.732	1.000
		BM vs. Th	z	-0.006	0.054	0.060	0.160	0.117	0.752
			$\ln(c)$	1.215	1.968	0.753	0.533	0.391	0.276
			D_{max}	NaN	8.607	NA	0.760	0.594	1.000
		HP vs. KG	z	-0.014	0.007	0.021	0.152	0.112	0.923
			$\ln(c)$	1.503	1.211	0.293	0.526	0.385	0.676
			D_{max}	4.215	3.433	0.782	0.479	0.394	0.191
		HP vs. PT	z	-0.014	0.047	0.061	0.163	0.120	0.759
			$\ln(c)$	1.503	1.935	0.432	0.555	0.415	0.542
			D_{max}	4.215	8.472	4.257	1.019	0.838	0.003
		HP vs. Sal	z	-0.014	0.050	0.064	0.182	0.125	0.798
			$\ln(c)$	1.503	2.280	0.777	0.590	0.433	0.297
			D_{max}	4.215	11.749	7.534	1.208	0.924	0.000
		HP vs. Stool	z	-0.014	0.003	0.017	0.152	0.118	0.916
			$\ln(c)$	1.503	1.431	0.073	0.540	0.405	0.913
			D_{max}	4.215	4.205	0.010	1.114	0.944	0.989
		HP vs. SubP	z	-0.014	0.101	0.115	0.179	0.131	0.607
			$\ln(c)$	1.503	2.311	0.808	0.598	0.465	0.285
			D_{max}	4.215	14.648	10.433	1.540	1.232	0.002
		HP vs. SupP	z	-0.014	0.077	0.092	0.169	0.125	0.677
			$\ln(c)$	1.503	2.271	0.768	0.549	0.424	0.259
			D_{max}	4.215	12.982	8.768	1.466	1.150	0.000
		HP vs. TD	z	-0.014	0.074	0.088	0.162	0.116	0.676
			$\ln(c)$	1.503	2.051	0.548	0.543	0.411	0.433
			D_{max}	4.215	10.284	6.069	1.065	0.831	0.000
		HP vs. Th	z	-0.014	0.054	0.068	0.165	0.113	0.754
			$\ln(c)$	1.503	1.968	0.465	0.546	0.383	0.527
			D_{max}	4.215	8.607	4.392	0.884	0.727	0.002
		KG vs. PT	z	0.007	0.047	0.040	0.196	0.140	0.856
			$\ln(c)$	1.211	1.935	0.724	0.671	0.486	0.408
			D_{max}	3.433	8.472	5.039	0.953	0.728	0.000
		KG vs. Sal	z	0.007	0.050	0.043	0.214	0.154	0.885
			$\ln(c)$	1.211	2.280	1.070	0.695	0.515	0.222
			D_{max}	3.433	11.749	8.316	1.204	0.980	0.000
		KG vs. Stool	z	0.007	0.003	0.004	0.154	0.119	0.986
			$\ln(c)$	1.211	1.431	0.220	0.551	0.415	0.750
			D_{max}	3.433	4.205	0.772	1.056	0.868	0.557
		KG vs. SubP	z	0.007	0.101	0.094	0.214	0.145	0.754
			$\ln(c)$	1.211	2.311	1.101	0.707	0.497	0.204
			D_{max}	3.433	14.648	11.215	1.554	1.198	0.000
		KG vs. SupP	z	0.007	0.077	0.070	0.194	0.136	0.783
			$\ln(c)$	1.211	2.271	1.061	0.651	0.483	0.204
			D_{max}	3.433	12.982	9.549	1.459	1.331	0.002
		KG vs. TD	z	0.007	0.074	0.067	0.180	0.127	0.770
			$\ln(c)$	1.211	2.051	0.840	0.618	0.436	0.275
			D_{max}	3.433	10.284	6.851	1.094	1.852	0.002
		KG vs. Th	z	0.007	0.054	0.047	0.178	0.127	0.849
			$\ln(c)$	1.211	1.968	0.757	0.605	0.450	0.321
			D_{max}	3.433	8.607	5.174	0.944	0.770	0.003
		PT vs. Sal	z	0.047	0.050	0.003	0.126	0.098	0.986
			$\ln(c)$	1.935	2.280	0.345	0.420	0.361	0.494
			D_{max}	8.472	11.749	3.277	1.169	0.893	0.029
		PT vs. Stool	z	0.047	0.003	0.044	0.184	0.145	0.817
			$\ln(c)$	1.935	1.431	0.504	0.623	0.487	0.504
			D_{max}	8.472	4.205	4.267	2.087	1.797	0.113
		PT vs. SubP	z	0.047	0.101	0.054	0.122	0.098	0.722
			$\ln(c)$	1.935	2.311	0.376	0.422	0.365	0.415
			D_{max}	8.472	14.648	6.176	1.233	0.997	0.000
		PT vs. SupP	z	0.047	0.077	0.031	0.119	0.091	0.837
			$\ln(c)$	1.935	2.271	0.336	0.423	0.336	0.528
			D_{max}	8.472	12.982	4.511	1.356	1.076	0.009
		PT vs. TD	z	0.047	0.074	0.027	0.114	0.094	0.832
			$\ln(c)$	1.935	2.051	0.116	0.411	0.353	0.797
			D_{max}	8.472	10.284	1.812	0.952	0.777	0.134
		PT vs. Th	z	0.047	0.054	0.007	0.145	0.106	0.968
			$\ln(c)$	1.935	1.968	0.033	0.477	0.378	0.953
			D_{max}	8.472	8.607	0.135	1.221	1.031	0.924
		Sal vs. Stool	z	0.050	0.003	0.047	0.226	0.160	0.887
			$\ln(c)$	2.280	1.431	0.850	0.713	0.517	0.317
			D_{max}	11.749	4.205	7.544	2.708	2.457	0.027
		Sal vs. SubP	z	0.050	0.101	0.051	0.063	0.049	0.515

			ln(c)	2.280	2.311	0.031	0.226	0.173	0.916
			D_{max}	11.749	14.648	2.899	0.606	0.480	0.003
		Sal vs. SupP	z	0.050	0.077	0.028	0.069	0.055	0.750
			ln(c)	2.280	2.271	0.009	0.251	0.198	0.971
			D_{max}	11.749	12.982	1.233	0.729	0.641	0.172
		Sal vs. TD	z	0.050	0.074	0.024	0.080	0.067	0.790
			ln(c)	2.280	2.051	0.229	0.280	0.233	0.470
			D_{max}	11.749	10.284	1.465	0.696	0.539	0.103
		Sal vs. Th	z	0.050	0.054	0.004	0.114	0.091	0.978
			ln(c)	2.280	1.968	0.313	0.387	0.323	0.490
			D_{max}	11.749	8.607	3.142	1.067	0.833	0.024
		Stool vs. SubP	z	0.003	0.101	0.098	0.220	0.166	0.716
			ln(c)	1.431	2.311	0.881	0.731	0.537	0.335
			D_{max}	4.205	14.648	10.443	3.135	2.306	0.005
		Stool vs. SupP	z	0.003	0.077	0.075	0.209	0.156	0.776
			ln(c)	1.431	2.271	0.841	0.671	0.522	0.321
			D_{max}	4.205	12.982	8.777	3.102	2.300	0.021
		Stool vs. TD	z	0.003	0.074	0.071	0.182	0.136	0.763
			ln(c)	1.431	2.051	0.620	0.613	0.450	0.404
			D_{max}	4.205	10.284	6.079	2.278	1.772	0.037
		Stool vs. Th	z	0.003	0.054	0.051	0.180	0.133	0.810
			ln(c)	1.431	1.968	0.537	0.587	0.450	0.451
			D_{max}	4.205	8.607	4.402	2.140	1.613	0.100
		SubP vs. SupP	z	0.101	0.077	0.023	0.063	0.050	0.754
			ln(c)	2.311	2.271	0.040	0.231	0.183	0.881
			D_{max}	14.648	12.982	1.666	0.529	0.465	0.021
		SubP vs. TD	z	0.101	0.074	0.027	0.081	0.067	0.776
			ln(c)	2.311	2.051	0.260	0.287	0.238	0.439
			D_{max}	14.648	10.284	4.364	0.803	0.728	0.001
		SubP vs. Th	z	0.101	0.054	0.047	0.107	0.080	0.730
			ln(c)	2.311	1.968	0.344	0.372	0.293	0.434
			D_{max}	14.648	8.607	6.041	1.204	0.951	0.001
		SupP vs. TD	z	0.077	0.074	0.004	0.084	0.067	0.974
			ln(c)	2.271	2.051	0.220	0.301	0.242	0.540
			D_{max}	12.982	10.284	2.699	0.845	0.690	0.012
		SupP vs. Th	z	0.077	0.054	0.024	0.113	0.084	0.883
			ln(c)	2.271	1.968	0.304	0.394	0.300	0.539
			D_{max}	12.982	8.607	4.376	1.214	0.951	0.010
		TD vs. Th	z	0.074	0.054	0.020	0.100	0.077	0.863
			ln(c)	2.051	1.968	0.083	0.357	0.279	0.838
			D_{max}	10.284	8.607	1.677	0.859	0.658	0.110
q=3	PL	BM vs. HP	z	-0.011	-0.019	0.008	0.067	0.050	0.921
			ln(c)	0.985	1.232	0.247	0.339	0.248	0.575
		BM vs. KG	z	-0.011	-0.008	0.003	0.067	0.051	0.967
			ln(c)	0.985	1.015	0.030	0.340	0.253	0.949
		BM vs. PT	z	-0.011	0.020	0.031	0.098	0.069	0.791
			ln(c)	0.985	1.729	0.744	0.494	0.353	0.236
		BM vs. Sal	z	-0.011	0.027	0.038	0.116	0.085	0.780
			ln(c)	0.985	2.137	1.152	0.588	0.421	0.113
		BM vs. Stool	z	-0.011	-0.019	0.008	0.078	0.060	0.935
			ln(c)	0.985	1.219	0.235	0.393	0.300	0.629
		BM vs. SubP	z	-0.011	0.058	0.069	0.119	0.086	0.646
			ln(c)	0.985	2.244	1.259	0.595	0.436	0.103
		BM vs. SupP	z	-0.011	0.046	0.057	0.109	0.079	0.691
			ln(c)	0.985	2.176	1.192	0.560	0.396	0.078
		BM vs. TD	z	-0.011	0.040	0.051	0.100	0.074	0.671
			ln(c)	0.985	1.929	0.944	0.516	0.376	0.143
		BM vs. Th	z	-0.011	0.005	0.016	0.087	0.065	0.872
			ln(c)	0.985	1.812	0.827	0.439	0.325	0.142
		HP vs. KG	z	-0.019	-0.008	0.011	0.076	0.055	0.902
			ln(c)	1.232	1.015	0.217	0.376	0.269	0.650
		HP vs. PT	z	-0.019	0.020	0.039	0.098	0.068	0.761
			ln(c)	1.232	1.729	0.497	0.503	0.346	0.464
		HP vs. Sal	z	-0.019	0.027	0.046	0.101	0.075	0.711
			ln(c)	1.232	2.137	0.905	0.512	0.372	0.143
		HP vs. Stool	z	-0.019	-0.019	0.000	0.084	0.066	1.000
			ln(c)	1.232	1.219	0.013	0.420	0.333	0.984
		HP vs. SubP	z	-0.019	0.058	0.077	0.115	0.083	0.591
			ln(c)	1.232	2.244	1.012	0.577	0.423	0.174
		HP vs. SupP	z	-0.019	0.046	0.065	0.107	0.078	0.618
			ln(c)	1.232	2.176	0.944	0.552	0.399	0.175

		HP vs. TD	z	-0.019	0.040	0.059	0.098	0.069	0.648
			ln(c)	1.232	1.929	0.697	0.500	0.353	0.276
		HP vs. Th	z	-0.019	0.005	0.024	0.090	0.062	0.828
			ln(c)	1.232	1.812	0.580	0.448	0.317	0.311
		KG vs. PT	z	-0.008	0.020	0.028	0.107	0.074	0.839
			ln(c)	1.015	1.729	0.714	0.544	0.379	0.317
		KG vs. Sal	z	-0.008	0.027	0.034	0.115	0.085	0.790
			ln(c)	1.015	2.137	1.122	0.577	0.416	0.117
		KG vs. Stool	z	-0.008	-0.019	0.011	0.080	0.065	0.885
			ln(c)	1.015	1.219	0.204	0.409	0.329	0.682
		KG vs. SubP	z	-0.008	0.058	0.066	0.118	0.085	0.669
			ln(c)	1.015	2.244	1.229	0.597	0.430	0.100
		KG vs. SupP	z	-0.008	0.046	0.054	0.114	0.084	0.710
			ln(c)	1.015	2.176	1.161	0.588	0.416	0.110
		KG vs. TD	z	-0.008	0.040	0.048	0.105	0.075	0.727
			ln(c)	1.015	1.929	0.914	0.526	0.371	0.158
		KG vs. Th	z	-0.008	0.005	0.013	0.099	0.071	0.908
			ln(c)	1.015	1.812	0.797	0.508	0.366	0.196
		PT vs. Sal	z	0.020	0.027	0.006	0.084	0.065	0.940
			ln(c)	1.729	2.137	0.408	0.430	0.325	0.452
		PT vs. Stool	z	0.020	-0.019	0.040	0.117	0.089	0.766
			ln(c)	1.729	1.219	0.509	0.588	0.432	0.505
		PT vs. SubP	z	0.020	0.058	0.037	0.084	0.063	0.722
			ln(c)	1.729	2.244	0.515	0.425	0.320	0.327
		PT vs. SupP	z	0.020	0.046	0.026	0.087	0.064	0.809
			ln(c)	1.729	2.176	0.447	0.446	0.321	0.428
		PT vs. TD	z	0.020	0.040	0.020	0.081	0.063	0.817
			ln(c)	1.729	1.929	0.200	0.414	0.313	0.695
		PT vs. Th	z	0.020	0.005	0.015	0.088	0.066	0.885
			ln(c)	1.729	1.812	0.083	0.447	0.335	0.878
		Sal vs. Stool	z	0.027	-0.019	0.046	0.143	0.099	0.808
			ln(c)	2.137	1.219	0.917	0.711	0.493	0.330
		Sal vs. SubP	z	0.027	0.058	0.031	0.039	0.030	0.541
			ln(c)	2.137	2.244	0.107	0.198	0.146	0.689
		Sal vs. SupP	z	0.027	0.046	0.020	0.044	0.035	0.709
			ln(c)	2.137	2.176	0.040	0.224	0.179	0.875
		Sal vs. TD	z	0.027	0.040	0.014	0.051	0.040	0.818
			ln(c)	2.137	1.929	0.208	0.254	0.198	0.513
		Sal vs. Th	z	0.027	0.005	0.021	0.075	0.058	0.811
			ln(c)	2.137	1.812	0.325	0.375	0.286	0.477
		Stool vs. SubP	z	-0.019	0.058	0.077	0.150	0.108	0.701
			ln(c)	1.219	2.244	1.025	0.755	0.529	0.285
		Stool vs. SupP	z	-0.019	0.046	0.066	0.147	0.107	0.709
			ln(c)	1.219	2.176	0.957	0.737	0.539	0.327
		Stool vs. TD	z	-0.019	0.040	0.059	0.129	0.089	0.741
			ln(c)	1.219	1.929	0.709	0.647	0.466	0.412
		Stool vs. Th	z	-0.019	0.005	0.025	0.114	0.085	0.847
			ln(c)	1.219	1.812	0.592	0.581	0.431	0.415
		SubP vs. SupP	z	0.058	0.046	0.011	0.038	0.029	0.806
			ln(c)	2.244	2.176	0.068	0.193	0.148	0.775
		SubP vs. TD	z	0.058	0.040	0.018	0.051	0.041	0.763
			ln(c)	2.244	1.929	0.316	0.263	0.206	0.334
		SubP vs. Th	z	0.058	0.005	0.052	0.079	0.061	0.583
			ln(c)	2.244	1.812	0.432	0.396	0.314	0.391
		SupP vs. TD	z	0.046	0.040	0.006	0.058	0.045	0.934
			ln(c)	2.176	1.929	0.248	0.293	0.224	0.497
		SupP vs. Th	z	0.046	0.005	0.041	0.079	0.059	0.678
			ln(c)	2.176	1.812	0.365	0.405	0.303	0.470
		TD vs. Th	z	0.040	0.005	0.035	0.072	0.053	0.713
			ln(c)	1.929	1.812	0.117	0.370	0.269	0.801
	PLEC	BM vs. HP	z	-0.016	-0.028	0.012	0.128	0.092	0.945
			ln(c)	0.995	1.248	0.253	0.441	0.324	0.661
			D_{max}	2.533	3.117	0.584	0.260	0.212	0.071
		BM vs. KG	z	-0.016	-0.011	0.005	0.130	0.092	0.973
			ln(c)	0.995	1.022	0.027	0.447	0.326	0.967
			D_{max}	2.533	2.656	0.123	0.259	0.188	0.737
		BM vs. PT	z	-0.016	0.019	0.035	0.180	0.119	0.886
			ln(c)	0.995	1.737	0.742	0.615	0.410	0.370
			D_{max}	2.533	6.503	3.970	0.557	0.479	0.000
		BM vs. Sal	z	-0.016	0.046	0.063	0.193	0.138	0.801
			ln(c)	0.995	2.095	1.100	0.659	0.474	0.184

			D_{max}	2.533	9.634	7.101	0.768	0.683	0.002
		BM vs. Stool	z	-0.016	-0.021	0.005	0.137	0.111	0.988
			ln(c)	0.995	1.218	0.223	0.488	0.375	0.710
			D_{max}	2.533	3.061	0.528	0.653	0.549	0.495
		BM vs. SubP	z	-0.016	0.111	0.127	0.200	0.147	0.619
			ln(c)	0.995	2.113	1.119	0.678	0.491	0.186
			D_{max}	2.533	12.539	10.006	0.962	0.785	0.000
		BM vs. SupP	z	-0.016	0.082	0.098	0.189	0.138	0.676
			ln(c)	0.995	2.095	1.100	0.642	0.468	0.151
			D_{max}	2.533	11.082	8.548	0.964	0.767	0.000
		BM vs. TD	z	-0.016	0.068	0.084	0.178	0.126	0.721
			ln(c)	0.995	1.868	0.873	0.616	0.443	0.270
			D_{max}	2.533	8.391	5.858	0.698	0.552	0.000
		BM vs. Th	z	-0.016	0.032	0.048	0.154	0.114	0.787
			ln(c)	0.995	1.750	0.755	0.518	0.388	0.263
			D_{max}	2.533	6.356	3.823	0.487	0.366	0.000
		HP vs. KG	z	-0.028	-0.011	0.017	0.138	0.099	0.942
			ln(c)	1.248	1.022	0.226	0.477	0.345	0.737
			D_{max}	3.117	2.656	0.462	0.289	0.233	0.191
		HP vs. PT	z	-0.028	0.019	0.047	0.163	0.122	0.799
			ln(c)	1.248	1.737	0.489	0.567	0.420	0.506
			D_{max}	3.117	6.503	3.385	0.701	0.586	0.002
		HP vs. Sal	z	-0.028	0.046	0.075	0.187	0.129	0.780
			ln(c)	1.248	2.095	0.847	0.611	0.439	0.263
			D_{max}	3.117	9.634	6.516	0.917	0.717	0.000
		HP vs. Stool	z	-0.028	-0.021	0.007	0.150	0.115	0.958
			ln(c)	1.248	1.218	0.030	0.522	0.392	0.968
			D_{max}	3.117	3.061	0.057	0.846	0.748	0.947
		HP vs. SubP	z	-0.028	0.111	0.139	0.192	0.142	0.573
			ln(c)	1.248	2.113	0.865	0.647	0.487	0.290
			D_{max}	3.117	12.539	9.422	1.248	1.407	0.002
		HP vs. SupP	z	-0.028	0.082	0.110	0.180	0.135	0.634
			ln(c)	1.248	2.095	0.847	0.578	0.453	0.247
			D_{max}	3.117	11.082	7.964	1.173	1.134	0.002
		HP vs. TD	z	-0.028	0.068	0.097	0.172	0.121	0.674
			ln(c)	1.248	1.868	0.620	0.575	0.422	0.407
			D_{max}	3.117	8.391	5.273	0.852	0.678	0.000
		HP vs. Th	z	-0.028	0.032	0.060	0.166	0.114	0.789
			ln(c)	1.248	1.750	0.501	0.557	0.379	0.494
			D_{max}	3.117	6.356	3.239	0.626	0.537	0.002
		KG vs. PT	z	-0.011	0.019	0.030	0.190	0.133	0.894
			ln(c)	1.022	1.737	0.715	0.662	0.461	0.416
			D_{max}	2.656	6.503	3.847	0.625	0.488	0.000
		KG vs. Sal	z	-0.011	0.046	0.057	0.215	0.153	0.829
			ln(c)	1.022	2.095	1.073	0.703	0.518	0.225
			D_{max}	2.656	9.634	6.978	0.830	0.694	0.000
		KG vs. Stool	z	-0.011	-0.021	0.010	0.149	0.116	0.964
			ln(c)	1.022	1.218	0.196	0.528	0.398	0.783
			D_{max}	2.656	3.061	0.405	0.729	0.578	0.656
		KG vs. SubP	z	-0.011	0.111	0.122	0.216	0.145	0.685
			ln(c)	1.022	2.113	1.091	0.717	0.502	0.216
			D_{max}	2.656	12.539	9.883	1.058	0.842	0.000
		KG vs. SupP	z	-0.011	0.082	0.093	0.198	0.143	0.728
			ln(c)	1.022	2.095	1.073	0.649	0.503	0.203
			D_{max}	2.656	11.082	8.426	1.034	0.806	0.000
		KG vs. TD	z	-0.011	0.068	0.079	0.182	0.127	0.742
			ln(c)	1.022	1.868	0.846	0.619	0.430	0.269
			D_{max}	2.656	8.391	5.735	0.763	0.616	0.000
		KG vs. Th	z	-0.011	0.032	0.043	0.170	0.123	0.850
			ln(c)	1.022	1.750	0.727	0.582	0.435	0.312
			D_{max}	2.656	6.356	3.701	0.589	0.459	0.000
		PT vs. Sal	z	0.019	0.046	0.028	0.145	0.106	0.899
			ln(c)	1.737	2.095	0.358	0.473	0.393	0.533
			D_{max}	6.503	9.634	3.131	1.204	0.908	0.040
		PT vs. Stool	z	0.019	-0.021	0.039	0.190	0.150	0.856
			ln(c)	1.737	1.218	0.519	0.620	0.490	0.496
			D_{max}	6.503	3.061	3.442	1.725	1.241	0.110
		PT vs. SubP	z	0.019	0.111	0.093	0.145	0.110	0.609
			ln(c)	1.737	2.113	0.376	0.478	0.397	0.483
			D_{max}	6.503	12.539	6.037	1.350	1.060	0.003
		PT vs. SupP	z	0.019	0.082	0.063	0.143	0.106	0.736

			ln(c)	1.737	2.095	0.358	0.478	0.371	0.538
			D_{max}	6.503	11.082	4.579	1.499	1.146	0.008
		PT vs. TD	z	0.019	0.068	0.050	0.134	0.103	0.764
			ln(c)	1.737	1.868	0.131	0.470	0.381	0.803
			D_{max}	6.503	8.391	1.888	1.009	0.846	0.125
		PT vs. Th	z	0.019	0.032	0.013	0.158	0.115	0.956
			ln(c)	1.737	1.750	0.012	0.512	0.404	0.982
			D_{max}	6.503	6.356	0.146	1.027	0.817	0.910
		Sal vs. Stool	z	0.046	-0.021	0.067	0.239	0.166	0.832
			ln(c)	2.095	1.218	0.877	0.741	0.529	0.346
			D_{max}	9.634	3.061	6.573	1.918	1.450	0.010
		Sal vs. SubP	z	0.046	0.111	0.065	0.070	0.053	0.443
			ln(c)	2.095	2.113	0.019	0.252	0.188	0.951
			D_{max}	9.634	12.539	2.906	0.676	0.554	0.005
		Sal vs. SupP	z	0.046	0.082	0.035	0.078	0.063	0.691
			ln(c)	2.095	2.095	0.000	0.283	0.221	1.000
			D_{max}	9.634	11.082	1.448	0.894	0.774	0.173
		Sal vs. TD	z	0.046	0.068	0.022	0.093	0.076	0.839
			ln(c)	2.095	1.868	0.227	0.316	0.266	0.529
			D_{max}	9.634	8.391	1.243	0.737	0.560	0.174
		Sal vs. Th	z	0.046	0.032	0.015	0.132	0.104	0.932
			ln(c)	2.095	1.750	0.345	0.443	0.365	0.513
			D_{max}	9.634	6.356	3.277	1.072	0.893	0.017
		Stool vs. SubP	z	-0.021	0.111	0.132	0.240	0.176	0.664
			ln(c)	1.218	2.113	0.896	0.774	0.548	0.360
			D_{max}	3.061	12.539	9.479	2.643	1.928	0.003
		Stool vs. SupP	z	-0.021	0.082	0.103	0.234	0.166	0.753
			ln(c)	1.218	2.095	0.877	0.714	0.535	0.338
			D_{max}	3.061	11.082	8.021	2.663	1.868	0.011
		Stool vs. TD	z	-0.021	0.068	0.089	0.205	0.150	0.755
			ln(c)	1.218	1.868	0.650	0.659	0.460	0.450
			D_{max}	3.061	8.391	5.330	2.151	1.596	0.042
		Stool vs. Th	z	-0.021	0.032	0.052	0.188	0.136	0.824
			ln(c)	1.218	1.750	0.532	0.584	0.443	0.449
			D_{max}	3.061	6.356	3.296	1.788	1.299	0.136
		SubP vs. SupP	z	0.111	0.082	0.029	0.070	0.056	0.722
			ln(c)	2.113	2.095	0.018	0.258	0.203	0.951
			D_{max}	12.539	11.082	1.458	0.572	0.502	0.052
		SubP vs. TD	z	0.111	0.068	0.043	0.094	0.076	0.705
			ln(c)	2.113	1.868	0.246	0.323	0.270	0.503
			D_{max}	12.539	8.391	4.148	0.900	0.742	0.003
		SubP vs. Th	z	0.111	0.032	0.080	0.130	0.097	0.614
			ln(c)	2.113	1.750	0.364	0.434	0.339	0.490
			D_{max}	12.539	6.356	6.183	1.350	1.015	0.000
		SupP vs. TD	z	0.082	0.068	0.013	0.100	0.079	0.919
			ln(c)	2.095	1.868	0.227	0.351	0.283	0.586
			D_{max}	11.082	8.391	2.691	0.967	0.777	0.031
		SupP vs. Th	z	0.082	0.032	0.050	0.133	0.100	0.777
			ln(c)	2.095	1.750	0.346	0.453	0.345	0.536
			D_{max}	11.082	6.356	4.725	1.354	1.020	0.011
		TD vs. Th	z	0.068	0.032	0.037	0.119	0.089	0.790
			ln(c)	1.868	1.750	0.118	0.410	0.318	0.805
			D_{max}	8.391	6.356	2.034	0.926	0.716	0.068
